# Modelling anti-Ov16 seroprevalence for the control and elimination of onchocerciasis

**DOI:** 10.1038/s41467-026-76562-9

**Published:** 2026-08-19

**Authors:** Aditya Ramani, Jacob N. Stapley, Matthew A. Dixon, Jonathan I. D. Hamley, Luís-Jorge Amaral, Maria-Gloria Basáñez, Martin Walker

**Affiliations:** 1 Department of Pathobiology and Population Sciences, Royal Veterinary College, Hawkshead Lane, Hatfield, United Kingdom; 2 MRC Centre for Global Infectious Disease Analysis, Department of Infectious Disease Epidemiology, School of Public Health, Imperial College London, London, United Kingdom; 3 Department of Visceral Surgery and Medicine, Inselspital, Bern University Hospital, University of Bern, Bern, Switzerland; 4 Multidisciplinary Center for Infectious Diseases, University of Bern, Bern, Switzerland; 5 Global Health Institute, University of Antwerp, Antwerp, Belgium

**Keywords:** Parasitic infection, Computational models, Epidemiology

## Abstract

The World Health Organization aims to verify elimination of onchocerciasis transmission in 12 endemic countries by 2030. Serological assays detecting IgG4 antibodies against the *Onchocerca volvulus* Ov16 antigen guide decision-making regarding start- and stop-mass ivermectin treatment, post-treatment and post-elimination surveillance. Transmission models are crucial for understanding anti-Ov16 IgG4 seroprevalence dynamics. Uncertainty associated with the parasite stage(s) stimulating seroconversion and duration of antibody persistence has hitherto hindered progress. We use the individual-based stochastic transmission model, EPIONCHO-IBM, to test hypotheses regarding the timing of seroconversion and seroreversion. We fit the model to data from (treatment-naïve) Gabon and validate it using data from Togo (four decades of control). The best-fit hypothesis is that seroconversion is elicited by near-patent/patent infection. The duration of antibody persistence remains uncertain. Our findings support the use of mechanistic transmission models to refine serological thresholds for start- and stop-treatment, and aid interpretation of post-treatment and post-elimination surveillance serological signals.

## Abstract (web summary)

Ov16 antibody tests are used for onchocerciasis surveillance. Fitting a transmission model to age-stratified seroprevalence data identified patent infection as the most likely parasite stage leading to seropositivity, indicating ongoing transmission.

## Introduction

Human onchocerciasis, caused by the filarial nematode *Onchocerca volvulus*, and transmitted among humans by the bites of female *Simulium* blackflies, is targeted by the World Health Organization (WHO) for verification of elimination of transmission in 12 endemic countries by 2030^1^. Currently, 28 countries in sub-Saharan Africa (SSA) remain endemic^2^. Mass drug administration (MDA) of ivermectin is the principal intervention strategy employed to achieve the WHO goal^1^. MDA has been used since the late 1980s in the Onchocerciasis Control Programme in West Africa (OCP, 1974-2002), and since the late 1990s in the African Programme for Onchocerciasis Control (APOC, 1995-2015)^3^. After the closure of APOC, ivermectin MDA has continued to be implemented by national programmes with technical support by the WHO’s Expanded Special Project for Elimination of Neglected Tropical Diseases (ESPEN), established in 2016^3^.

Serological assays that detect immunoglobulin G subclass 4 (IgG4) antibodies against the (recombinant) *O. volvulus* Ov16 antigen (anti-Ov16 IgG4) are central to programmatic decision-making at both poles of the elimination pathway. On the elimination end, anti-Ov16 seroprevalence in children is used (together with infectivity prevalence in blackflies) to inform stop-MDA decisions, post-treatment surveillance (PTS) and confirm elimination in post-elimination surveillance (PES)^4^. To start-MDA in previously unidentified low-transmission foci, anti-Ov16 seroprevalence in adults is used following the procedures for onchocerciasis elimination mapping (OEM)^5^. Several serological diagnostics are recommended for these purposes, including enzyme-linked immunosorbent assays (Ov16 ELISAs) and rapid diagnostic tests (Ov16 RDTs)^4,5^. These tests detect both current and past infection^6^ and are less invasive than traditional skin-snip microscopy, which is used to detect (and enumerate) dermal *O. volvulus* microfilariae^7,8^.

The WHO recommends that MDA be initiated in communities where anti-Ov16 seroprevalence is ≥ 2% in adults aged ≥20 years, providing cut-off values for Ov16 RDT using dry blood spot (DBS) eluates, the Onchocerciasis Elimination Program for the Americas (OEPA) ELISA, or the alkaline phosphatase (AP) ELISA, according to their diagnostic performance^5^. It is considered that MDA can be safely stopped when seroprevalence—measured by Ov16 ELISA—is <0.1% (at the upper 95% confidence limit) in children aged <10 years (in addition to an infectivity prevalence <0.05% in blackflies)^4^. While the former recommendation may lead to initiating MDA in populations previously considered non-endemic/hypoendemic, the latter is statistically and operationally difficult to achieve without near-perfect serological specificity^9–11^. These challenges have motivated epidemiological^12^ and transmission modelling studies^13–15^ to scrutinise the robustness and appropriateness of these thresholds, with the goal of informing programmatic decision-making with the best available evidence.

Modelling work on serological thresholds is highly sensitive to underlying assumptions on age- and sex-dependent exposure patterns and population processes regulating *O. volvulus* abundance^15^ and—critically—the parasite stage that stimulates the production of anti-Ov16 IgG4 antibodies^13^ as well as the duration of antibody persistence after infection clearance (seroreversion)^16^. For example, a study using the ONCHOSIM transmission model, indicated that an anti-Ov16 IgG4 seroprevalence <2% in children aged 5-14 years may reflect unstable or interrupted transmission^14^. However, more recent modelling using EPIONCHO-IBM has shown that unstable low prevalence may persist for decades, challenging the validity of fixed serological thresholds for stopping MDA^17^. Hence, there is a critical need to better understand the immuno-dynamics of seroconversion and seroreversion to interpret reliably serological data for programmatic decision-making.

Early work in humans and chimpanzees found that total anti-Ov16 immunoglobulin G (IgG) seroconversion occurred during pre-patent infection, before detection of microfilariae in the skin^18^. A subsequent study in non-human primates found that detection of anti-Ov16 IgG4 corresponded with the detection of microfilariae, indicative of patent infection, with some evidence of seroreversion following parasite clearance^19^. Immuno-epidemiological studies have yielded similarly inconclusive results, with evidence showing persistent IgG4 responses in post-patent individuals and variable antigen recognition patterns across infection states^20^. Hence, the *O. volvulus* parasite stage(s) predominantly involved in IgG4 antibody seroconversion remain(s) incompletely understood. This has hindered the role that modelling of anti-Ov16 IgG4 serological dynamics can play to support decision-making on serological thresholds for onchocerciasis control and elimination.

Here, we test different hypotheses on the *O. volvulus* parasite stage(s) that result(s) in anti-Ov16 IgG4 antibody seroconversion—with and without seroreversion (i.e., loss of detectable anti-Ov16 IgG4)—by fitting the individual-based EPIONCHO-IBM stochastic transmission model^21^ to seroprevalence data from a treatment-naïve setting in Gabon^22^. We then validate the parameterised model by comparing predictions (following simulated interventions) with empirical seroprevalence data collected after 39 years of onchocerciasis control in Togo^23^. Finally, we discuss how our results will strengthen the use of modelling to inform serological thresholds regarding start- and stop-MDA decisions, as well as PTS and PES for elimination of onchocerciasis transmission.

## Results

### Model calibration using microfilarial prevalence data

**Fig. 1:**
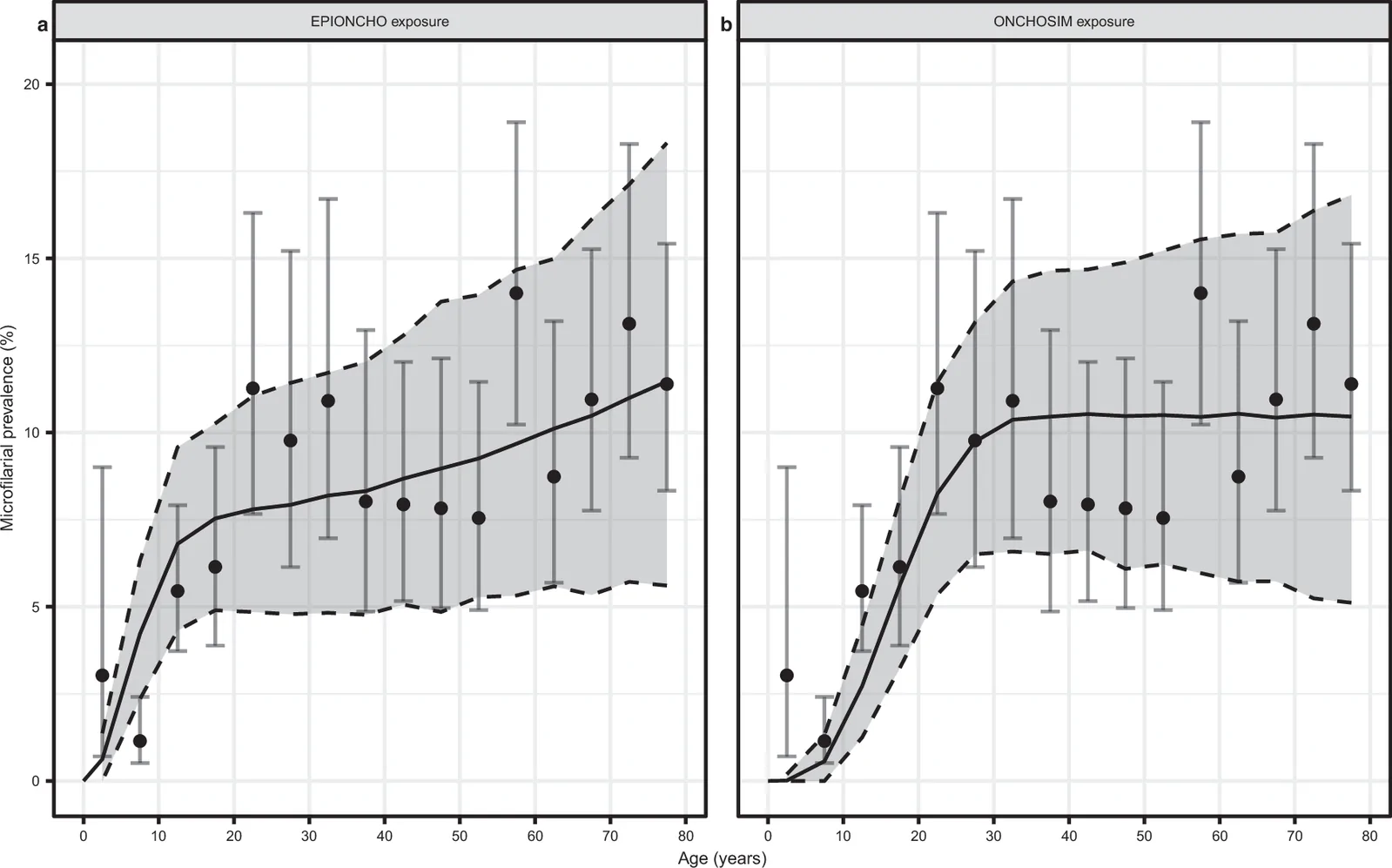
Observed and fitted microfilarial age-prevalence profiles for Gabon. The black solid lines represent the modelled mean microfilarial prevalence using EPIONCHO-IBM calibrated with different age- and sex-dependent exposure to blackfly bites. **a** Using EPIONCHO exposure, an annual biting rate (ABR) of 76 bites/person/year and an individual exposure heterogeneity parameter of *k*_*E*_ = 0.2. **b** Using ONCHOSIM exposure, an ABR of 70 bites/person/year and an individual exposure heterogeneity parameter of *k*_*E*_ = 0.2. The grey shaded areas are the 95% uncertainty intervals (2.5th to 97.5th percentiles of the simulated mean) from 1500 stochastic repeats. The black circles are the observed microfilarial prevalence estimates^22^ for 4257 individuals from 58 communities binned into 5-year age groups; the error bars are the associated 95% Wilson score confidence intervals. EPIONCHO and ONCHOSIM exposure functions are illustrated in Supplementary Fig. 1. Source data originate from Atsame et al.^22^; the code to reproduce the figure with model output is in Ramani et al.^55^.

We calibrated EPIONCHO-IBM^21^ using pre-intervention microfilarial age-prevalence data collected from 4257 participants living in 58 hypoendemic, treatment-naïve communities in Gabon^22^ by estimating the annual biting rate (ABR, no. bites/person/year) of *Simulium damnosum sensu lato* (s.l.)^21,24^ and the degree of inter-individual exposure heterogeneity, defined by parameter *k*_*E*_^21^. To account for potential bias introduced by the assumed age- and sex-dependent exposure function of the model, the calibration and hypothesis testing was replicated using the age- and sex-dependent exposure functions for both EPIONCHO-IBM^25^ (referred to as EPIONCHO exposure) and ONCHOSIM (referred to as ONCHOSIM exposure; see Supplementary Information Fig. 1^13^). The prior ranges and best-fit ABRs for *k*_*E*_ = 0.2 and *k*_*E*_ = 0.3 are shown in Supplementary Table 1 and Supplementary Fig. 2, and the observed versus fitted microfilarial age-prevalence profiles are shown in Fig. 1. Using the best-fit ABR of 76 (EPIONCHO exposure) and 70 (ONCHOSIM exposure) bites/person/year with *k*_*E*_ = 0.2, the projected microfilarial prevalence in individuals aged ≥5 years was 7.99% (95% uncertainty interval, 2.5^th^ to 97.5^th^ percentile of the simulated mean, 95% UI = 6.33%‒9.52%) with EPIONCHO exposure, and 7.65% (95% UI = 6.16%‒9.02%) with ONCHOSIM exposure, closely matching the observed prevalence of 7.90% (95% Wilson score confidence interval, 95% WCI = 7.13%‒8.75%).

We also calibrated EPIONCHO-IBM to pre-intervention microfilarial prevalence estimates from four prefectures (administrative level 2) in the Savanes and Kara regions of Togo, where vector control had been implemented (during the OCP and subsequent Special Intervention Zone (SIZ) anti-vectorial operations) for 16 and 30 years, respectively, and ivermectin MDA since 1991^23,26^. The range of best-fit ABRs for each prefecture, assuming *k*_*E*_ = 0.3^26^ is shown in Supplementary Table 2, along with the respective distributions in Supplementary Fig. 3. Supplementary Methods 1 provides further detail regarding model calibration. Supplementary Methods 2 describes the specific interventions modelled for Togo.

### Identifying the immunological stimulus for seroconversion

**Fig. 2:**
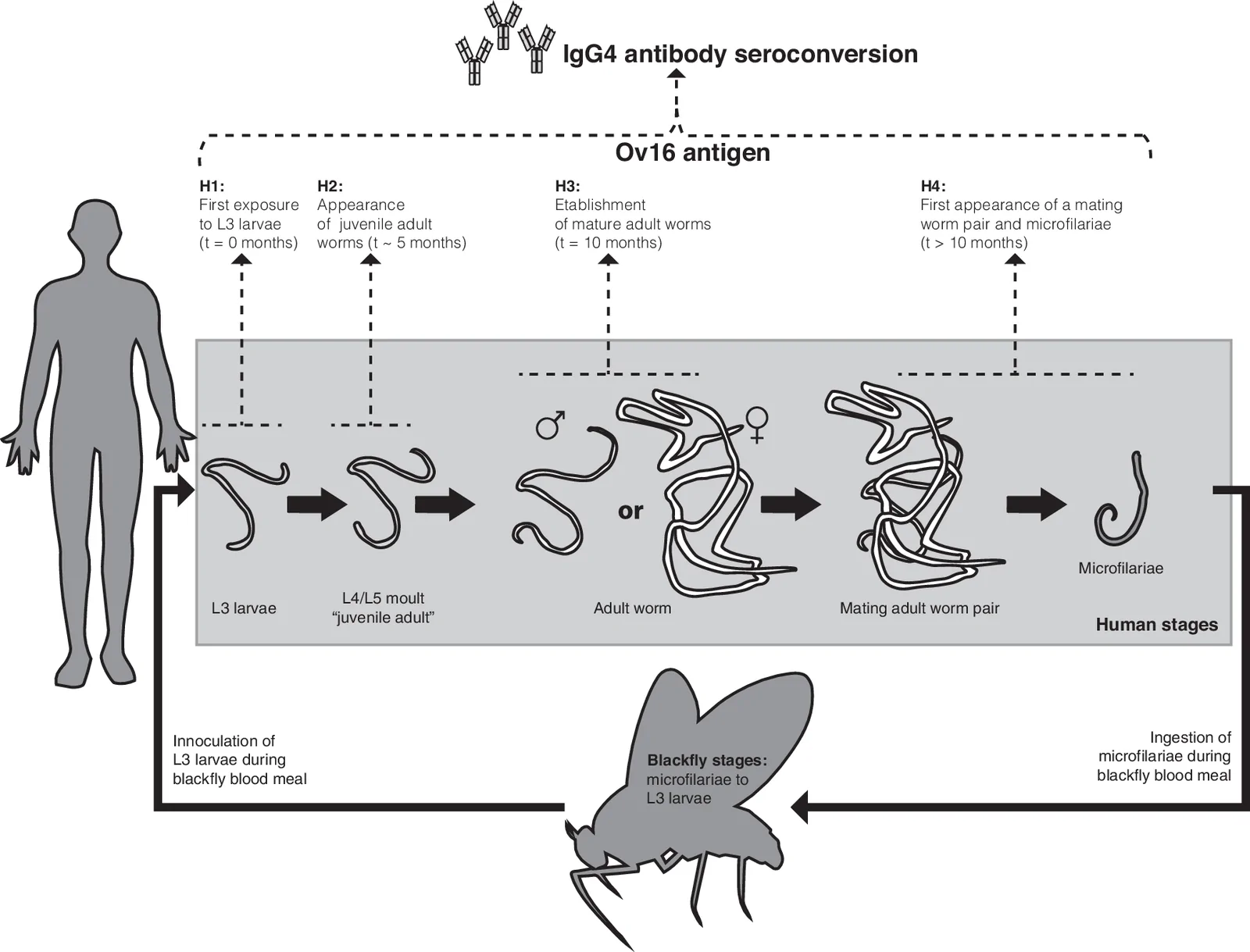
Lifecycle of *Onchocerca volvulus* showing seroconversion hypotheses incorporated into the onchocerciasis transmission model, EPIONCHO-IBM. The parasite stages resulting in the production of anti-Ov16 IgG4 antibodies are: L3 larvae (hypothesis H1); L5 (juvenile) worms following the L4-L5 moult (hypothesis H2); any mature adult *O. volvulus* (hypothesis H3), or the production of microfilariae by a mating worm pair (hypothesis H4).

Using the calibrated model, we evaluated the goodness-of-fit of model-predicted versus observed age-seroprevalence profiles (using weighted mean squared errors, MSEs) assuming various hypotheses regarding the *O. volvulus* parasite stage(s) that elicit(s) anti-Ov16 IgG4 seroconversion (Fig. 2, Table 1), integrating uncertainty in the diagnostic performance characteristics (sensitivity and specificity) of the SD BIOLINE Ov16 RDT using whole blood^22^ by sampling from independent beta distributions informed by previously published estimates (Supplementary Table 3, Supplementary Fig. 4, Supplementary Methods 3). Comparisons were summarised using 95% Monte Carlo confidence intervals (95% MCCIs integrating stochastic uncertainty) associated with the weighted MSEs.

**Table 1:** Seroconversion and seroreversion hypotheses tested

Seroconversion hypothesis	No seroreversion^a^ (lifelong immunological memory) (i)	Immediate seroreversion^b^ (no immunological memory) (ii)	Finite seroreversion^c^ (finite immunological memory) (iii)
Exposure to L3 larvae (H1)	H1i	H1ii	H1iii
During the L4-L5 moult (H2)	H2i	H2ii	H2iii
Presence of any adult worm (of either sex) (H3)	H3i	H3ii	H3iii
Presence of microfilariae (from a mating worm pair), not necessarily detectable by skin-snip microscopy (H4)	H4i	H4ii	H4iii

^a^No seroreversion assumes that anti-Ov16 IgG4 antibodies persist for life (i.e., lifelong immunological memory).

^b^Immediate seroreversion assumes that anti-Ov16 IgG4 antibodies persist only while the parasite stage stimulating seroconversion is present (i.e., no immunological memory).

^c^Finite seroreversion assumes that anti-Ov16 IgG4 antibodies persist until all parasite stages (L3-L5, adult worms and microfilariae) are cleared, irrespective of the seroconversion stimulus (i.e., finite immunological memory).

**Fig. 3:**
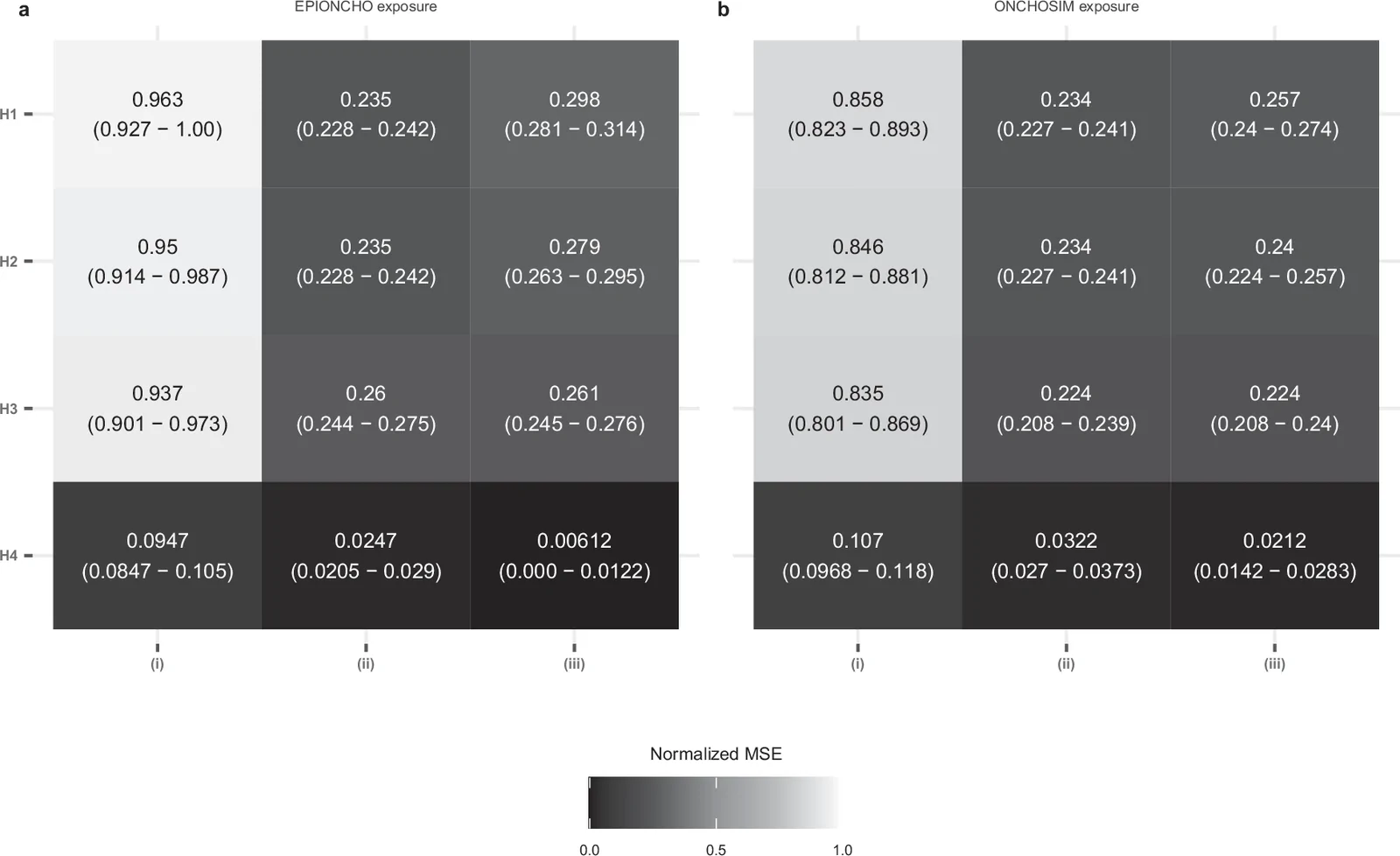
Quantitative assessment of goodness-of-fit of anti-Ov16 IgG4 seroconversion-seroreversion hypotheses under different assumptions on age- and sex-dependent exposure. Each tile shows the normalised, weighted mean squared errors (MSEs) and 95% Monte Carlo confidence intervals (95% MCCIs), calculated by comparing EPIONCHO-IBM-predicted age-seroprevalence profiles with observed anti-Ov16 IgG4 seroprevalence estimates from 4257 individuals living in 58 ivermectin-naïve, hypoendemic communities in Gabon^22^. **a** Model simulations using EPIONCHO exposure (age- and sex-dependent exposure to blackfly bites)^25^. **b** Model simulations using ONCHOSIM exposure^13^. For each seroconversion (H1-H4) and seroreversion (i-iii) hypothesis (Table 1), MSEs were calculated for each of 1500 stochastic repeats, each adjusted for 1500 combinations of sensitivity and specificity sampled from independent beta distributions (with mean sensitivity of 48.2% and mean specificity of 96.9%; see Supplementary Methods 3), weighted by the proportion of observations within a given age-group. These MSEs were then normalised to the interval [0,1] relative to the lowest (best) unnormalised weighted MSE across hypotheses (i.e., tiles show MSE relative to the best fit). Unnormalised values and 95% MCCIs are shown in Supplementary Tables 4–5 and point estimates of sensitivity/specificity in Supplementary Tables 6–7. Darker tiles correspond to lower (better) normalised MSEs. With both exposure functions, seroconversion initiated by near-patent/patent infection (H4) provided the best fit to the data. With EPIONCHO exposure, hypothesis H4iii (finite seroreversion) provided the best quantitative fit to the data, while with ONCHOSIM exposure, H4ii (immediate seroreversion) and H4iii (finite seroreversion) were statistically indistinguishable based on overlapping 95% MCCIs.

The hypothesis that seroconversion is elicited by near-patent/patent infection (H4, Table 1) provided a better quantitative fit to the data (lower mean squared error, MSE), irrespective of the assumed exposure function (EPIONCHO exposure vs ONCHOSIM exposure; Fig. 3). Additionally, we were unable to distinguish between the immediate seroreversion hypothesis (i.e., no immunological memory, H4ii in Table 1) and the finite seroreversion hypothesis (i.e., finite immunological memory, H4iii in Table 1). While the 95% MCCIs of the MSEs associated with H4ii and H4iii do not overlap for EPIONCHO exposure^25^, they do for ONCHOSIM exposure^13^ (Fig. 3; unnormalised MSEs and associated 95% MCCIs are shown in Supplementary Table 4 for EPIONCHO exposure and Supplementary Table 5 for ONCHOSIM exposure). The MSEs for point estimates of the sensitivity and specificity for EPIONCHO and ONCHOSIM exposure are shown, respectively, in Supplementary Tables 6 and 7.

**Fig. 4:**
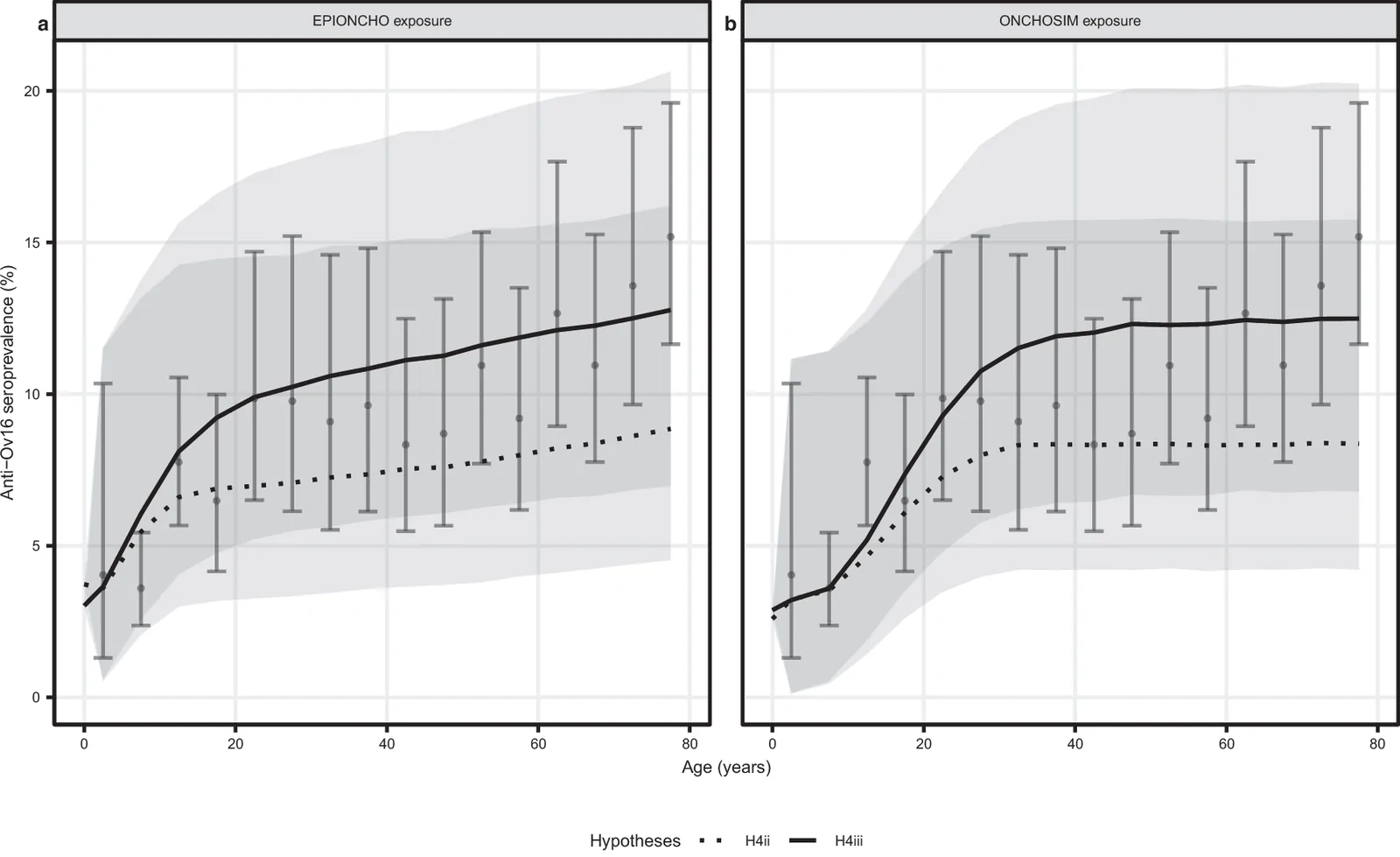
Observed and EPIONCHO-IBM-predicted anti-Ov16 IgG4 age-seroprevalence profiles for 4257 individuals living in 58 ivermectin-naïve communities in Gabon. The panels show the mean predicted age-seroprevalence using EPIONCHO-IBM calibrated to observed microfilarial prevalence estimates (Fig. 1)^22^ assuming different age- and sex-specific exposure to blackfly bites. **a** Assuming EPIONCHO exposure^25^, an annual biting rate (ABR) of 76 bites/person/year, and an individual exposure heterogeneity parameter of *k*_*E*_ = 0.2. **b** Assuming ONCHOSIM exposure^13^, an ABR or 70 bites/person/year, and an individual exposure heterogeneity parameter of *k*_*E*_ = 0.2. Predictions assume that anti-Ov16 IgG4 seroconversion is elicited by near-patent/patent infection (hypothesis H4) with either immediate seroreversion (H4ii, dotted line) or finite seroreversion (H4iii, solid line), and are adjusted for the diagnostic performance of the SD BIOLINE Ov16 rapid diagnostic test using whole blood (sampled from independent beta distributions with mean sensitivity of 48.2% and mean specificity of 96.9%; see Supplementary Methods 3). Grey circles show observed seroprevalence estimates binned into 5-year age groups in 4257 individuals; error bars represent 95% Wilson score confidence intervals. Shaded areas depict 95% uncertainty intervals (2.5th to 97.5th percentiles of the simulated mean), based on 1500 stochastic repeats, each adjusted by 1500 samples from the diagnostic performance distributions. Source data originate from Atsame et al.^22^; the code to reproduce the figure with model output is in Ramani et al.^55^.

The observed and predicted age-seroprevalence profiles for these best fitting (indistinguishable) hypotheses (H4ii and H4iii) are shown in Fig. 4. Both hypotheses were advanced for validation against the Togo seroprevalence data^23^, collected after 39 years of onchocerciasis control. Supplementary Fig. 5 shows the observed and predicted age-seroprevalence profiles for all seroconversion-seroreversion hypotheses (H1i-H4iii, Table 1) using diagnostic sensitivity/specificity sampled from independent beta distributions (i.e., integrating diagnostic performance uncertainty). Supplementary Fig. 6–8 show the corresponding age-seroprevalence profiles for point estimates of sensitivity/specificity (i.e., point value sensitivity analysis).

### Validating seroprevalence predictions under intervention

We used EPIONCHO-IBM, calibrated to pre-intervention microfilarial prevalence estimates from Togo^26^, to predict anti-Ov16 IgG4 seroprevalence profiles using the best-fitting seroconversion hypothesis (H4: near-patent/patent infection) and the two indistinguishable seroreversion hypotheses (H4ii: immediate seroreversion; H4iii: finite seroreversion) identified from the Gabon data.

Model simulations considered 39 years of intervention in four prefectures (administrative level 2; Ôti/Kpendjal, Kéran, and Bassar), including vector control from 1976-2007 (ending in 1993 in Ôti/Kpendjal)^26^ and MDA from 1991 to 2015^23,26^. Intervention effectiveness was modelled in a prefecture-specific manner, as defined by Amaral et al.^26^ based on vector control efficacy (proportional reduction in ABR for the duration of implementation), MDA coverage and systematic non-adherence (the proportion of individuals never participating in MDA). As the model was calibrated using work from Amaral et al.^26^, which used EPIONCHO-IBM for its analysis, only EPIONCHO exposure^25^ (Supplementary Fig. 1a) was used for the validation. Supplementary Table 8 provides parameter values and categorizations of intervention effectiveness. A comparison of the model-predicted versus observed microfilarial prevalence estimates in 2015 is given in Supplementary Table 9.

**Fig. 5:**
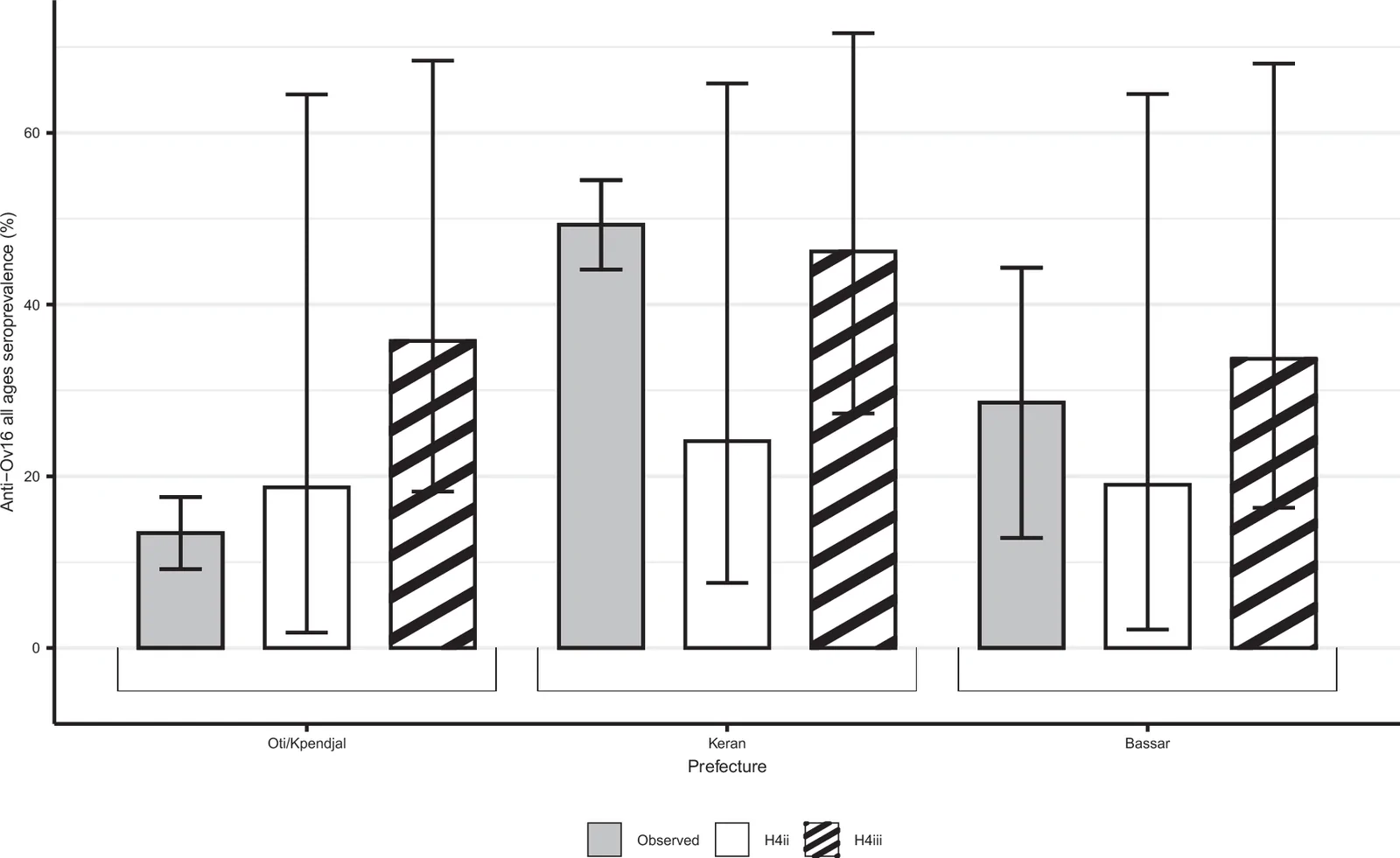
Observed and predicted anti-Ov16 IgG4 seroprevalence among 644 individuals (aged 5–80 years) in four prefectures of Togo (Ôti/Kpendjal, Kéran, and Bassar) after 39 years of anti-vectorial and anti-parasitic intervention, under assumptions of immediate or finite seroreversion. Grey bars show observed seroprevalence estimates in 2015 and associated 95% Wilson score confidence intervals^23^. White and hatched bars represent model-derived, mean predicted (5–80 years) seroprevalence generated by EPIONCHO-IBM, calibrated to model-derived pre-intervention microfilarial prevalence estimates (with age- and sex-dependent EPIONCHO exposure to blackfly bites)^25^, and inter-individual exposure heterogeneity parameter, *k*_*E*_ = 0.3)^26^ using the best-fit seroconversion hypothesis (anti-Ov16 IgG4 antibody production elicited by near-patent/patent infection) identified from the Gabon dataset^22^. Model predictions are adjusted for the uncertain diagnostic performance of the horseradish peroxidase, HRP Ov16 ELISA used in Togo^23^ by sampling from independent beta distributions (with mean sensitivity of 76.4% and mean specificity of 83.2%; see Supplementary Methods 3). White bars indicate model predictions assuming immediate seroreversion (hypothesis H4ii); hatched bars indicate model predictions assuming finite seroreversion (hypothesis H4iii). Black error bars for model predictions denote 95% uncertainty intervals (2.5th to 97.5th percentiles of simulated means) based on 1500 stochastic repeats per prefecture (i.e., 4500 simulations in total), each adjusted by 1500 samples from the diagnostic performance (sensitivity/specificity) distributions. Historical interventions included vector control (aerial larviciding of *Simulium damnosum* *sensu lato* (s.l.) breeding sites: 1976–1993 in Ôti/Kpendjal; 1976–2007 in Kéran and Bassar) and ivermectin mass drug administration (1991–2015)^26^. Intervention effectiveness was modelled as minimal in Bassar, enhanced in Kéran, and a mix of minimal, reference, and enhanced in Ôti/Kpendjal. See Supplementary Table 8 for parameters defining intervention effectiveness categories. Source data originate from Komlan et al.^23^; the code to reproduce the figure with model output is in Ramani et al.^55^.

The predictive performance of the prefecture-specific EPIONCHO-IBM seroprevalence projections, under the two seroreversion hypotheses and for diagnostic performances (sensitivity/specificity) of the horseradish peroxidase (HRP) Ov16 ELISA used in Togo^23^ (Supplementary Table 10)—sampled from independent beta distributions (Supplementary Fig. 9; Supplementary Methods 3)—are shown qualitatively in Fig. 5 and quantitatively (using MSEs and 95% MCCIs) in Supplementary Table 11. Predictive performance using point estimates of sensitivity and specificity are shown in Supplementary Table 12. Model performance varied by prefecture, with no single seroreversion hypothesis consistently providing the best fit. In both Kéran and Bassar, the best predictive performance was obtained under the finite seroreversion hypothesis (H4iii). By contrast, in Ôti/Kpendjal, the immediate seroreversion hypothesis (H4ii) yielded the best fit.

**Fig. 6:**
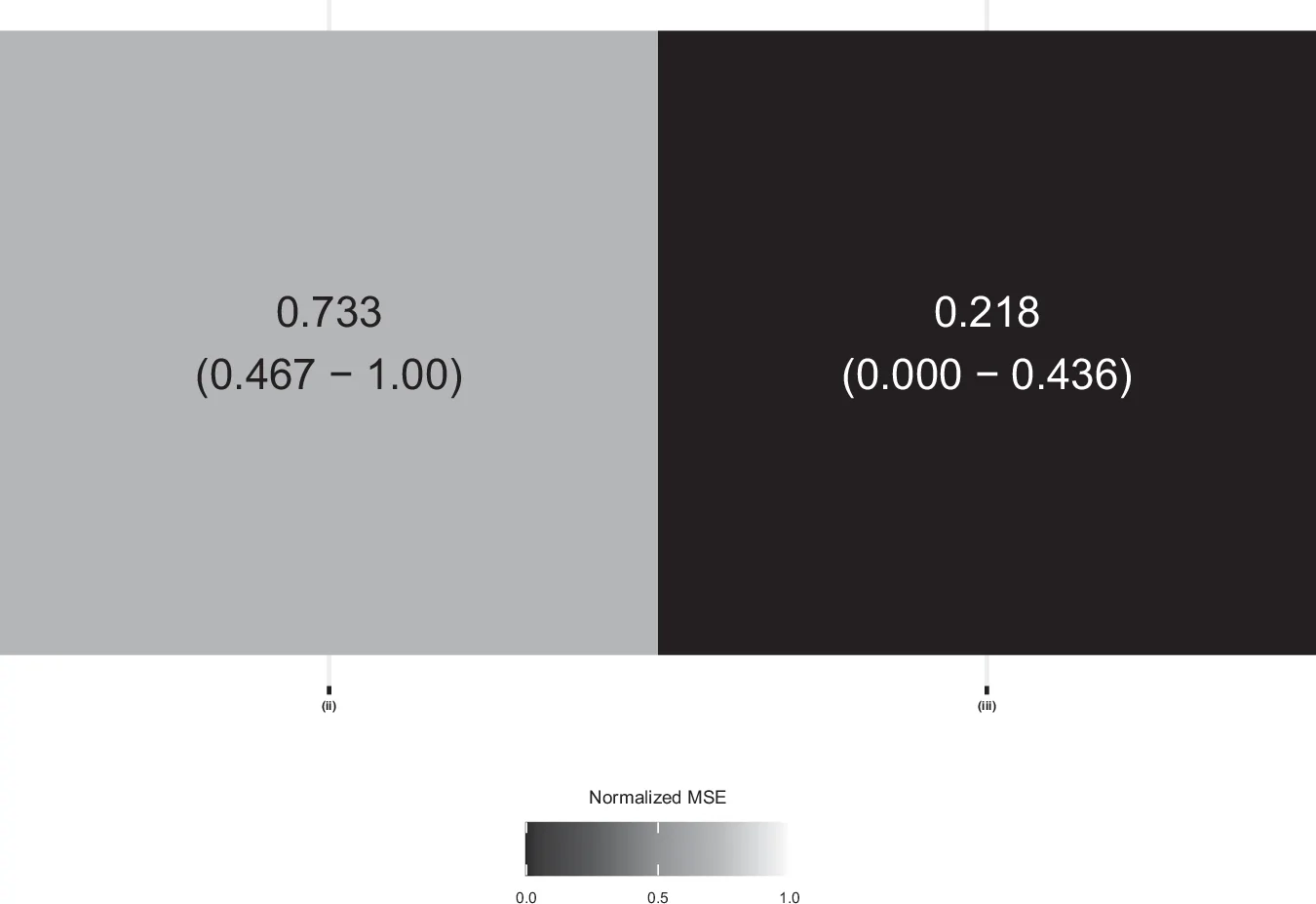
Quantitative assessment of EPIONCHO-IBM predictive performance under two seroreversion hypotheses. Each tile shows the normalised, weighted mean squared error (MSE) and 95% Monte Carlo confidence interval (95% MCCI), calculated by comparing EPIONCHO-IBM-predicted 5-80-year-old anti-Ov16 IgG4 seroprevalence with observed anti-Ov16 IgG4 5-80-year-old seroprevalence estimates from 576 individuals from four prefectures in Togo after 39 years of anti-vectorial and anti-parasitic intervention^23^. Model simulations use age- and sex-dependent EPIONCHO exposure to blackfly bites)^25^. For each seroreversion (ii, iii) hypothesis (Table 1), prefecture-specific MSEs were calculated from 1500 stochastic repeats, for each of 1500 combinations of sensitivity and specificity sampled from independent beta distributions (with a mean sensitivity of 76.4% and mean specificity of 83.2%, see Supplementary Methods 3). The single prefecture-combined MSE for a hypothesis was calculated as the arithmetic mean of the 3 prefecture-specific MSEs, weighted by the percentage contribution of each population to the observed anti-Ov16 seroprevalence data^23^. These MSEs were then normalised to the interval [0,1] relative to the lowest (best) unnormalised weighted MSE across hypotheses (i.e., tiles show MSE relative to the best fit; unnormalised values and 95% MCCIs are shown as prefecture-specific estimates in Supplementary Table 11 and prefecture-combined estimates in Supplementary Table 13). Both hypotheses assume anti-Ov16 IgG4 seroconversion is initiated by near-patent/patent infection (Table 1). Darker tiles indicate lower (better) normalised MSEs. The best quantitative fit to the data was achieved under hypothesis H4iii, seroconversion elicited by near-patent/patent infection with finite seroreversion.

Aggregated predictive performance across all prefectures—summarised using normalised MSEs—is shown in Fig. 6, with corresponding unnormalised values and 95% MCCIs in Supplementary Table 13 (with aggregated predictive performance for point estimates of sensitivity and specificity shown in Supplementary Table 14). Overall, the finite seroreversion hypothesis (H4iii) provided the lowest MSE. However, it is important to note that this result belies the comparatively poor fit in Ôti/Kpendjal with the finite seroreversion hypothesis (H4iii, see Supplementary Table 11). Hence, although H4iii yielded the best overall performance, it cannot be considered definitively superior across all settings.

**Fig. 7:**
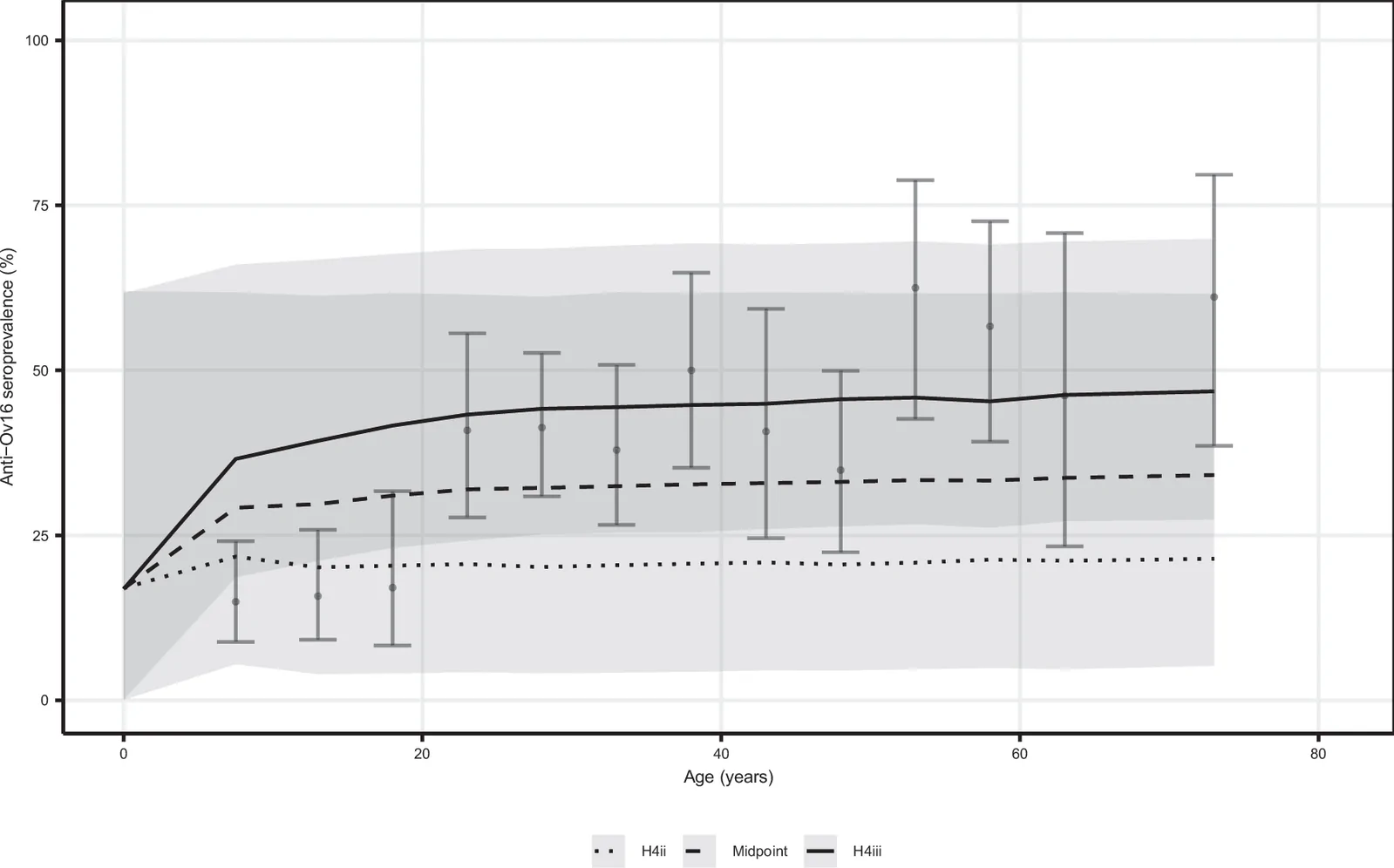
Observed and predicted anti-Ov16 IgG4 age-seroprevalence profiles for 576 individuals (aged 5-80 years) in four prefectures of Togo (aggregated) after 39 years of anti-vectorial and anti-parasitic intervention. The solid, dashed, and dotted lines represent mean predicted age-seroprevalence profiles generated by EPIONCHO-IBM, calibrated to inferred pre-intervention microfilarial prevalence estimates^26^ (with EPIONCHO exposure^25^ and inter-individual exposure heterogeneity parameter, *k*_*E*_ = 0.3) and using the best-fitting H4 seroconversion hypothesis (anti-Ov16 IgG4 elicited by near-patent/patent infection) identified from the Gabon seroprevalence data^22^. Predictions are adjusted for the uncertain diagnostic performance of the horseradish peroxidase, HRP Ov16 ELISA used in Togo^23^ by sampling from an independent beta distribution (with mean sensitivity of 76.4% and mean specificity of 83.2%; see Supplementary Methods 3). The dotted line indicates predictions assuming immediate seroreversion (hypothesis H4ii), the solid line corresponds to predictions assuming finite seroreversion (hypothesis H4iii), and the dashed line shows the mean of these two hypotheses for visualisation purposes. Shaded areas depict 95% uncertainty intervals (2.5th to 97.5th percentiles of simulated means) based on 1500 stochastic repeats per prefecture (i.e., 4500 simulations in total), each adjusted by 1500 samples from the diagnostic performance distributions. The prefecture-combined age-seroprevalence for a hypothesis was calculated as the arithmetic mean of the 3 prefectures, weighted by the percentage contribution of each prefecture to the observed anti-Ov16 data^23^. Historical interventions included vector control (aerial larviciding of *Simulium damnosum* *sensu lato* (s.l.) breeding sites: 1976–1993 in Ôti/Kpendjal; 1976–2007 in Kéran and Bassar) and ivermectin mass drug administration (1991–2015)^26^. Intervention effectiveness was modelled as minimal in Bassar, enhanced in Kéran, and a mix of minimal, reference, and enhanced in Ôti/Kpendjal. See Supplementary Table 8 for parameters defining intervention effectiveness categories. Source data originate from Komlan et al.^23^; the code to reproduce the figure with model output is in Ramani et al.^55^.

The predicted versus observed age-seroprevalence profiles aggregated across all prefectures (prefecture-specific age-seroprevalence estimates were not available^23^) for H4ii and H4iii are shown in Fig. 7. Model predictions using H4iii tended to overestimate seroprevalence in younger age groups ( ≤ 20 years), while those using H4ii did not, although the observed seroprevalence estimates for both hypotheses fall within the 95% UIs of the model predictions. In older age groups ( > 20 years), predictions under H4ii and H4iii tended to straddle the observed data. For visualisation purposes only, the midpoint of the two hypotheses is also shown in Fig. 7.

## Discussion

Understanding the dynamics of anti-Ov16 IgG4 antibody responses is essential to support onchocerciasis elimination efforts, particularly as programmes have moved from parasitological to serological surveillance for decision-making. Since 2016, the WHO has recommended the use of anti-Ov16 IgG4 serology (together with blackfly vector xenomonitoring) to support decisions on stopping ivermectin MDA and proceeding with PTS and PES^4^. However, until now, modelling of seroprevalence dynamics has lagged behind modelling of microfilarial prevalence^26–28^, owing to critical uncertainties in the immunological stimulus for seroconversion and the persistence of IgG4 antibody responses against the Ov16 antigen^13–15^.

Our findings indicate that the production of anti-Ov16 IgG4 antibodies is likely elicited by parasite lifecycle stages associated with near-patent/patent infection, around the time of microfilarial production by mating worm pairs. Using this parameterisation, EPIONCHO-IBM accurately reproduced age-seroprevalence profiles in two contrasting epidemiological and programmatic settings: a hypoendemic untreated setting in Gabon^22^, and a highly endemic setting with nearly four decades of interventions in Togo^23^. This represents a major advance in the use of transmission models to inform serological thresholds for key public health decisions, such as initiating or stopping MDA, and informing the elimination of onchocerciasis transmission during the PTS and PES periods. Specifically, by embedding a biologically-motivated seroconversion–seroreversion process within a validated transmission model, our framework provides a quantitative mapping from underlying transmission status and intervention history to the expected age-specific distribution of Ov16 seroprevalence (under imperfect test performance), which is essential to evaluate and refine operational start-/stop-MDA and PTS/PES serological thresholds.

Experimental^18,19^ and clinico-immunological^20,29^ evidence on the stimulus that leads to the production of anti-Ov16 IgG4 antibodies has remained inconclusive since the first development of anti-Ov16 serology in the late 1980s (initially using total IgG)^18^ and the precursors of the current Ov16 RDTs^30^. Our finding that IgG4 seroconversion is most likely stimulated by parasite stages surrounding the timing of patent infection aligns with the findings of Cama et al.^19^ and is consistent with IgG4 antibodies being produced later than, for instance, immunoglobulin E (IgE)^29^. Moreover, microfilariae (and female worms) have been associated with transforming growth factor beta (TGF-β) expression (among other cytokines produced by regulatory T cells)^31,32^, which in turn is implicated in the induction of IgG4 antibodies^31^. Previous modelling studies have assumed that seroconversion is initiated by pre-patent parasite stages^13–15^, which we demonstrate yields substantially different and less accurate age-seroprevalence profiles.

Importantly, our modelled seroconversion mechanism does not require microfilariae to be detectable by skin-snip microscopy, recognising the limitations of this diagnostic in low-intensity infections^5,10,33^. This helps explain observations of seropositive but skin-snip negative children^18,29^. Moreover, our finding that near-patent/patent infection is the predominant stimulus for IgG4 seroconversion is consistent with other studies suggesting that anti-Ov16 IgG4 seropositivity is a good marker of active infection in children under 11 years of age^34^. Notably, this conclusion held across a range of assumed sensitivities and specificities of the Ov16 RDT protocol used in Gabon^22^, which we varied to reflect the range of published estimates^22,35–37^ (Supplementary Methods 3).

Despite these advances, an important remaining source of uncertainty is the persistence of anti-Ov16 IgG4 antibodies (i.e., the duration of immunological memory). In our analysis of the Gabon data, we were unable to distinguish between immediate seroreversion (i.e., seroreversion occurring after the loss of all mating worm pairs) and finite seroreversion (i.e., occurring after clearance of all parasite stages). This is not unexpected as seroreversion and diagnostic sensitivity are confounded; higher sensitivity can compensate, to some extent, for declining antibody responses. This can be seen in Supplementary Figs. 6–8, and Supplementary Tables 6–7, where, when using point diagnostic performance adjustments, simulations assuming no seroreversion with poorer diagnostic performance provide the best fit to the Gabon data, while simulations using instant or finite seroreversion provided the best fit as diagnostic performance increased.

Relatedly, an Ov16-negative result in an infected individual may reflect several processes, including variability in the magnitude and timing of the Ov16-specific IgG4 response, antigenic coverage of the assay (i.e., individuals whose humoral response is dominated by reactivity to other parasite antigens), and technical performance of the ELISA or RDT. In a paediatric study from Sierra Leone^29^, only 75% of skin-snip positive children had detectable IgG to Ov16, and 10/53 microfilaria-positive children had no detectable IgG to any of four recombinant antigens (Ov11, Ov27, Ov29 or Ov16) despite low–moderate responses to crude adult worm antigen; the authors concluded that additional epitopes may be needed and noted that longitudinal follow-up would be required to determine the significance of these antibody-negative infections. Likewise, contemporary evaluations of Ov16 assays in endemic settings consistently report sensitivities below 100% among parasitologically-confirmed infections^19,30,34,36^. These observations are sometimes interpreted as evidence of stable non-responder phenotypes; however, direct evidence for persistent individual-level failure to mount an Ov16-specific IgG4 response is limited because most data are cross-sectional and antibody responses can both lag behind infection and wane after clearance^16,19^. In our modelling, we interpret sensitivity as an effective probability that a truly infected individual is observed as being Ov16-positive at a given survey, subsuming these biological and technical sources of discordance rather than attributing them to fixed host phenotypes.

The seroprevalence data from Togo are potentially more informative on seroreversion dynamics since—after decades of intervention—transmission may be close to interruption in some of the surveyed villages^26^. A study in Guatemala estimated a mean IgG4 antibody decay rate of 0.20/year and a half-life of 3.3 years, with 73% of previously seropositive individuals seroreverting 2-3 years after transmission was interrupted and MDA halted^16^. When aggregated across the four prefectures in Togo, our findings point to finite seroreversion, providing a better fit to the data. However, this conclusion is obfuscated by results from Ôti/Kpendjal, where assuming immediate seroreversion (H4ii) yielded considerably better predictions than with finite seroreversion (H4iii). Thus, while we are confident that near-patent/patent infection is the predominant seroconversion stimulus, our findings remain inconclusive regarding the dynamics of seroreversion.

Several factors underlie this residual uncertainty. We implemented seroreversion as a binary (on/off) process rather than as a decay in antibody titres. This simplification may lack sufficient nuance to capture accurately observed age-seroprevalence patterns. Indeed, the immediate and finite seroreversion hypotheses qualitatively straddled the observed data, suggesting that a more refined approach to modelling seroreversion may be necessary. Furthermore, it is likely that villages from the different prefectures in Togo were (in 2015) differentially close to interruption of transmission^26^, meaning that signals of seroreversion in some may have been masked by ongoing transmission in others. Notwithstanding, even with a more nuanced approach to modelling seroreversion and a village-level analysis, definitive inference may remain elusive given the substantial uncertainty around the performance of the HRP Ov16 ELISA used in Togo, for which variation in sensitivity and specificity can mimic effects of seroreversion. In addition, diagnostic performance may plausibly vary across epidemiological contexts and intervention histories (e.g., if antibody titres are lower or more heterogeneous in hypoendemic settings or after prolonged MDA), which could contribute to setting-specific effective sensitivity. We do not model titre dynamics explicitly here and therefore treat sensitivity/specificity as uncertain effective parameters rather than attempting to infer context-dependent diagnostic performance characteristics from these data.

The sensitivity and specificity of the HRP Ov16-ELISA used in Togo were previously estimated by comparing test results to skin-snip microscopy^23^. Given the low microfilarial prevalence across villages (0%-13.6% in 2015) and the reduced sensitivity of skin-snip microscopy in low-intensity infections^5,10,33^, the reported specificity of the ELISA was most likely underestimated^23^. This explains why model predictions systematically overestimated seroprevalence when using a point specificity of 70% (Supplementary Table 12)—resulting in an excess of false positives—and improved with increased specificity (or when simulating from a distribution of specificities with mean 83.2%, consistent with the range of estimates from the literature; Supplementary Fig. 9, Supplementary Methods 3)^6,34,35^. Conversely, the estimated 70% sensitivity of the HRP Ov16-ELISA—derived from using the near-100% specificity of microfilarial detection—is probably unbiased, aligning with other published estimates of sensitivity, which are in the range of 50% to 95%^6,7,23,34,35,38,39^ (details of the age ranges and comparators used for previous sensitivity and specificity estimates of the HRP Ov16-ELISA are given in Supplementary Table 10).

Modelling 39 years of intervention in Togo necessarily made use of several assumptions. Due to the lack of village-level treatment and vector control history^23^, we applied prefecture-level generalisations for MDA coverage and vector control efficacy, assuming that all villages within a prefecture shared the same history of intervention^26^. Additionally, due to limited geo-located, age-stratified data, we aggregated model predictions across prefectures. These assumptions may have contributed to our overprediction of seroprevalence in Ôti/Kpendjal with H4iii, where vector control efficacy in some villages may have approached 100%^26,40^, higher than the maximum of 90% considered here, albeit our microfilarial prevalence predictions were consistent with the observed data in all prefectures (Supplementary Table 9). As an additional robustness check, we repeated the Togo validation allowing vector-control efficacy to reach 100% in the enhanced scenario for Ôti/Kpendjal; however, the results were not substantially different and did not affect our principal conclusions (Supplementary Tables 11 and 13). Given the extended timelines of onchocerciasis programmes, such data limitations are not uncommon^26,27,41^. For example, in the absence of detailed entomological time series, it is not possible to capture potential secular changes owing to climate and/or environmental change and, consequently, only vector control was assumed to effect reductions in blackfly ABRs^26^.

It is also important to acknowledge other important assumptions embedded within EPIONCHO-IBM. These include density-dependent parasite establishment in humans, whereby the efficiency of transmission from vectors to humans *increases* with *decreasing* transmission intensity^42^, and age- and sex-specific exposure profiles^15,25^. Compared to the ONCHOSIM transmission model^41,43,44^, the default EPIONCHO exposure function within EPIONCHO-IBM reflects higher exposure in children younger than 10 years^25^, which could potentially overestimate seroprevalence in this age group (although it has successfully captured microfilarial loads in 5-10-year-old children in high-transmission settings^45^). Lont et al.^13^ (using ONCHOSIM) showed that higher exposure can be offset by lower sensitivity to yield equivalent seroprevalence in children. A salient advantage of our analysis is the use of all-age seroprevalence data from Gabon^22^, the integration of uncertainty in diagnostic performance, and the comparison of results when using age- and sex-dependent EPIONCHO and ONCHOSIM exposure functions. While some combinations of exposure, diagnostic performance, and seroreversion can partially compensate and yield similar fits for specific age groups, the all-age data and uncertainty-integrated fitting framework show that, on average across the sampled parameter ranges, the near-patent/patent seroconversion hypothesis consistently outperforms hypotheses invoking pre-patent seroconversion.

As onchocerciasis elimination programmes continue transitioning away from using skin-snip microscopy to less invasive serological surveillance, it is essential that transmission models advance in parallel to remain useful for public health decision-making. Here, we have incorporated an immunologically motivated anti-Ov16 IgG4 seroconversion-seroreversion sub-model into EPIONCHO-IBM, indicating that seroconversion seems most likely elicited by near-patent/patent *O. volvulus* infection. The model captures observed age-seroprevalence profiles both in treatment-naïve and intervention settings, with varying age- and sex-dependent exposure. This is a major step forward for onchocerciasis transmission dynamics modelling, providing a critical tool to support programmatic decisions on initiating MDA, stopping treatment safely, determining elimination, and responding to serological signals during PTS and PES.

## Methods

### Ethics statement

This study analysed, through transmission dynamics modelling, de-identified, individual-level and aggregate village-level microfilarial prevalence and anti-Ov16 IgG4 seroprevalence data collected in Gabon and Togo in 2015. Ethical approval for these studies was obtained, respectively, from the Ministry of Health and Social Affairs of the Republic of Gabon (APOC/EVE/030/15/HZ)^22^, and the Togolese Bioethics Committee for Research in Health (Comité de Bioéthique pour la Recherche en Santé; CBRS (#013/2015/CBRS/3.Septembre 2015)^23^. The study in Togo was also granted study authorization and approval by the Ministry of Health of Togo (#338/2015/MSPS/CAB/SG)^23^. For our (in silico) study, no human participants were recruited and no individual-level identifiable data were used; therefore, no institutional review board approval and informed consent were required for the present (modelling) analysis.

### EPIONCHO-IBM

**Fig. 8:**
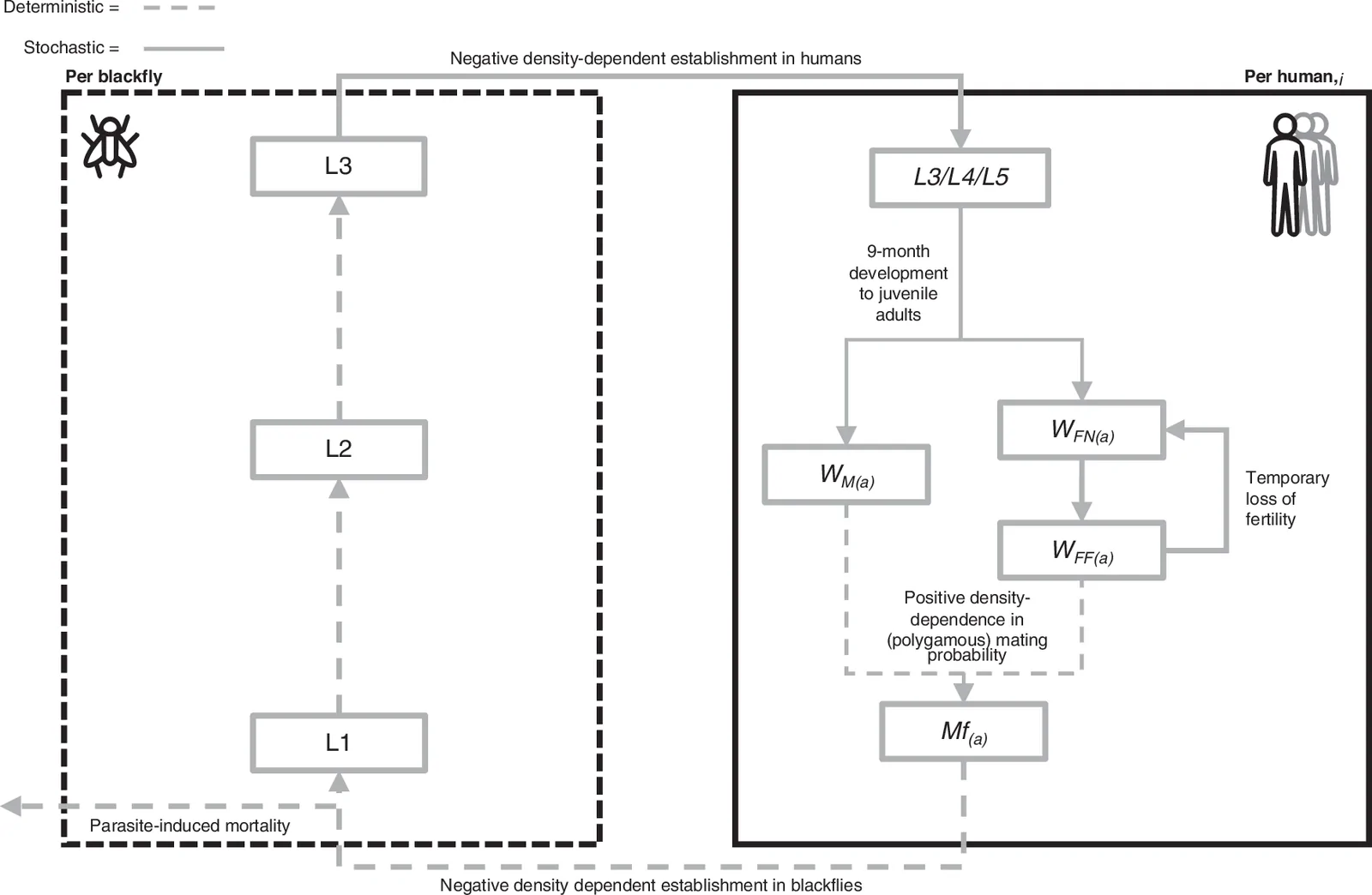
EPIONCHO-IBM model schematic. EPIONCHO-IBM^21^ is an individual-based onchocerciasis transmission model, extending a deterministic precursor, EPIONCHO^27,42^. The model considers *Onchocerca volvulus* infection dynamics in the blackfly vector (per blackfly box) and human hosts (per human box). Deterministic components are denoted by dashed lines; stochastic components by solid lines. Within an individual human host, *i*, the model tracks numbers of pre-patent *L3, L4*, and *L5 O. volvulus* stages, adult worms (*W*) and microfilariae (*Mf*). Microfilariae are the *L1* parasite stage. Numbers of adult males (*W*_M_) and females (*W*_F_) are tracked separately (assuming a 1:1 sex ratio). Females are initially non-fertile (*W*_FN_) and cycle between fertile (*W*_FF_) and non-fertile, reflecting their periodic reproduction biology^47,48^. *Mf* are produced following the mating of adult male and fertile female worms, assuming complete polygamy (i.e., a single adult male can inseminate all fertile females). The survival of adult worms and microfilariae is modelled using a Weibull distribution, such that the average lifespan of adults is 9.28 years (with 95% dead by 12.0 years) and that of microfilariae is 0.833 years (with 95% dead by 1.09 years). Parasite fecundity decreases nonlinearly with age, the fecundity rate halving by 13 years. Individuals are exposed to blackfly bites depending on their age and sex, in addition to an individual exposure heterogeneity (gamma distributed) factor randomly assigned at birth. The latter leads to most individuals having low worm burdens, and a few harbouring high worm burdens, generating mechanistically the classic overdispersed distribution that is a hallmark of helminth epidemiology. The dynamics of *L1*, *L2*, and *L3* larvae within vectors are modelled deterministically, with differential equations defining their progression. The extrinsic incubation period of the parasite within blackflies takes approximately one week under tropical conditions. The dynamics of *Mf* within humans are also modelled deterministically. Density-dependent processes are assumed to operate upon parasite establishment within humans and blackfly vectors, vector survival (affected by the number of ingested microfilariae), and the probability that a female worm is mated.

EPIONCHO-IBM is a stochastic individual-based onchocerciasis transmission model^21,46^, based on its deterministic precursors^25,27,42^. The model tracks *O. volvulus* life stages within individual human hosts—pre-patent (L3, L4, L5), adult worms and microfilariae (per milligram of skin or per skin snip)—and the mean number of L3 per blackfly vector, with the dynamics of L1, L2 and L3 larvae within the vector modelled deterministically^27^. The dynamics of microfilariae within humans are also modelled deterministically^21^. Adult female worms transition between non-fertile and fertile states to capture the 3-4 reproductively active cycles that female worms undergo per year, for which they need to be re-inseminated^47,48^. Microfilarial production takes place when at least one adult male and a fertile female worm are present, i.e., assuming a completely polygamous mating system and a balanced sex ratio^21,47^. The mortality rates of adult worms and microfilariae increase and the fecundity of female worms decreases with parasite age^21^. The default EPIONCHO-IBM human age- and sex-dependent exposure patterns are parameterised using data from African savannah epidemiological settings^25^ (Supplementary Fig. 1a). The model assumes density-dependent establishment/development of ingested microfilariae to L3 larvae within blackfly vectors, parasite-induced vector mortality (which increases with the number of microfilariae ingested by the blackflies), and transmission intensity-dependent establishment/development of inoculated L3 larvae to adult worms within humans^42^. Individual exposure to vector bites varies according to a gamma distribution with shape = rate parameter = *k*_*E*_, such that the mean exposure in the population is equal to 1. This ensures that blackfly bites are distributed among hosts with an average exposure given by the annual biting rate (ABR, the number of bites per person per year)^21^. Mass drug administration is modelled assuming that a proportion of eligible individuals (aged ≥5 years) receive treatment with ivermectin, which exerts a microfilaricidal effect and a temporary embryostatic effect^48^. A cumulative, irreversible sterilising effect per treatment dose^49^ is assumed from the second round of treatment onwards^27,50^. Figure 8 presents a schematic representation of EPIONCHO-IBM describing the parasite stages modelled in the blackfly vector and the human host, highlighting the stochastic and deterministic components of the model, as well as the processes (down- and up-) regulating parasite population abundance in vectors and humans. EPIONCHO-IBM is fully described in Hamley et al.^21^ and the code is available at: https://github.com/mrc-ide/EPIONCHO.IBM. All model simulations were run using R (version 4.4.3)^51^ and the Imperial College Research Computing Service^52^. See Data availability statement for a link providing source data and code to reproduce the analyses presented in this paper, available at: https://zenodo.org/records/21244449.

### Modelling anti-Ov16 IgG4 seroprevalence

We defined four hypotheses on the immunological (parasite stage) stimulus for production of detectable anti-Ov16 IgG4 antibodies (seroconversion): H1) following exposure to L3 larvae; H2) following the L4 to L5 moult (which occurs approximately 5 months following infection with L3)^53^; H3) following establishment of a single adult worm (of either sex); H4) following production of microfilariae (not necessarily detectable by skin-snip microscopy) corresponding to the presence of a mating worm pair (i.e., a fertile female and at least one male worm, assuming completely polygamous mating). Hypotheses H1-H3 reflect seroconversion during pre-patent infection; hypothesis H4 reflects seroconversion elicited by near-patent/patent infection (Fig. 2)^19^. Although patency in onchocerciasis is defined by the presence of skin microfilariae, hypothesis H4 accounts for any microfilariae in the host, whether detectable by skin-snip microscopy or not, allowing for an individual to be anti-Ov16 seropositive but skin-snip negative. We also considered three seroreversion hypotheses: i) no seroreversion (i.e., lifelong persistence of IgG4 antibodies); ii) seroreversion immediately following absence of the relevant seroconversion stimulus (i.e., immediate seroreversion with no immunological memory), and iii) seroreversion immediately following absence of all (larval and adult) parasite stages (i.e., finite seroreversion, following the absence of all parasite stages). The latter accounts phenomenologically for a finite (not lifelong) immunological memory, characterised by a cessation of IgG4 antibody production following complete clearance of infection. Thus, in total, we considered 12 hypotheses: four seroconversion hypotheses and for each of these, three seroreversion hypotheses (Table 1).

### Seroprevalence data

We used anti-Ov16 IgG4 seroprevalence data from Gabon^22^ to identify the best-fitting seroconversion-seroreversion hypotheses, and data from Togo^23^ for validation. From the treatment-naïve endemic setting in Gabon (i.e., in the absence of control intervention), 4350 individuals (aged 5-85 years, from 67 communities) consented to undergo skin-snip microscopy, of which 4257 (from 58 communities) also donated blood samples. The blood samples were tested in the field using an SD BIOLINE Ov16 RDT prototype. In the 4257 individuals, mean microfilarial prevalence and seroprevalence (both measured in those aged ≥5 years) were 7.90% (95% WCI: 7.13–8.75%) and 9.01% (95% WCI: 8.18–9.90%), respectively. The reported sensitivity and specificity of the Ov16-RDT with whole blood, compared to skin-snip microscopy performed in the same individuals, were 52% and 95%, respectively^22^. Microfilarial prevalence and anti-Ov16 IgG4 seroprevalence (estimated using an HRP Ov16 ELISA) from Togo were extracted from a published study conducted in 2015^23^, covering 11 villages (4 prefectures) located in the SIZ that followed the closure of the OCP^26^. The villages were: Pancerys (Kpendjal prefecture), Boutchakou and Koukoumbou (Ôti prefecture), Goulbi, Tchitchira, Koutougou Solla and Kpantiiyagou (Kéran prefecture), and Bawlesi, Mô-village, Katcha-Konkomba and Saboundi (Bassar prefecture)^23^. Kpendjal and Ôti prefectures are in the Savanes region; Kéran and Bassar prefectures are in the Kara region^26^. A total of 1455 people (aged 5–80 years) were examined by skin-snip microscopy, of whom 644 were tested using an HRP Ov16-ELISA, with age recorded in 576 individuals. The intervention history included vector control (1976–1993 in Savanes; 1976–2007 in Kara), and MDA from 1991^26^. The observed all-age microfilarial prevalence in 2015 (as reported by Komlan et al.^23^) was 5.7% (95% WCI: 4.5–6.9%) and the anti-Ov16 IgG4 seroprevalence was 34.0% (95% WCI: 30.3%-37.7%). Using skin-snip microscopy as the reference, the reported sensitivity and specificity of the HRP Ov16 ELISA used in Togo was 71.4% and 71.1%, respectively^23^.

### Model calibration

We calibrated EPIONCHO-IBM using the microfilarial data from Gabon (treatment-naïve, no prior MDA) and Togo (following 39 years of intervention). For Gabon, we ran 500 simulations for 100 years for each of a previously determined range of ABRs (which yielded endemic microfilarial prevalence between 0.05% and 21%) using two values of the inter-individual exposure heterogeneity parameter, *k*_*E*_ = 0.2 or *k*_*E*_ = 0.3 and their associated density dependence parameters^21,45^. We identified the ABR-*k*_*E*_ combinations that minimised the weighted MSE of the model predictions compared to age-stratified (sixteen 5-year age groups) microfilarial prevalence estimates (weighting squared errors by the proportion of the data within each age group; see Supplementary Methods 1 for further details). To account for potential bias arising from the assumed age- and sex-dependent exposure function in EPIONCHO-IBM (referred to as EPIONCHO exposure, reflecting its original development for the deterministic precursor of EPIONCHO-IBM^25^), we also calibrated the model using the ONCHOSIM age- and sex-dependent exposure function^13^. Briefly, the EPIONCHO exposure function assumes that males have higher exposure at younger ages, which gradually decreases, while females have lower exposure at birth, which increases with age. ONCHOSIM’s age- and sex-dependent exposure function assumes zero exposure at birth, which increases linearly until age 20, with males having higher overall exposure compared to females (Supplementary Fig. 1).

For Togo, the model was calibrated by grouping villages into prefectures (three villages in Ôti/Kpendjal, four villages in Kéran, and three villages in Bassar) due to a lack of village-specific intervention histories. Pancerys (in the Kpendjal prefecture) was grouped with the two other villages in the Ôti prefecture due to its location on the same river basin (Ôti River Basin), its proximity to the Ôti prefecture, and a shared history of vector control efforts, which ended in 1993 in both prefectures^26^. The baseline (pre-intervention) microfilarial prevalence had not been recorded for most villages (excepting Tchitchira in Kéran, which was holoendemic at baseline with 89.4% microfilarial prevalence, and Mô-village in Bassar, which was hyperendemic, with 71.4% microfilarial prevalence)^26^. Therefore, when baseline prevalence was not available, model-derived estimates from Amaral et al.^26^ were used (Supplementary Methods 1).

We ran 100 simulations for 100 years to reach endemic equilibrium for ABRs ranging from 100 to 60,000 bites/person/year using *k*_*E*_ = 0.3. For each prefecture, we constructed a distribution of ABRs that matched (within a 1-percent-point range; e.g., 70%-71%) the aggregated prefecture-specific pre-intervention microfilarial prevalence estimates that were either recorded or model-derived for that prefecture^26^. The latter were derived using EPIONCHO-IBM to simulate the history of intervention—under various assumptions of effectiveness defined by vector control efficacy, total population treatment coverage, and systematic non-adherence with MDA—in each prefecture (Supplementary Methods 2) and inferring the pre-intervention microfilarial prevalence that was compatible with modelled and observed microfilarial prevalence trends from repeated cross-sectional surveys undertaken between 1975 and 2015^26^. Supplementary Methods 1 and Amaral et al.^26^ provide further details.

### Hypothesis testing

We tested our 12 seroconversion-seroreversion hypotheses (Table 1) using EPIONCHO-IBM calibrated to the epidemiological data from Gabon by comparing the weighted MSEs of the predicted age-seroprevalence profiles against the observed age-stratified anti-Ov16 IgG4 seroprevalence estimates. We simulated the model 1500 times for 100 years (using the best ABR-*k*_*E*_ combinations from the calibration) for each hypothesis and adjusted the predicted anti-Ov16 seroprevalence for the uncertain sensitivity and specificity of the Ov16-RDT. Sensitivities and specificities were sampled from independent beta distributions, with mean and variance informed by reported sensitivity and specificity of IgG4 Ov16 RDTs using whole blood from other studies (Supplementary Table 3, Supplementary Fig. 4, Supplementary Methods 3). The predicted true seroprevalence from each run of the model was adjusted using 1500 pairs of sensitivity and specificity values. Adjustment was undertaken in a stochastic manner: putative seropositive individuals were randomly assigned a number from 0 to 1 using a uniform probability distribution, and considered as testing positive if their number was below the sampled sensitivity value (or negative if it was above, indicating a false negative result); putative seronegative individuals were considered positive if their number exceeded the sampled specificity value (indicating a false positive result).

Mathematically, for a given hypothesis, let ${Y}_{i,j} \in \{0,1\}$ denote the true (modelled) serostatus for individual j in a stochastic replicate *i* and for a diagnostic draw *d*, let $({{Se}}_{d},{{Sp}}_{d})$ be the sensitivity and specificity (drawn from independent beta distributions). The observed (diagnostically-adjusted) serostatus ${\tilde{Y}}_{i,d,j}\in \{0,1\}$ is given by,

1 $${\tilde{Y}}_{i,d,j} \sim {{\rm{Bernoulli}}}\left(S{e}_{d}{Y}_{i,j}+(1-S{p}_{d})(1-{Y}_{i,j})\right)$$

If we further let A be the set of 5-year age bins and *g*(·) be a function that maps modelled integer age to its bin, then for bin *a* ∈ *A*, the set of simulated individuals in that bin for replicate *i* is given by,

2 $${S}_{i}(a)=\{\,j:g({{{\rm{age}}}}_{i,j})=a\},\,{N}_{i}(a)=|{S}_{i}(a)|$$

It follows that the adjusted model-predicted seroprevalence in age bin a for replicate i and diagnostic draw d is,

3 $${\tilde{p}}_{i,d}(a)=\frac{1}{{N}_{i}(a)}{\sum}_{j\in {S}_{i}(a)}{\tilde{Y}}_{i,d,j}$$

and the average seroprevalence in age bin a is,

4 $$\tilde{p}(a)=\frac{1}{ID}{\sum }_{i=1}^{I}{\sum }_{d=1}^{D}{\tilde{p}}_{i,d}(a)$$

Where $D={\mathrm{1 500}}$ is the number of sensitivity and specificity replicates and $I={\mathrm{1 500}}$ is the number of model simulation replicates.

### Weighted mean squared error calculation

Weighted MSEs were calculated individually for each model run, sensitivity and specificity pair, seroconversion, and seroreversion hypothesis, by comparing the predicted age-seroprevalence with the observed age-seroprevalence. The age-MSEs were weighted by the percentage of the observed population within that age group and summed to create a single weighted MSE for a given run with a given diagnostic adjustment pair. From there, the mean MSE was calculated for each diagnostic performance pair across each run. Finally, the mean diagnostic performance-weighted MSE for each diagnostic performance pair were combined to calculate the mean weighted MSE, and 95% Monte Carlo confidence interval (95% MCCI; capturing stochastic uncertainty), for each seroconversion and seroreversion hypothesis.

Mathematically, let ${p}^{{{\rm{obs}}}}(a)$ be the observed seroprevalence in age bin a and let ${n}^{{{\rm{obs}}}}(a)$ be the observed sample size. We define the weights as,

5 $$w(a)=\frac{{n}^{{{\rm{obs}}}}(a)\,}{{\sum }_{a^{\prime}\in A}{n}^{{{\rm{obs}}}}(a^{\prime})}$$

such that the weighted MSE for model replicate i and diagnostic draw d is given by,

6 $$MS{E}_{i,d}={\sum}_{a\in A}w(a){\left({\tilde{p}}_{i,d}(a)-{p}^{{{\rm{obs}}}}(a)\right)}^{2}$$

The mean weighted MSE across $D={\mathrm{1 500}}$ diagnostic (sensitivity/specificity) draws and $I={\mathrm{1 500}}$ stochastic model replicates is,

7 $$\overline{MSE}=\frac{1}{ID}{\sum }_{i=1}^{I}{\sum }_{d=1}^{D}MS{E}_{i,d}$$

with 95% MCCI calculated based on the variance across stochastic repeats {${{{\rm{MSE}}}}_{i}$},

8 $$MCC{I}_{95\%}=\overline{MSE}\,\pm 1.96\times \frac{VAR(MS{E}_{i})}{I}$$

For interpretability we report normalised MSE relative to the best-performing hypothesis (i.e., ΔMSE scaled to the best fit), using MSEs that are marginalised over uncertainty in diagnostic sensitivity and specificity. We emphasize that differences in MSEs across diagnostic assumptions are used only for robustness assessment and do not constitute evidence about the true diagnostic performance, which is not identifiable from these data alone.

### Model validation

We used the best fitting seroconversion-seroreversion hypotheses (smallest MSE) from hypothesis testing to compare the predicted age-seroprevalence profiles (under intervention) with the observed seroprevalence from Togo collected in 2015^23^. Using the distributions of ABRs from the model calibration, we simulated endemic equilibrium for each prefecture and projected forward to 2015, incorporating 39 years of interventions. These included the prefecture-specific history of vector control (from 1976–2007, except in Ôti/Kpendjal, where it ceased in 1993), MDA frequency between 1991 and 2015 (Supplementary Table 8), and intervention effectiveness—defined by vector control efficacy, MDA coverage, and the proportion of systematic non-adherence (i.e., individuals never treated)—as estimated by Amaral et al.^26^ (Supplementary Table 8). A detailed description of the intervention histories and how they were modelled is given in Supplementary Methods 2.

We compared the prefecture-specific model predictions with the all-age anti-Ov16 IgG4 seroprevalence. Because the sensitivity of the HRP Ov16-ELISA used in Togo was estimated by comparing against presence of microfilariae—which has poor sensitivity in near-elimination settings^5,10,33^—we calculated the MSE of the model predictions using sensitivities and specificities sampled from beta distributions, parameterised using values compiled from prior studies of HRP Ov16-ELISA protocols (Supplementary Table 10, Supplementary Fig. 9, Supplementary Methods 3). Similarly to hypothesis testing, 1500 sensitivity and specificity pairs were sampled from independent beta distributions (Supplementary Fig. 9), and each run was adjusted by each sensitivity/specificity pair. Supplementary Table 10 lists the studies used to inform the distribution of sensitivity and specificity.

An age-stratified analysis at prefecture level was not possible as data disaggregated by age were not reported for each prefecture. Thus, we aggregated model outputs across prefectures, sampling individuals in proportion to the observed parasitological or serological data (324/1455 = 22.3% for microfilarial prevalence and 254/644 = 39.4% for anti-Ov16 IgG4 seroprevalence in Ôti/Kpendjal; 539/1455 = 37.0% and 355/644 = 55.1% in Kéran; 592/1455 = 40.7% and 35/644 = 5.43% in Bassar). For the calculation of prefecture-specific MSEs, we collapsed the age stratification used in Eqs. (5–8) to a single all-age bin (i.e., *A* = {5–80}). In this case, the weighting over age bins is trivial (the single bin has weight 1), and the resulting MSE compares the model-predicted all-age (5–80 years) seroprevalence with the observed all-age seroprevalence for that prefecture. To summarise predictive performance across prefectures, we combined prefecture-specific MSEs using weights proportional to each prefecture’s contribution to the observed serology sample so that the weighting in Eqs. (5–8) is applied over prefectures rather than over age bins.

All reported values were rounded to 3 significant figures with the exception of those values taken from the literature, which are given with their reported precision.

We adhered to the five principles of the Neglected Tropical Diseases (NTD) Modelling Consortium regarding Policy-Relevant Items for Reporting Models in Epidemiology of NTDs (PRIME-NTD), for good practice in NTD modelling^54^ (Supplementary Table 15).

### Reporting summary

Further information on research design is available in the Nature Portfolio Reporting Summary linked to this article.

## Supplementary information

Supplementary Information

Peer Review file

Reporting Summary

## Data Availability

All information used for the analyses is contained in the figures, tables, and Supplementary Information. The data used to calibrate and validate the model are publicly available in the publications by Atsame et al. 2024^22^, Komlan et al. 2018^23^ and Amaral et al.^26^. The following link describes the source data and the code to reproduce the analyses presented: https://doi.org/10.5281/zenodo.21244449^55^

## Code availability

The EPIONCHO-IBM model code and instructions to replicate the analyses conducted and the results obtained in this study are available at: https://github.com/mrc-ide/EPIONCHO.IBM/tree/v1.0.1-ov16. Instructions on how to use the code are contained within the accompanying README file. The source data and code to replicate the analyses can also be found at: https://doi.org/10.5281/zenodo.21244449^55^.

The version of the EPIONCHO-IBM model with the best-fit hypotheses from this paper is available at: https://github.com/mrc-ide/EPIONCHO.IBM/tree/master. Steps on how to use the model code are given in the vignettes.
