# Supplementary material for: Modelling anti-Ov16 seroprevalence for the control and elimination of onchocerciasis
Source: Nat Commun. 2026 Aug 19;17:9924. doi: 10.1038/s41467-026-76562-9 (PMC13582928; doi:10.1038/s41467-026-76562-9)
Supplement: Supplementary file 1 [file 41467_2026_76562_MOESM1_ESM.pdf]

Supplementary Information

Modelling anti-Ov16 seroprevalence for the control and elimination of onchocerciasis

Aditya Ramani[1,2,], Jacob N. Stapley[2], Matthew A. Dixon[2], Jonathan I. D. Hamley[2,3,4], Luís-Jorge Amaral[2,5], Maria-Gloria Basáñez[2,*,†], Martin Walker[1,2,*,†]

[1] Department of Pathobiology and Population Sciences, Royal Veterinary College, Hawkshead Lane, Hatfield, United Kingdom

[2] MRC Centre for Global Infectious Disease Analysis, Department of Infectious Disease Epidemiology, School of Public Health, Imperial College London, London, United Kingdom

[3] Department of Visceral Surgery and Medicine, Inselspital, Bern University Hospital, University of Bern, Switzerland

[4] Multidisciplinary Center for Infectious Diseases, University of Bern, Bern, Switzerland

[5] Global Health Institute, University of Antwerp, Antwerp, Belgium

[*] **Corresponding authors:** m.basanez@imperial.ac.uk; mwalker@rvc.ac.uk

[†] Joint senior authors

Supplementary Figures and Tables

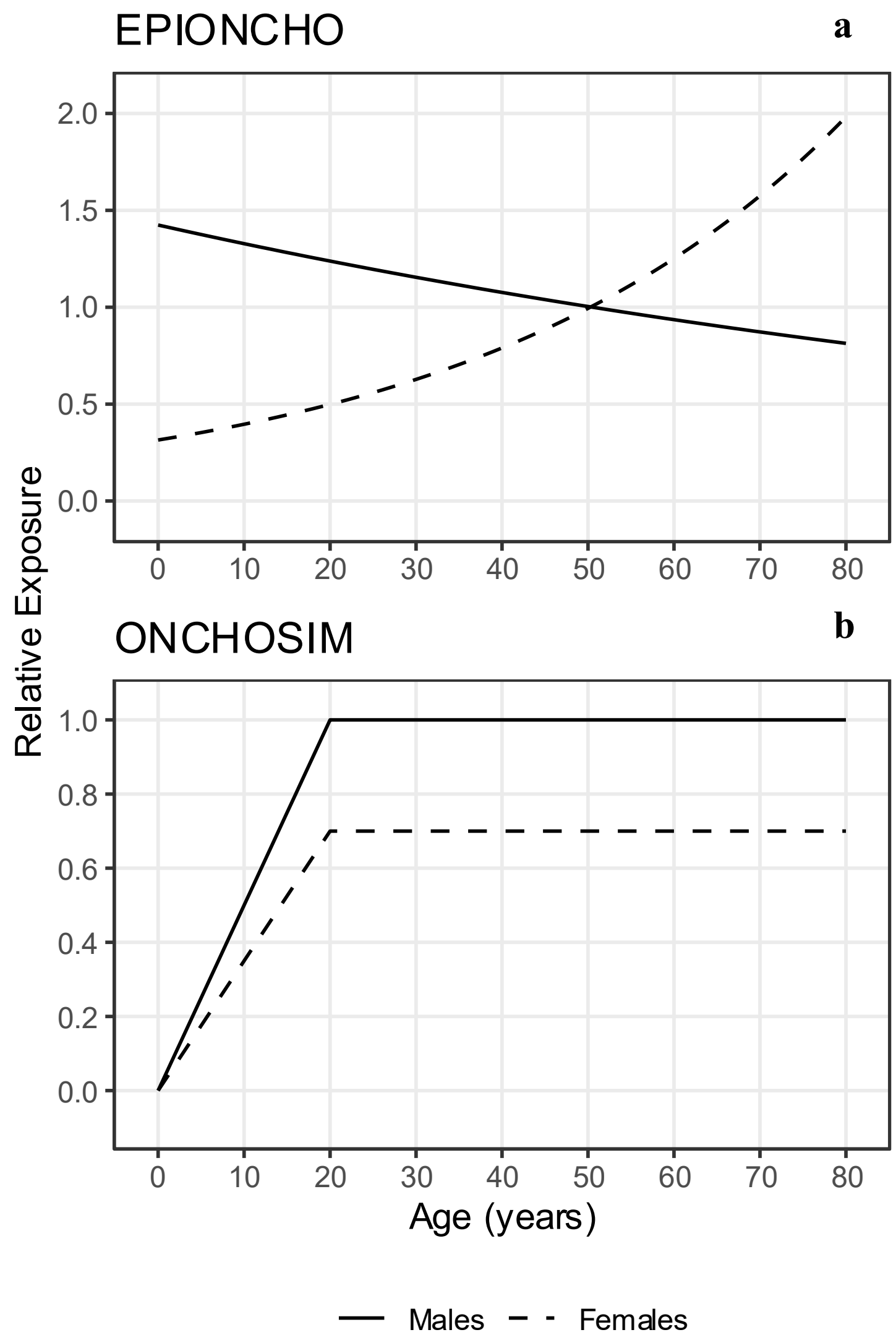

Supplementary Fig. 1. EPIONCHO and ONCHOSIM age- and sex-dependent exposure functions. a The EPIONCHO exposure function assumes non-zero exposure at birth, and variable exposure throughout life, with males' relative exposure decreasing and females' relative exposure increasing with age[1]. **b** ONCHOSIM exposure is zero at birth, increases at a constant rate until age 20, whereafter it plateaus. Females have a consistently lower exposure than males[2]. Figure modified from Hamley et al.[3]

Supplementary Table 1. Parameters used for calibrating EPIONCHO-IBM to observed microfilarial prevalence data from 4,257 individuals aged 5-90 years living in 58 onchocerciasis hypoendemic (treatment-naïve) communities in Gabon[4]

Age- and sex-dependent exposure function	Annual biting rate (ABR, no. bites/person/year) prior range	Inter-individual exposure heterogeneity parameter, k_E	ABR best-fit value (range)[a]
EPIONCHO[b]	60 – 120	0.2	76 (74 – 76)
	150 – 210	0.3	179 (176 – 180)
ONCHOSIM[b]	50 – 130	0.2	70 (70 – 72)
	120 – 210	0.3	164 (162 – 166)

[a] Range of ABRs within 95% Wilson score 95% confidence interval of observed prevalence estimate (7.13%−8.75%)[4].

[b] See Supplementary Fig. 1 for EPIONCHO and ONCHOSIM exposure functions.

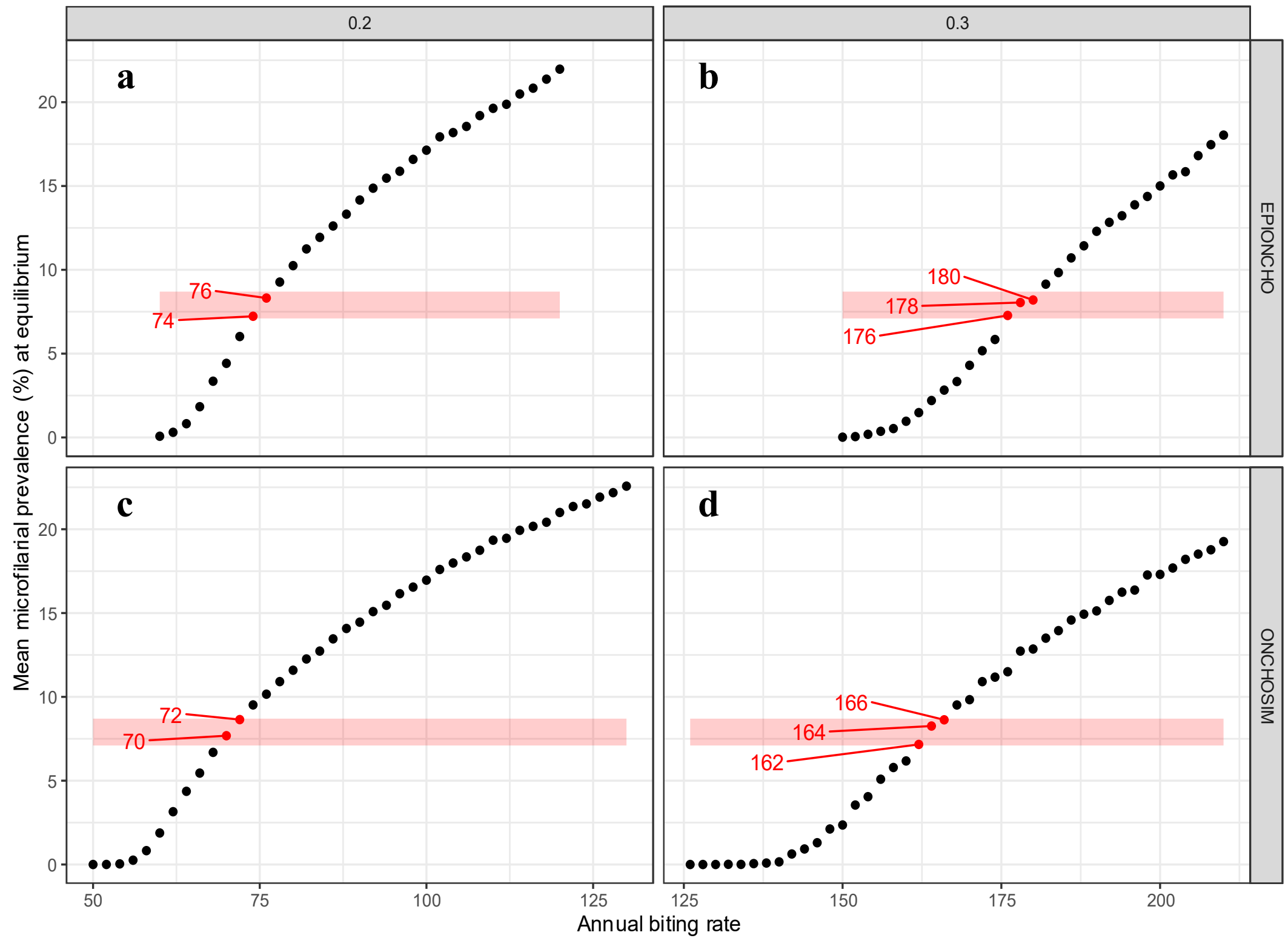

Supplementary Fig. 2. Observed and predicted microfilarial prevalence in individuals aged 5 years and older for different combinations of the annual biting rate (ABR) and inter-individual exposure heterogeneity parameter, k_E. The predicted microfilarial prevalence values (black circles) were generated using EPIONCHO-IBM after a 100-year burn-in period, with age- and sex-dependent (EPIONCHO or ONCHOSIM) exposure (Supplementary Fig. 1). The ABR-k_E values within the 95% Wilson score confidence interval of the observed microfilarial prevalence estimate from 4,257 individuals from 58 hypoendemic treatment-naïve communities in Gabon[4] is shaded in red. The predicted microfilarial prevalences were calculated using the mean of 100 simulations of the model for a given ABR, weighting the prevalence in 5-year age groups by the percentage of individuals in that age group within the observed data. **a** Results using EPIONCHO exposure[1,3] and $k_E = 0.2$. **b** Results using EPIONCHO exposure[1,3] and $k_E = 0.3$. **c** Results using ONCHOSIM exposure[2,3] and $k_E = 0.2$. **d** Results using ONCHOSIM exposure[2,3] and $k_E = 0.3$. The best-fit ABR was 76 bites/person/year and $k_E = 0.2$ when using EPIONCHO exposure, and 70 bites/person/year and $k_E = 0.2$ when using ONCHOSIM exposure.

Supplementary Table 2. Parameters used for calibrating EPIONCHO-IBM to model-derived pre-intervention microfilarial prevalence estimates from 1,455 individuals aged 5-80 years living in four prefectures in Togo[5]

Prefecture	Number of individuals examined by skin-snip microscopy	Inferred pre-intervention microfilarial prevalence estimate (endemicity level)[a]	Annual biting rate range (no. bites/person/year)[b]
Ôti/Kpendjal	324	70% (hyperendemic)	1,000 – 5,000
Kéran	539	85% (holoendemic)	6,000 – 60,000
Bassar	592	70% (hyperendemic)	1,000 – 5,000

[a] Inferred by aggregating model-derived village-level estimates presented in Amaral et al.[6]
[b] Only EPIONCHO exposure was used.

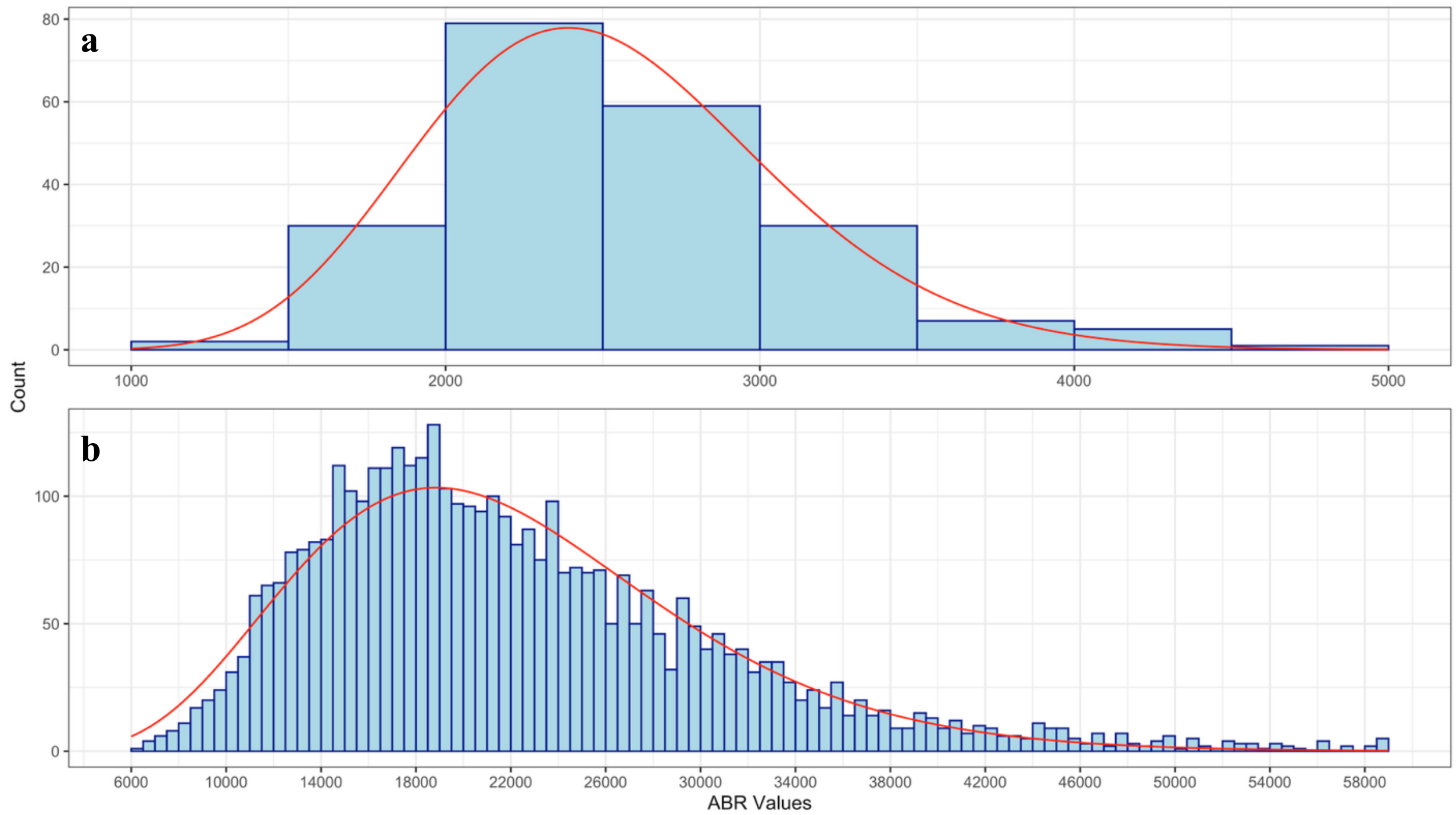

Supplementary Fig. 3. Distribution of annual biting rates (ABRs) derived from calibrating EPIONCHO-IBM to three prefectures in Togo with pre-intervention microfilarial prevalence values of 70% and 85%. The blue bars show the frequency of ABRs that yielded an endemic equilibrium microfilarial prevalence that was within ± 0.5% of the target. **a** 70% microfilarial prevalence: Ôti/Kpendjal and Bassar prefectures. **b** 85% microfilarial prevalence: Kéran prefecture. See Supplementary Table 2 and Supplementary Methods 1 for detailed information on how these target microfilarial prevalences were determined. The red lines show the fitted gamma distributions to the empirical ABRs, estimated using the fitdistrplus package[7] in R. These parameterised gamma distributions were used for sampling ABRs.

Supplementary Table 3. Sensitivity and specificity estimates for the SD BIOLINE Ov16 rapid diagnostic test (Ov16-RDT) using whole blood

Sensitivity	Specificity	Reference diagnostic	Sample size	Reference
51.0%	94.8%	Skin-snip microscopy	4,257	Atsame et al.[4]
60.0%	94.0%	Skin-snip microscopy	400	Nikièma et al.[8]
40.0%	98.9%	Ov16 ELISA (Tanzania)	931	Ogawa et al.[9]
28.4%	99.9%	Ov16 ELISA (Togo)	999	Ogawa et al.[9]
40.0% – 60.0%	–	Skin-snip microscopy	–	NTDSC, TFGH[10]

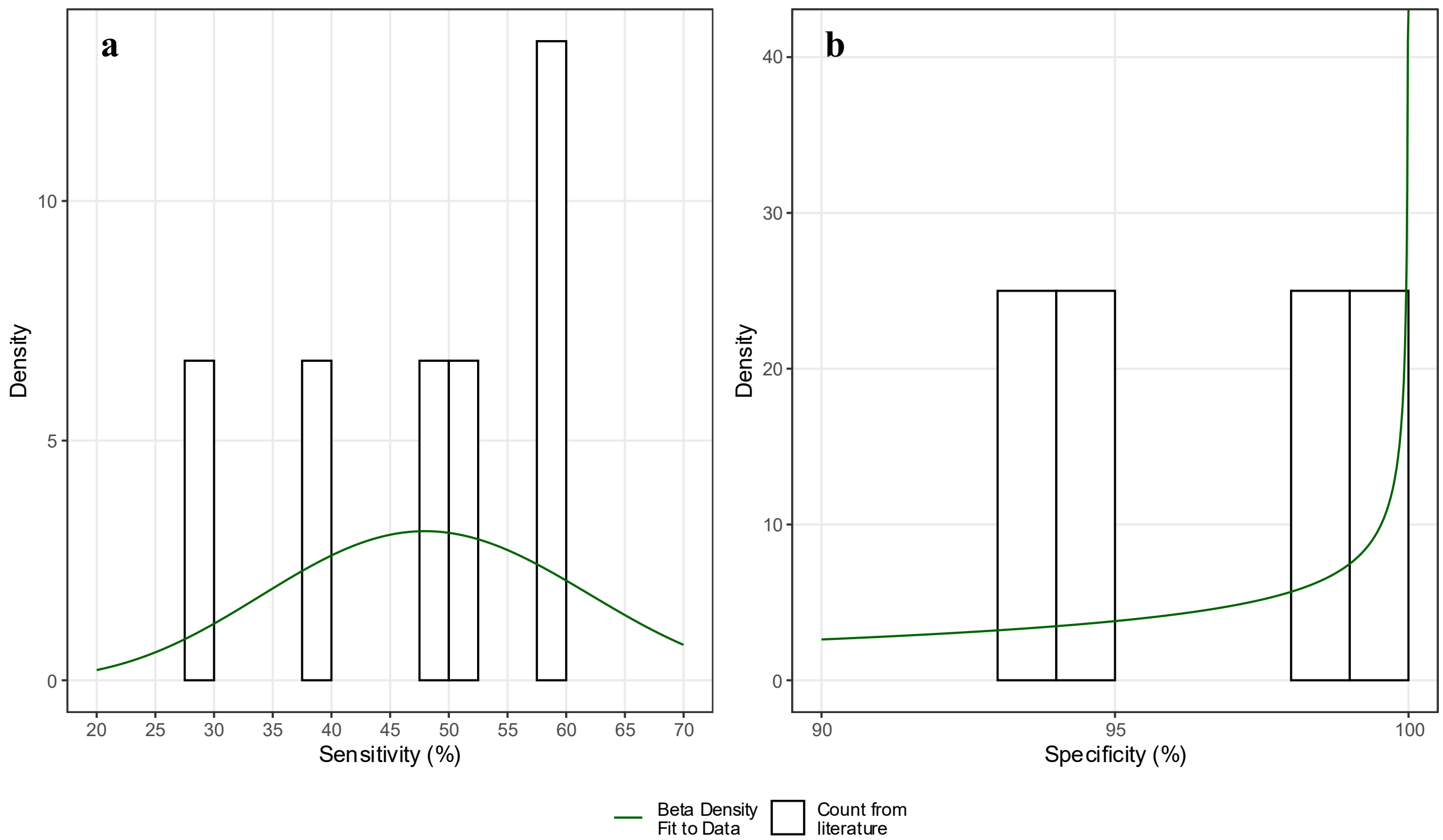

Supplementary Fig. 4. Distribution of sensitivity and specificity of the Ov16 SD BIOLINE rapid diagnostic test (Ov16-RDT) using whole blood. White bars are reported values from the literature (Supplementary Table 3). Green lines are fitted beta distributions (Supplementary Methods 3). **a** Sensitivity, with shape parameters $\alpha = 7.56$, $\beta = 8.11$ (mean = 48.2%). **b** Specificity, with shape parameters $\alpha = 32.85$, $\beta = 1.05$ (mean = 96.9%).

Supplementary Table 4. Quantitative assessment of goodness-of-fit of EPIONCHO-IBM-predicted versus observed anti-Ov16 IgG4 seroprevalence from 4,257 individuals living in 58 hypoendemic communities in Gabon[4] for each seroconversion-seroreversion hypothesis using EPIONCHO exposure[1,3] and incorporating uncertainty in diagnostic performance of the SD BIOLINE Ov16-RDT

Sensitivity[a]	Specificity[a]	Hypothesis	Unnormalised mean squared error (MSE)[b] (95% Monte Carlo confidence interval)		
			No seroreversion (i)	Immediate seroreversion (ii)	Finite seroreversion (iii)
48.2% (24.8%–72.1%)	96.9% (89.1%–99.9%)	H1	183 (176–189)	54.9 (53.7–56.1)	69.1 (65.9–72.3)
		H2	181 (174–187)	54.9 (53.7–56.1)	65.8 (62.7–68.9)
		H3	178 (172–185)	62.8 (59.8–65.8)	62.6 (59.7–65.6)
		H4	33.7 (31.7–35.7)	20.6 (19.7–21.5)	**18.1**[c] **(16.8–19.4)**[c]

[a] Beta distribution mean (Supplementary Fig. 4) and 2.5th – 97.5th percentiles.

[b] Mean squared error (MSE) is calculated as the arithmetic mean squared error (residual) and 95% Monte Carlo confidence interval from 1,500 model repeats (stochastic simulations) marginalised over 1,500 sensitivity/specificity replicates weighted by the proportion of observations within each age group.

[c] Best-fitting hypothesis (lowest MSE).

Supplementary Table 5. Quantitative assessment of goodness-of-fit of EPIONCHO-IBM-predicted versus observed anti-Ov16 IgG4 seroprevalence from 4,257 individuals living in 58 hypoendemic communities in Gabon[4] for each seroconversion-seroreversion hypothesis using ONCHOSIM exposure[2,3] and incorporating uncertainty in diagnostic performance of the SD BIOLINE Ov16-RDT

**Sensitivity**[a]	**Specificity**[a]	**Hypothesis**	**Unnormalised mean squared error (MSE)**[b] **(95% Monte Carlo confidence interval)**		
			**No seroreversion (i)**	**Immediate seroreversion (ii)**	**Finite seroreversion (iii)**
48.2% (24.8%–72.1%)	96.9% (89.1%–99.9%)	H1	160 (154–166)	54.8 (53.6–56.0)	58.7 (55.8–61.6)
		H2	158 (152–164)	54.8 (53.6–56.0)	55.8 (53.1–58.6)
		H3	156 (150–162)	53 (50.3–55.7)	53.1 (50.4–55.7)
		H4	33.5 (31.7–35.3)	**20.8**[c] **(19.9–21.7)**[c]	**19.0**[c] **(17.8–20.1)**[c]

[a] Beta distribution mean (Supplementary Fig. 4) and 2.5th – 97.5th percentile.

[b] Mean squared error (MSE) is calculated as the arithmetic mean squared error (residual) and 95% Monte Carlo confidence interval from 1,500 model repeats (stochastic simulations) marginalised over 1,500 sensitivity/specificity replicates weighted by the proportion of observations within each age group.

[c] Best-fitting hypotheses (lowest MSE).

Supplementary Table 6. Quantitative assessment of goodness-of-fit of EPIONCHO-IBM-predicted versus observed anti-Ov16 IgG4 seroprevalence from 4,257 individuals living in 58 hypoendemic communities in Gabon[4] for each seroconversion-seroreversion hypothesis using EPIONCHO exposure[1,3] and assuming different sensitivities and specificities of the SD BIOLINE Ov16-RDT

Sensitivity	Specificity	Hypothesis	Unnormalised mean squared error (MSE)[a] (95% confidence interval)		
			No seroreversion (i)	Immediate seroreversion (ii)	Finite seroreversion (iii)
40%	99%	H1	63.4 (62.8–64)	74.9 (74.7–75)	16.4 (16.2–16.6)
		H2	62.4 (61.8–63)	74.9 (74.7–75)	15.3 (15.1–15.6)
		H3	61.3 (60.7–61.9)	14.2 (14–14.4)	14.3 (14.1–14.6)
		H4	**6.65 (6.53–6.78)**[b]	28.6 (28.4–28.9)	10.7 (10.5–10.9)
50%		H1	141 (140–142)	74.6 (74.5–74.8)	38.4 (38–38.9)
		H2	139 (138–140)	74.6 (74.5–74.8)	35.9 (35.4–36.3)
		H3	137 (136–138)	33.4 (33–33.8)	33.4 (33–33.8)
		H4	13.6 (13.4–13.9)	20.8 (20.5–21)	**6.49 (6.36–6.62)**[b]
60%		H1	252 (251–253)	74.8 (74.6–75)	75.9 (75.2–76.6)
		H2	249 (248–250)	74.8 (74.6–75)	71.3 (70.6–72)
		H3	246 (244–247)	66.8 (66.1–67.5)	66.8 (66.2–67.5)
		H4	30.6 (30.2–31)	14.9 (14.6–15.1)	**7.31 (7.16–7.46)**[b]
40%	97%	H1	81.9 (81.3–82.6)	48 (47.7–48.2)	26.9 (26.6–27.2)
		H2	80.7 (80–81.3)	48 (47.7–48.2)	25.4 (25.1–25.7)
		H3	79.4 (78.8–80)	23.6 (23.3–23.9)	23.9 (23.6–24.1)
		H4	9.32 (9.15–9.49)	16.1 (15.9–16.4)	**7.15 (7.02–7.29)**[b]
50%		H1	168 (167–169)	47.8 (47.6–48)	56.8 (56.2–57.3)
		H2	166 (165–167)	47.8 (47.6–48)	53.7 (53.1–54.2)
		H3	163 (162–164)	50.4 (49.9–50.9)	50.5 (50–51.1)
		H4	22.1 (21.8–22.4)	11.7 (11.5–11.9)	**7.68 (7.54–7.83)**[b]
60%		H1	287 (286–289)	47.8 (47.5–48)	102 (101–103)
		H2	284 (282–285)	47.7 (47.5–47.9)	96.5 (95.7–97.3)
		H3	280 (279–282)	91.3 (90.5–92.1)	91.4 (90.6–92.1)
		H4	45.1 (44.5–45.6)	**8.7 (8.54–8.86)**[b]	13.4 (13.2–13.6)
40%	94%	H1	116 (115–117)	22 (21.8–22.2)	50.3 (49.8–50.7)
		H2	115 (114–115)	22 (21.8–22.2)	48.1 (47.7–48.6)
		H3	113 (112–114)	45.9 (45.5–46.4)	46 (45.6–46.4)
		H4	22.8 (22.5–23.1)	**10 (9.86–10.2)**[b]	12.3 (12.2–12.5)
50%		H1	217 (215–218)	21.9 (21.6–22.1)	92 (91.3–92.7)
		H2	214 (213 215)	21.9 (21.6–22.1)	88.1 (87.4–88.8)
		H3	211 (210–213)	84.8 (84.1–85.5)	84.2 (83.6–84.9)
		H4	44.7 (44.2–45.2)	**10.2 (10–10.4)**[b]	20.5 (20.2–20.8)
60%		H1	350 (348–351)	22 (21.8–22.3)	150 (149–151)
		H2	346 (344–347)	22 (21.8–22.3)	144 (143–145)
		H3	342 (340–344)	137 (136–138)	137 (136–138)
		H4	77.2 (76.5–77.9)	**12.4 (12.2–12.6)**[b]	33.8 (33.3–34.2)

[a] Mean squared error (MSE) is calculated as the arithmetic mean squared error (residual) and 95% Monte Carlo confidence interval from 1,500 model repeats (stochastic simulations) weighted by the proportion of observations within each age group.

[b] Best-fitting hypothesis (lowest MSE) for each combination of sensitivity and specificity.

Supplementary Table 7. Quantitative assessment of goodness-of-fit of EPIONCHO-IBM-predicted versus observed anti-Ov16 IgG4 seroprevalence from 4,257 individuals living in 58 hypoendemic communities in Gabon[4] for each seroconversion-seroreversion hypothesis using ONCHOSIM exposure[2,3] and assuming different sensitivities and specificities of the SD BIOLINE Ov16-RDT

Sensitivity	Specificity	Hypothesis	Unnormalised mean squared error (MSE)[a] (95% confidence interval)		
			No seroreversion (i)	Immediate seroreversion (ii)	Finite seroreversion (iii)
40%	99%	H1	53.6 (53–54.1)	74.9 (74.8–75.1)	12.2 (12–12.4)
		H2	52.8 (52.3–53.3)	74.9 (74.8–75.1)	11.5 (11.3–11.7)
		H3	52.1 (51.6–52.7)	10.8 (10.6–11)	10.8 (10.6–11)
		H4	**10.3 (10.2–10.5)**[b]	29.9 (29.6–30.1)	13.5 (13.3–13.7)
50%		H1	122 (121–123)	75 (74.8–75.1)	31.1 (30.8–31.5)
		H2	120 (120–121)	74.9 (74.8–75.1)	29 (28.6–29.4)
		H3	119 (118–120)	26.8 (26.5–27.1)	26.9 (26.6–27.3)
		H4	17.1 (16.8–17.3)	22.6 (22.3–22.8)	**9.93 (9.78–10.1)**[b]
60%		H1	219 (218–220)	74.8 (74.6–74.9)	64 (63.3–64.6)
		H2	216 (215–218)	74.8 (74.6–74.9)	60 (59.4–60.6)
		H3	214 (212–215)	55.9 (55.4–56.5)	56.2 (55.6–56.8)
		H4	32.7 (32.3–33.2)	16.7 (16.4–16.9)	**11.4 (11.2–11.6)**[b]
40%	97%	H1	68.6 (68–69.2)	47.7 (47.4–47.9)	20.6 (20.3–20.9)
		H2	67.6 (67–68.2)	47.7 (47.4–47.9)	19.3 (19–19.5)
		H3	66.6 (66–67.2)	17.9 (17.6–18.1)	18.1 (17.8–18.3)
		H4	10 (9.83–10.2)	16.4 (16.2–16.6)	**8.05 (7.91–8.19)**[b]
50%		H1	145 (144–146)	47.8 (47.6–48.1)	46.4 (46–46.9)
		H2	143 (142–144)	47.8 (47.6–48)	43.7 (43.3–44.2)
		H3	141 (141–142)	40.7 (40.2–41.1)	41 (40.6–41.5)
		H4	22.2 (21.8–22.5)	11.8 (11.7–12)	**8.87 (8.71–9.03)**[b]
60%		H1	252 (250–253)	47.7 (47.4–47.9)	86.6 (85.8–87.3)
		H2	249 (247–250)	47.7 (47.4–47.9)	81.9 (81.2–82.6)
		H3	246 (244–247)	77.5 (76.8–78.2)	77.3 (76.6–78)
		H4	43.8 (43.3–44.3)	**8.97 (8.8–9.13)**[b]	14.7 (14.5–15)
40%	94%	H1	98.3 (97.5–99)	21.9 (21.7–22.1)	41.2 (40.8–41.6)
		H2	96.9 (96.1–97.6)	21.9 (21.7–22.1)	39.3 (38.9–39.7)
		H3	95.5 (94.8–96.2)	37.4 (37–37.8)	37.3 (36.9–37.7)
		H4	19.8 (19.6–20.1)	**8.78 (8.62–8.93)**[b]	10.7 (10.6–10.9)
50%		H1	187 (185–188)	22 (21.8–22.2)	78.2 (77.6–78.9)
		H2	184 (183–185)	21.9 (21.7–22.2)	74.6 (74–75.2)
		H3	182 (181–183)	70.8 (70.1–71.4)	71 (70.4–71.7)
		H4	40 (39.5–40.5)	**8.53 (8.39–8.68)**[b]	18.4 (18.1–18.7)
60%		H1	305 (304–307)	22 (21.8–22.2)	129 (128–130)
		H2	302 (300–303)	22 (21.8–22.2)	124 (123–125)
		H3	298 (297–300)	118 (117–119)	118 (117–119)
		H4	69.6 (68.9–70.3)	**10.1 (9.92–10.2)**[b]	30.9 (30.6–31.3)

[a] Mean squared error (MSE) is calculated as the arithmetic mean squared error (residual) and 95% Monte Carlo confidence interval from 1,500 model repeats (stochastic simulations) weighted by the proportion of observations within each age group.

[b] Best-fitting hypothesis (lowest MSE) for each combination of sensitivity and specificity.

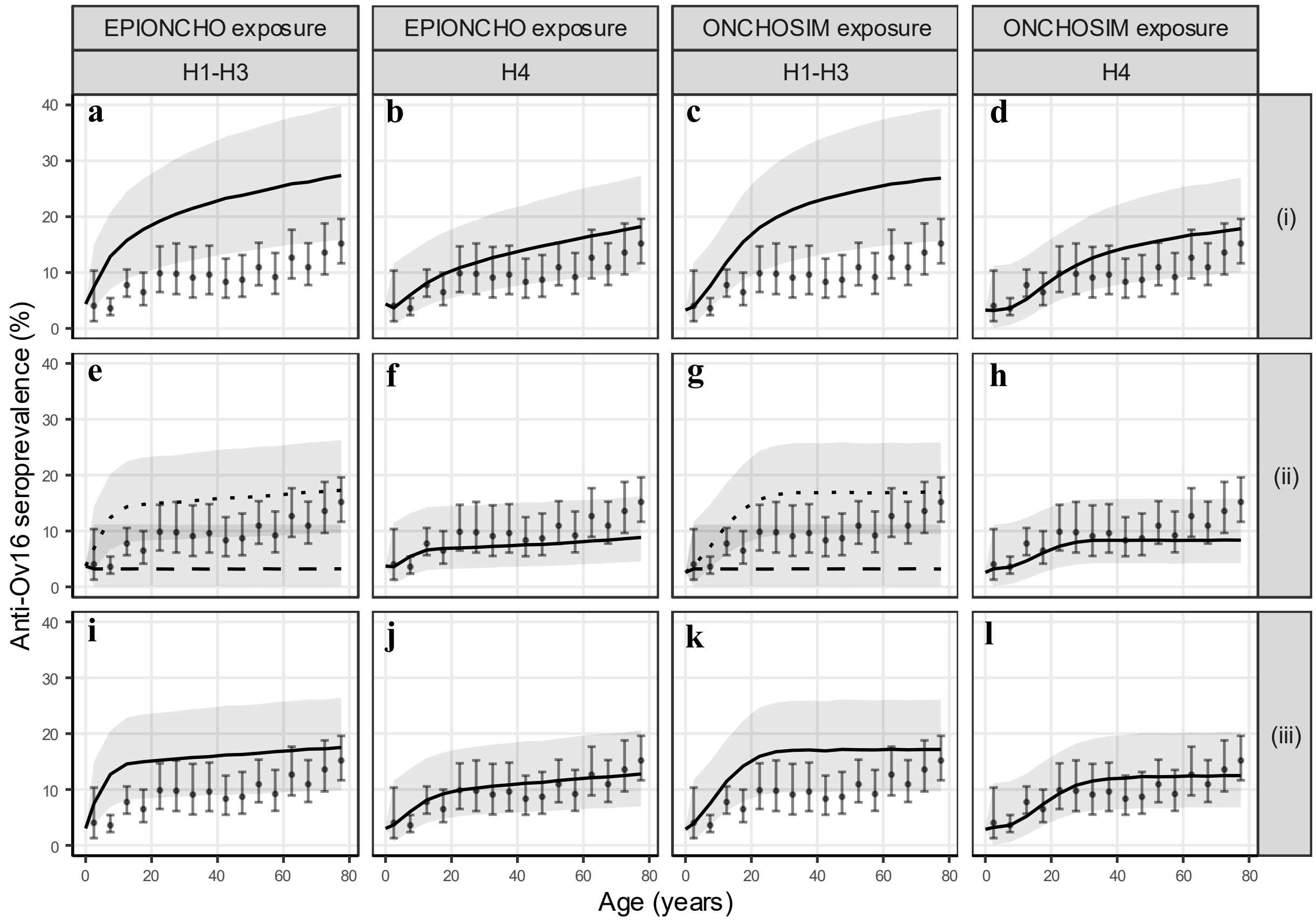

Supplementary Fig. 5. **Observed and predicted anti-Ov16 IgG4 age-seroprevalence profiles for 4,257 individuals living in 58 ivermectin-naïve communities in Gabon[4].** Mean predicted age-seroprevalence using EPIONCHO-IBM with exposure heterogeneity parameter k_E=0.2, calibrated to microfilarial prevalence estimates[4] (Fig. 1 of Main Text). **a** EPIONCHO exposure[1,3]; annual biting rate (ABR)=76 bites/person/year; anti-Ov16 IgG4 seroconversion elicited by pre-patent-infection with no seroreversion (hypotheses H1i-H3i). **b** EPIONCHO exposure; ABR=76; seroconversion elicited by near-patent/patent infection with no seroreversion (hypothesis H4i). **c** ONCHOSIM exposure[2,3]; ABR=70; H1i-H3i. **d** ONCHOSIM exposure; ABR=70; H4i. **e** EPIONCHO exposure; ABR=76; seroconversion elicited by pre-patent infection with immediate seroreversion (H1ii-H3ii). **f** EPIONCHO exposure; ABR=76; seroconversion elicited by near-patent/patent infection with immediate seroreversion (H4ii). **g** ONCHOSIM exposure; ABR=70; H1ii-H3ii. **h** ONCHOSIM exposure; ABR=70; H4ii. **i** EPIONCHO exposure; ABR=76; seroconversion elicited by pre-patent infection with finite seroreversion (H1iii-H3iii). **j** EPIONCHO exposure; ABR=76; seroconversion elicited by near-patent/patent infection with finite seroreversion (H4iii). **k** ONCHOSIM exposure; ABR=70; H1iii-H3iii. **l** ONCHOSIM exposure; ABR=70; H4iii. Solid black lines for H1-H3 indicate no appreciable difference in projections between these hypotheses (**a, c, i, k**). Dashed lines indicate H1ii-H2ii; dotted lines denote H3ii, for immediate seroreversion (**e**, **g**). Projections were adjusted for the assumed diagnostic performance of the SD BIOLINE Ov16 rapid diagnostic test using whole blood. Diagnostic parameters were sampled from independent beta distributions, with mean sensitivity=48.2% and mean specificity=96.9% (Supplementary Fig. 4). Black circles are observed seroprevalence estimates binned into 5-year age groups; error bars are 95% Wilson score confidence intervals. Shaded areas depict 95% uncertainty intervals (2.5th to 97.5th quantiles of the simulated mean), based on 1,500 stochastic repeats, each adjusted by 1,500 replicates from the diagnostic performance distributions. See Supplementary Fig. 1 for EPIONCHO and ONCHOSIM exposure functions. Source data originate from Atsame et al.[4]; the code to reproduce the figure with model output is in Ramani et al.[23]

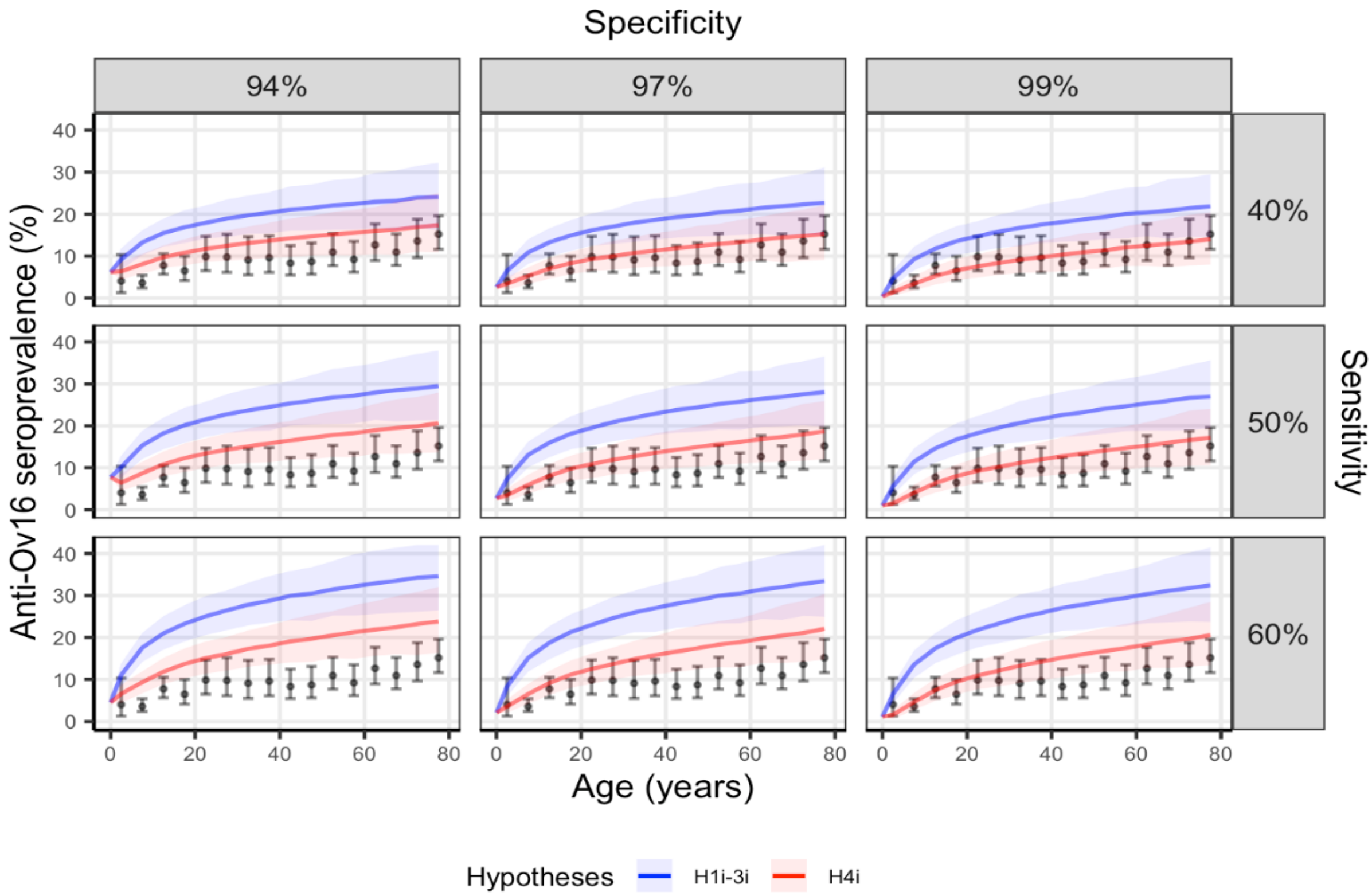

Supplementary Fig. 6. Observed and predicted anti-Ov16 IgG4 age-seroprevalence profiles for 4,257 individuals living in 58 ivermectin-naïve communities in Gabon[4], assuming no seroreversion. Solid coloured lines represent mean predicted age-seroprevalence profiles generated by EPIONCHO-IBM with EPIONCHO exposure (age- and sex-dependent exposure to blackfly bites)[1,17], calibrated to microfilarial prevalence estimates[4] (Fig. 1 of Main Text and Supplementary Fig. 1a) using an annual biting rate (ABR) = 76 bites/person/year and inter-individual exposure heterogeneity parameter $k_E = 0.2$. Predictions were generated under different seroconversion hypotheses (Fig. 2 of Main Text) assuming no seroreversion and adjusted for varying diagnostic performance of the SD BIOLINE Ov16 rapid diagnostic test (Ov16-RDT) using whole blood. Blue lines represent seroconversion elicited by pre-patent infection (hypotheses H1i–H3i, aggregated); red lines represent seroconversion elicited by near-patent/patent infection (hypothesis H4i). Ov16-RDT specificity is varied by column (94%, 97%, 99%), and sensitivity is varied by row (40%, 50%, 60%). Black circles show the observed seroprevalence estimates binned into 5-year age groups; the error bars are the associated 95% Wilson score confidence intervals. For each hypothesis and diagnostic performance, the model-predicted means and 95% uncertainty intervals (2.5th to 97.5th quantiles of the simulated mean; shaded areas) were calculated from 1,500 stochastic repeats. Source data originate from Atsame et al.[4]; the code to reproduce the figure with model output is in Ramani et al.[23]

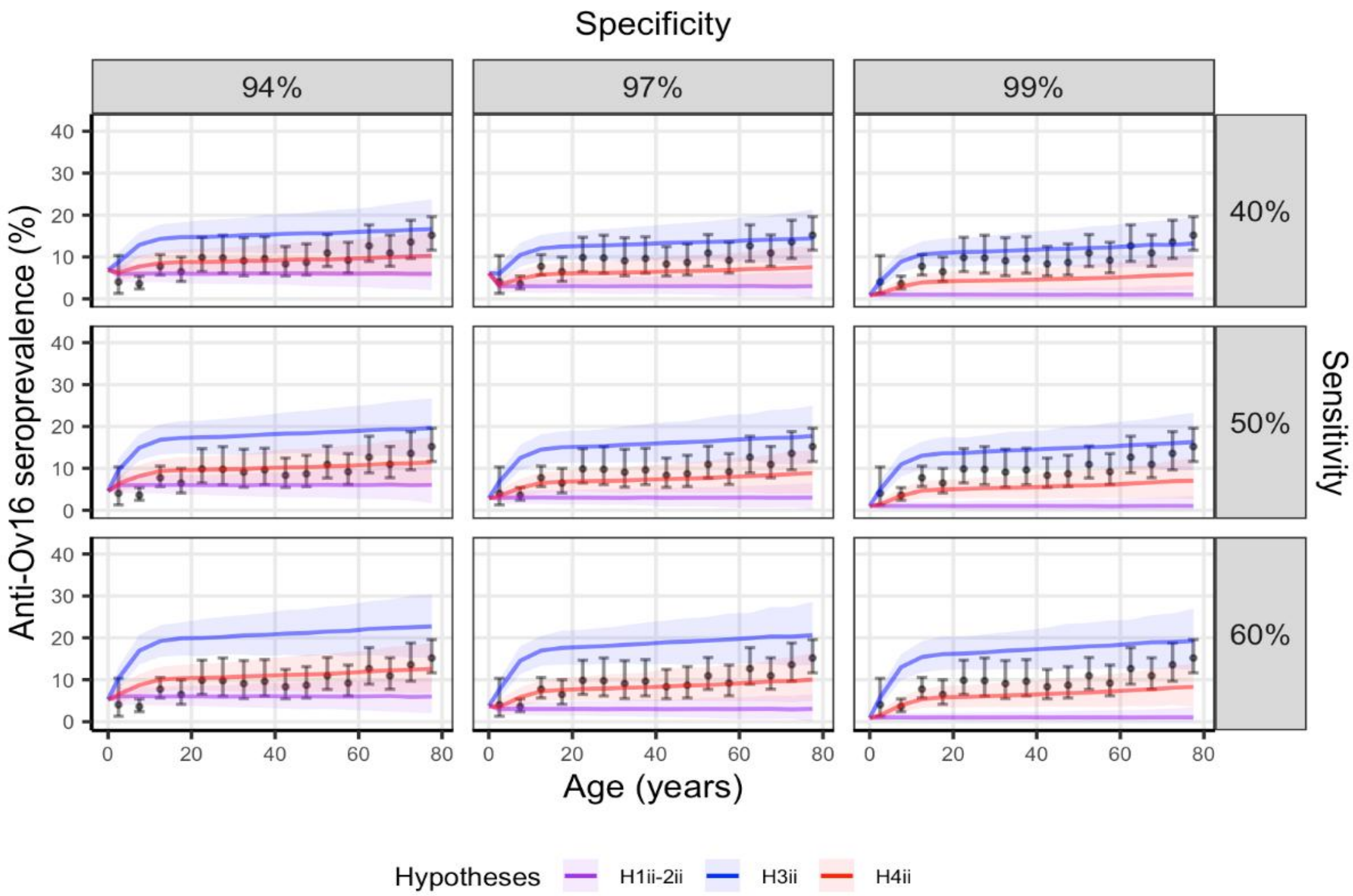

Supplementary Fig. 7. **Observed and predicted anti-Ov16 IgG4 age-seroprevalence profiles for 4,257 individuals living in 58 ivermectin-naïve communities in Gabon[4], assuming immediate seroreversion.** Solid coloured lines represent mean predicted age-seroprevalence profiles generated by EPIONCHO-IBM with EPIONCHO exposure (age- and sex-dependent exposure to blackfly bites)[1,17], calibrated to microfilarial prevalence estimates[4] (Fig. 1 of Main Text and Supplementary Fig. 1a) using an annual biting rate (ABR) = 76 bites/person/year and inter-individual exposure heterogeneity parameter k_E = 0.2. Predictions were generated under different seroconversion hypotheses (Fig. 2 of Main Text) assuming immediate seroreversion and adjusted for varying diagnostic performance of the SD BIOLINE Ov16 rapid diagnostic test (Ov16-RDT) using whole blood. Purple lines represent seroconversion elicited by L3 larvae or L4-L5 moult (hypotheses H1ii–H2ii, aggregated); blue lines represent seroconversion elicited by any established worm of either sex (hypothesis H3ii); red lines represent seroconversion elicited by near-patent/patent infection (hypothesis H4ii). Ov16-RDT specificity is varied by column (94%, 97%, 99%), and sensitivity is varied by row (40%, 50%, 60%). Black circles show the observed seroprevalence estimates binned into 5-year age groups; the error bars are the associated 95% Wilson score confidence intervals. For each hypothesis and diagnostic performance, the model-predicted means and 95% uncertainty intervals (2.5th to 97.5th quantiles of the simulated mean; shaded areas) were calculated from 1,500 stochastic repeats. Source data originate from Atsame et al.[4]; the code to reproduce the figure with model output is in Ramani et al.[23]

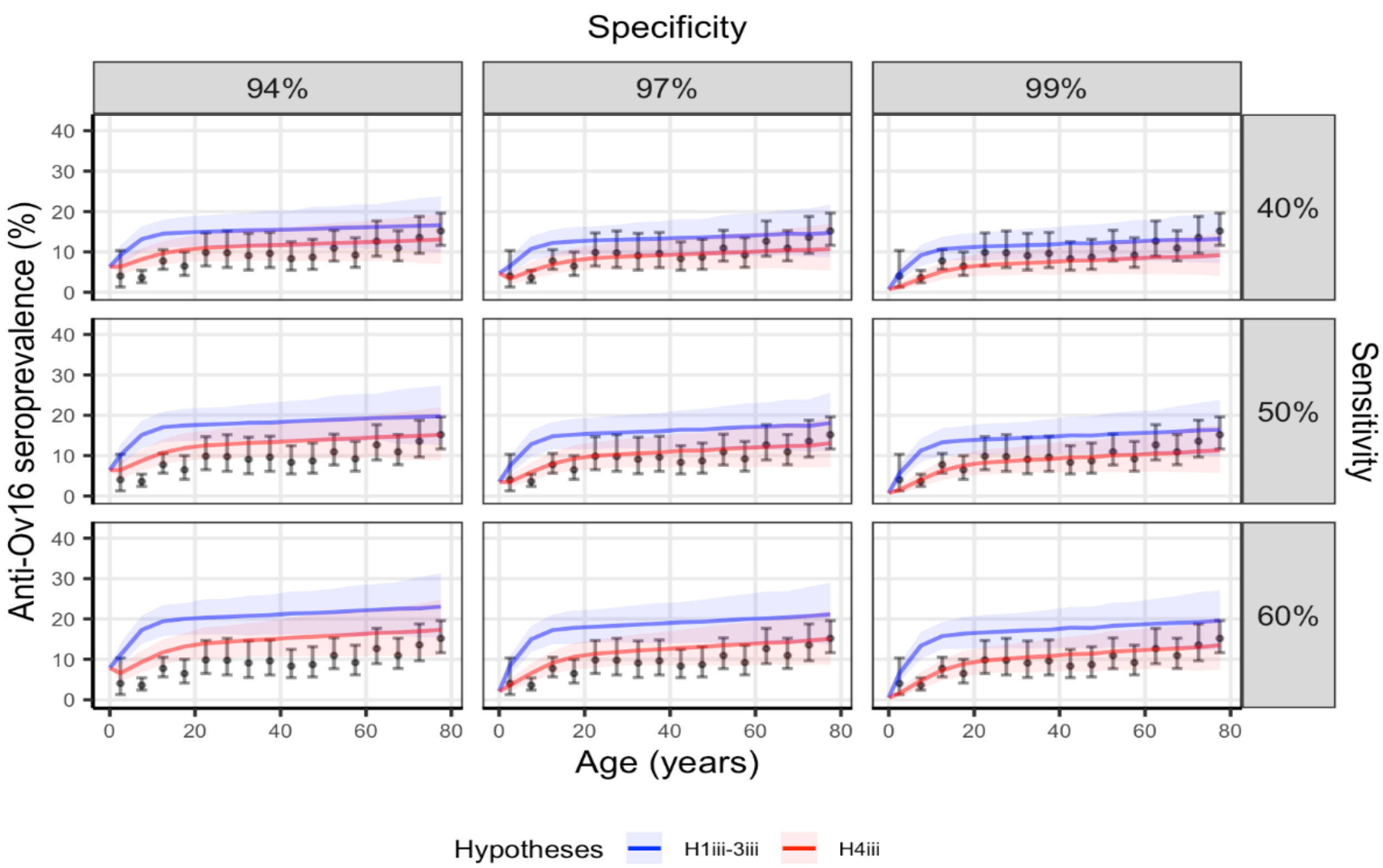

Supplementary Fig. 8. **Observed and predicted anti-Ov16 IgG4 age-seroprevalence profiles for 4,257 individuals living in 58 ivermectin-naïve communities in Gabon[4], assuming finite seroreversion.** Solid coloured lines represent mean predicted age-seroprevalence profiles generated by EPIONCHO-IBM with EPIONCHO exposure (age- and sex-dependent exposure to blackfly bites)[1,17], calibrated to microfilarial prevalence estimates[4] (Fig. 1 of Main Text and Supplementary Fig. 1a) using an annual biting rate (ABR) = 76 bites/person/year and inter-individual exposure heterogeneity parameter $k_E = 0.2$. Predictions were generated under different seroconversion hypotheses (Fig. 2 of Main Text) assuming finite seroreversion and adjusted for varying diagnostic performance of the SD BIOLINE Ov16 rapid diagnostic test (Ov16-RDT) using whole blood. Blue lines represent seroconversion elicited by pre-patent infection (hypotheses H1iii–H3iii, aggregated); red lines represent seroconversion elicited by near-patent/patent infection (hypothesis H4iii). Ov16-RDT specificity is varied by column (94%, 97%, 99%), and sensitivity is varied by row (40%, 50%, 60%). Black circles show the observed seroprevalence estimates binned into 5-year age groups; the error bars are the associated 95% Wilson score confidence intervals. For each hypothesis and diagnostic performance, the model-predicted means and 95% uncertainty intervals (2.5th to 97.5th quantiles of the simulated mean; shaded areas) were calculated from 1,500 stochastic repeats. Source data originate from Atsame et al.[4]; the code to reproduce the figure with model output is in Ramani et al.[23]

Supplementary Table 8. Categorizations and parameters defining the effectiveness of vector control (VC) and mass drug administration (MDA) of ivermectin in four prefectures of Togo for the period 1976-2015

Effectiveness categorization	Time period	Intervention	Frequency	Efficacy/coverage[a]	Systematic non-adherence[b]
Minimal	1976–2007[c]	VC	Continuous	60%	NA[d]
	1991–1995	MDA	Annual	50%	5%
	1996–2002	MDA	Annual	65%	5%
	2003–2015	MDA	Biannual	65%	5%
Reference	1976–2007[c]	VC	Continuous	75%	NA[d]
	1991–1995	MDA	Annual	50%	2.5%
	1996–2001	MDA	Annual	65%	2.5%
	2002	MDA	Annual	75%	2.5%
	2003–2015	MDA	Biannual	75%	2.5%
Enhanced	1976–2007[c]	VC	Continuous	90%	NA[d]
	1991–1995	MDA	Annual	65%	1%
	1996–2001	MDA	Annual	75%	1%
	2002	MDA	Annual	80%	1%
	2003–2015	MDA	Biannual	80%	1%

[a] For vector control (VC), efficacy is the percentage reduction in the blackfly annual biting rate during the intervention; for mass drug administration (MDA), coverage is the percentage of the total population that receive ivermectin at each round.

[b] Systematic non-adherence is the percentage of the eligible population (aged ≥ 5 years) who never participate in ivermectin MDA.

[c] Vector control stopped in 1993 for Ôti/Kpendjal prefectures[6].

[d] Systematic non-adherence is not applicable (NA) to vector control.

Supplementary Table 9. EPIONCHO-IBM-predicted and observed microfilarial prevalence estimates from 1,455 individuals living in four prefectures in Togo after 39 years of intervention[5]

Prefecture	Intervention effectiveness[a]	Predicted microfilarial prevalence in 2015 (95% uncertainty interval)	Observed microfilarial prevalence in 2015 (95% confidence interval)[b]
Ôti/Kpendjal	Mixed[c]	2.63% (0.000% – 9.23%)	2.47% (1.19% – 4.91%)
Kéran	Enhanced	11.8% (0.000% – 24.8%)	10.2% (7.92% – 13.1%)
Bassar	Minimal	3.25% (0.0707% – 8.03%)	3.38% (2.18% – 5.20%)
Prefectures combined	–	5.91% (0.789% – 11.8%)	5.70% (4.62% – 7.03%)

[a] Parameters defining intervention effectiveness are given in Supplementary Table 8.

[b] Mean and 95% (Wilson score) confidence interval calculated from data for three villages in Ôti/Kpendjal (Pancérys, Boutchakou and Koukoumbou); four villages in Kéran (Goulbi, Tchitchira, Koutougou Solla and Kpantiiyagou), and four villages in Bassar (Bawlesi, Mô-village, Katcha-Konkomba and Saboundi), from Komlan et al.[5]

[c] Each of the three villages in Ôti/Kpendjal were identified as having different intervention effectiveness, with simulations integrating this variability (see Supplementary Methods 2).

Supplementary Table 10. Estimates of sensitivity and specificity for the horseradish peroxidase (HRP) Ov16-ELISA

Age range (yr)	Sensitivity	Specificity	Reference diagnostic	Reference
≥10	81%	–	Skin-snip microscopy or nodule palpation	Ogunrinade et al.[11]
≥10	95%	–	Skin-snip microscopy or or nodule palpation	Ogunrinade et al.[11]
All ages	84%	94%	Skin-snip microscopy	Golden et al.[12]
All ages	60%	75%	Skin-snip microscopy or skin-snip PCR positive	Golden et al.[12]
<11	80%	97%	Laboratory-confirmed active infection	Golden et al.[13]
All ages	71.4%	71.1%	Skin-snip microscopy	Komlan et al.[5]
–	53%	>99%	Skin-snip microscopy	NTDSC, TFGH[10]
–	78%	>99%	Skin-snip microscopy	NTDSC, TFGH[10]
15 – 30 (IQR)[a]	83%	84.8%	Skin-snip microscopy/serostatus (concordant Ov16 ELISA & Ov16 RDT)	Hotterbeekx et al.[14]
All ages	82.5%	55.7%	Skin-snip microscopy	Djune-Yemeli et al.[15]

[a] IQR = Interquartile range.

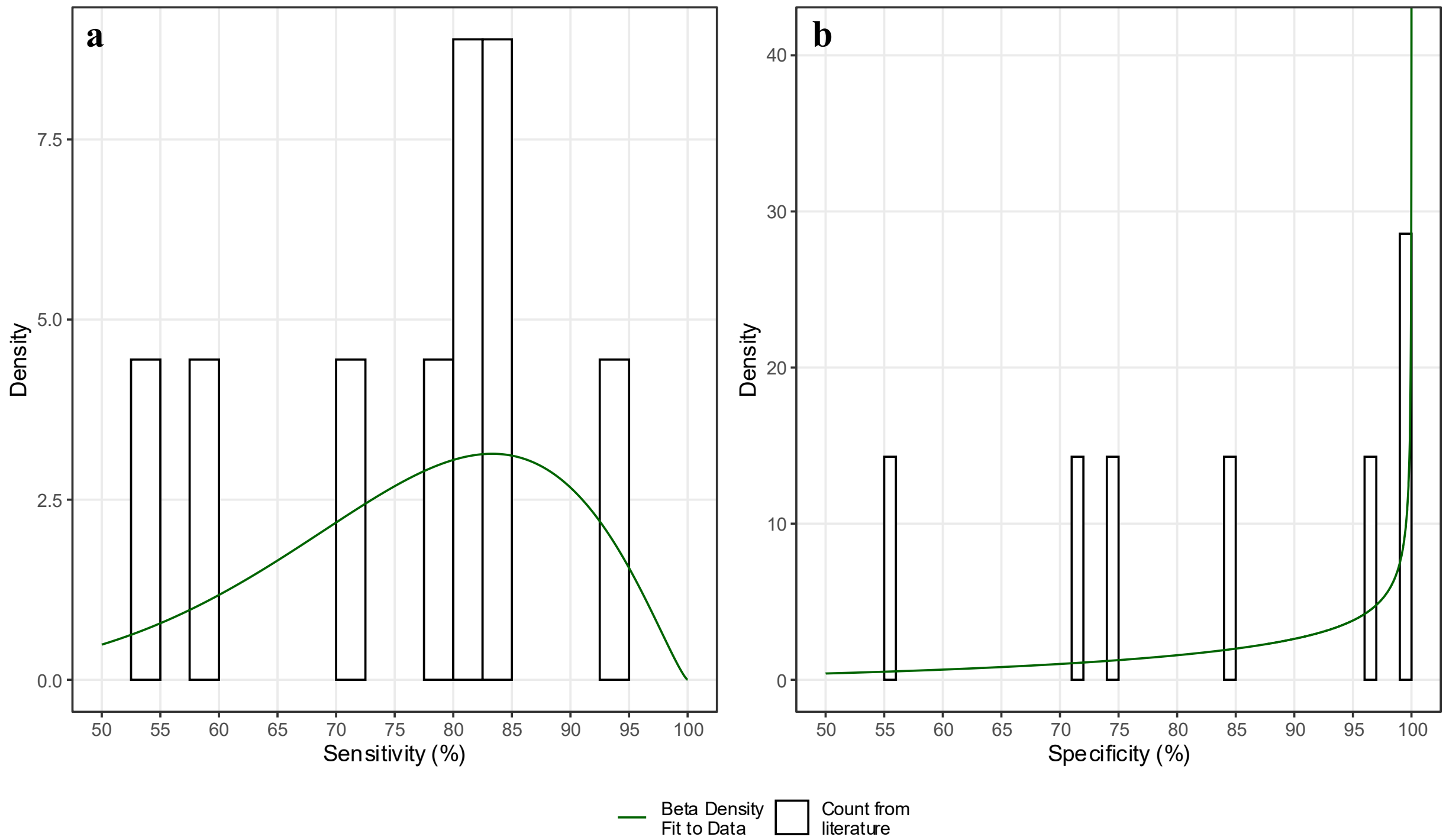

Supplementary Fig. 9. Distribution of sensitivity and specificity of the horseradish peroxidase Ov16-ELISA. White bars are reported values from the literature (Supplementary Table 10). Green lines are fitted beta distributions (Supplementary Methods 3). **a** Sensitivity, with shape parameters $\alpha = 7.40$, $\beta = 2.28$ (mean = 76.4%). **b** Specificity, with shape parameters $\alpha = 3.18$, $\beta = 0.635$ (mean = 83.2%).

Supplementary Table 11. Prefecture-specific predictive performance of EPIONCHO-IBM-projected versus observed anti-Ov16 IgG4 seroprevalence from 644 individuals living in four prefectures in Togo after 39 years of intervention[5] incorporating uncertainty in diagnostic performance of the horseradish peroxidase (HRP) Ov16-ELISA

Sensitivity[a]	Specificity[a]	Unnormalised mean squared error (MSE)[b] (95% Monte Carlo confidence interval)					
		Ôti/Kpendjal		Kéran		Bassar	
		Immediate seroreversion (H4ii)	Finite seroreversion (H4iii)	Immediate seroreversion (H4ii)	Finite seroreversion (H4iii)	Immediate seroreversion (H4ii)	Finite seroreversion (H4iii)
76.4% (46.8%−95.9%)	83.2% (38.6%−99.9%)	**329**[c,d] **(294−364)**[c,d]	723 (685−761)	572 (553−592)	**214**[d] **(206−222)**[d]	341 (322−360)	**227**[d] **(207−248)**[d]
		317[e] (284−349)[e]	652[e] (617−686)[e]	–	–	–	–

[a] Beta distribution mean (Supplementary Fig. 9) and 2.5th – 97.5th percentile.

[b] Mean squared error (MSE) is calculated as the arithmetic mean squared error (residual) and 95% Monte Carlo confidence interval from 1,500 model repeats (stochastic simulations) marginalised over 1,500 sensitivity/specificity replicates.

[c] Assuming 90% vector control efficacy for Ôti/Kpendjal when simulating enhanced interventions (see Supplementary Tables 8, 9 and Supplementary Methods 2).

[d] Best predictive performance (lowest MSE).

[e] Assuming 100% vector control efficacy for Ôti/Kpendjal when simulating enhanced interventions (see Supplementary Methods 2).

Supplementary Table 12. Prefecture-specific predictive performance of EPIONCHO-IBM-projected versus observed anti-Ov16 IgG4 seroprevalence from 644 individuals living in four prefectures in Togo after 39 years of intervention[5] assuming different sensitivity and specificity of the horseradish peroxidase (HRP) Ov16-ELISA

Sensitivity	Specificity	Unnormalised mean squared error (MSE)[a] (95% Monte Carlo confidence interval)					
		Ôti/Kpendjal		Kéran		Bassar	
		Immediate seroreversion (H4ii)	Finite seroreversion (H4iii)	Immediate seroreversion (H4ii)	Finite seroreversion (H4iii)	Immediate seroreversion (H4ii)	Finite seroreversion (H4iii)
70%	95%	38.6 (37.1–40)	212 (202–221)	905 (880–929)	243 (224–262)	405 (400–410)	59.8 (55.8–63.8)
80%		36.8 (35.3–38.2)	318 (304–331)	795 (769–821)	179 (161–197)	388 (383–394)	47.6 (44.1–51.1)
85%		36.4 (35–37.8)	379 (363–395)	742 (716–769)	167 (150–184)	376 (370–382)	47.6 (44.3–51)
70%	85%	**25.5**[b] **(23.9–27.1)**[b]	403 (390–415)	506 (490–521)	120 (109–131)	117 (115–120)	27.5 (25.8–29.2)
80%		30.8 (28.8–32.8)	552 (534–569)	423 (407–439)	101 (91–111)	108 (105–111)	54.4 (51.3–57.6)
85%		35.4 (33.1–37.8)	634 (614–654)	391 (375–408)	112 (102–122)	103 (100–106)	74.6 (70.5–78.8)
70%	70%	346 (341–352)	878 (863–893)	125 (119–131)	**39.7**[b] **(36.5–42.9)**[b]	**19.4**[b] **(18.4–20.4)**[b]	176 (171–181)
80%		368 (361–374)	1090 (1070–1110)	94.8 (89.1–101)	89.7 (85.7–93.8)	25.3 (23.9–26.7)	261 (254–269)
85%		374 (367–380)	1190 (1170–1220)	86.6 (81–92.3)	136 (130–141)	27.9 (26.4–29.4)	309 (301–317)

[a] Mean squared error (MSE) is calculated as the arithmetic mean squared error (residual) and 95% Monte Carlo confidence interval from 1,500 model repeats (stochastic simulations).

[b] Best predictive performance (lowest MSE) for each prefecture.

Supplementary Table 13. Aggregated (across all prefectures) predictive performance of EPIONCHO-IBM-projected versus observed anti-Ov16 IgG4 seroprevalence from 644 individuals living in four prefectures in Togo after 39 years of intervention[5] incorporating uncertainty in diagnostic performance of the horseradish peroxidase (HRP) Ov16-ELISA

**Sensitivity**[a]	**Specificity**[a]	**Aggregated unnormalised mean squared error (MSE)**[b] **(95% Monte Carlo confidence interval)**	
		**Immediate seroreversion (H4ii)**	**Finite seroreversion (H4iii)**
76.4% (46.8%–95.9%)	83.2% (38.6%–99.9%)	464[c] (439–489)	**415**[c,d] **(394–436)**[c,d c]
		468[e] (444–493)[e]	**389**[d,e] **(370–408)**[d,e]

[a] Beta distribution mean (Supplementary Fig. 9) and 2.5th – 97.5th percentile.

[b] Mean squared error (MSE) is calculated as the arithmetic mean squared error (residual) and 95% Monte Carlo confidence interval from 1,500 model repeats (stochastic simulations) marginalised over 1,500 sensitivity/specificity replicates weighted by the proportion of observations within each prefecture (Ôti/Kpendjal: 39.4%, Kéran: 55.2%, Bassar: 5.4%).

[c] Assuming 90% vector control efficacy for Ôti/Kpendjal when simulating enhanced interventions (see Supplementary Tables 8, 9 and Supplementary Methods 2).

[d] Best predictive performance (lowest MSE).

[e] Assuming 100% vector control efficacy for Ôti/Kpendjal when simulating enhanced interventions (see Supplementary Methods 2).

Supplementary Table 14. Aggregated (across all prefectures) predictive performance of EPIONCHO-IBM-projected versus observed anti-Ov16 IgG4 seroprevalence from 644 individuals living in four prefectures in Togo after 39 years of intervention[5] assuming different sensitivity and specificity of the horseradish peroxidase (HRP) Ov16-ELISA

Sensitivity	Specificity	Aggregated unnormalised Mean Squared Error (MSE)[a] (95% Monte Carlo confidence interval)	
		**Immediate seroreversion (H4ii)**	**Finite seroreversion (H4iii)**
70%	95%	536 (522–551)	221 (206–235)
80%		474 (459–489)	227 (211–242)
85%		444 (429–460)	244 (228–260)
70%	85%	296 (286–305)	226 (215–238)
80%		251 (242–261)	276 (263–288)
85%		236 (225–246)	316 (302–329)
70%	70%	**206**[b] **(201–212)**[b]	377 (369–385)
80%		**199**[b] **(193–204)**[b]	492 (481–502)
85%		**197**[b] **(191–202)**[b]	562 (549–575)

[a] Mean squared error (MSE) is calculated as the arithmetic mean squared error (residual) and 95% Monte Carlo confidence interval from 1,500 model repeats (stochastic simulations) weighted by the proportion of observations within each prefecture (Ôti/Kpendjal: 39.4%, Kéran: 55.2%, Bassar: 5.43%).

[b] Best predictive performance (lowest MSE).

Supplementary Table 15. Policy-Relevant Items for Reporting Models in Epidemiology of Neglected Tropical Diseases (PRIME-NTD) summary table

For the analyses presented, we adhered to the Five Principles of the Neglected Tropical Diseases (NTD) Modelling Consortium for good practice in policy-relevant NTD modelling[16], and briefly describe below the five tenets, how they were fulfilled, and where in the Main Text and/or Supplementary Material they can be found:

Principle	What has been done to satisfy the principle?	Where in the manuscript is this described?
**Stakeholder engagement**	Discussion with both modelling-focused collaborators and policy-focused collaborators on the underlying data and model results	Acknolwedgements
**Complete model documentation**	Link to documented code and vignettes have been provided, in addition to a description of additions to the model, and a reference to the full description of the model	Methods, Supplementary Methods, References, Code availability section
**Complete description of data used**	The data used consisted only of published data, and references have been provided to indicate where the data can be found and are fully described by the authors of the cited papers. Additionally, the data are described within the manuscript	Methods, Supplementary Methods, References, Data availability section
**Communicating uncertainty**	Uncertainty was considered throughout the study, including varying diagnostic sensitivity and specificity and sampling annual biting rates. Additionally, ninety-five percent (Wilson score, Monte Carlo) confidence intervals and uncertainty intervals were calculated and presented for the data, the projections and the mean squared errors as pertinent	Methods, Results, Supplementary Figures and Tables
**Testable model outcomes**	Model outcomes were first tested and compared to pre-intervention age-stratified seroprevalence data from Gabon. Then they were validated against seroprevalence data from 11 villages in Togo, accounting for intervention history	Results and Discussion, Supplementary Figures and Tables

Supplementary Methods

Supplementary Methods 1. Model calibration

In hypoendemic settings, microfilarial prevalence is highly sensitive to changes in annual biting rate (ABR, no. bites/person/year). Conversely, in meso- and hyperendemic settings a much broader range of ABRs is compatible with similar microfilarial prevalence values[17]. Due to the hypoendemic nature of the communities in Gabon, we aimed to find a single ABR—for values of the inter-individual exposure heterogeneity parameter, $k_E = 0.2$ or $k_E = 0.3$—that would minimise the discrepancy between the EPIONCHO-IBM predictions and the observed microfilarial prevalence. To achieve this, we undertook a two-stage calibration process, first identifying a sample of plausible ABR-k_E combinations and then fitting to the data to identify specific best-fit ABRs for each assumed value of k_E. For stage 1, we ran 100 simulations of EPIONCHO-IBM for a 100-year burn-in period and for a range of ABRs (for each k_E), comparing the mean microfilarial prevalence in those aged 5 years and older with that of the observed data[4]. When calculating the model-predicted mean prevalence, we sampled individuals in 5-year age groups to match the number of individuals in the respective age groups of the observed data (i.e., to weight correctly the prevalence by the sampled age demographic group). We selected and kept ABR-k_E combinations with a modelled mean microfilarial prevalence that was within the 95% (Wilson score) confidence interval (95% WCI) of the observed microfilarial prevalence (7.13%−8.75%) (Supplementary Fig. 1). For stage 2, we re-simulated EPIONCHO-IBM to endemic equilibrium 500 times for each of the selected ABR-k_E combinations and identified the ABR (for each k_E) that minimised the mean squared error (MSE) of the model predictions compared to the age-stratified (5-year groups) microfilarial prevalence data. The MSEs were calculated for each of the model runs by weighting each age group by the observed proportion of the population in that age-group (i.e., weighting more heavily more populated age groups). To account for potential bias introduced by the assumed age- and sex-dependent exposure function of the model, the calibration and hypothesis testing was replicated using the age- and sex-dependent exposure functions for both EPIONCHO-IBM (referred to as EPIONCHO exposure[1,3]) and ONCHOSIM (referred to as ONCHOSIM exposure[2,3]; see Supplementary Fig. 1). Supplementary Table 1 shows the range of

ABRs considered and selected (in stage 1), and the final (fitted, stage 2) ABR-k_E combinations for both age- and sex-dependent exposure functions.

In Togo, the pre-intervention baseline microfilarial prevalence was not known for all of the villages, so model-derived inferred estimates from Amaral et al.[6] were used when baseline prevalence was not available. Briefly, these values were inferred by identifying ABRs and parameters related to the effectiveness of interventions (vector control efficacy, treatment coverage and proportion of systematic non-adherence) that best captured microfilarial prevalence data collected longitudinally (by repeated cross-sectional surveys) throughout the intervention period. Intervention effectiveness was classified by Amaral et al.[6] as minimal, reference and enhanced defined, respectively, as a vector control efficacy of 60%, 75% and 90% (the percentage reduction in the ABR for the vector control period), a coverage of total population increasing to 65%, 75% and 80% (from 2002), and a systematic non-adherence (the proportion of individuals never taking treatment) of 5%, 2.5% and 1%. Treatment coverage (of total population) and frequency were fixed among all villages but varied temporally over the history of intervention[6] (Supplementary Table 8, Supplementary Methods 2).

All four villages in Kéran, were identified as having enhanced effectiveness (vector control efficacy 90%; total population coverage increasing from 65% in 1991-1995 to 80% from 2002, and systematic non-adherence 1%), with an inferred pre-intervention baseline microfilarial prevalence of 90%, excepting the village of Goulbi, which had an inferred prevalence of 70%. Hence, we used a prefecture-level aggregated baseline prevalence of 85% (the mean of three inferred prevalence values of 90% and one of 70%). In Bassar, all four villages were identified has having minimal intervention effectiveness (vector control efficacy 60%; total population coverage increasing from 50% in 1991-1995 to 65% from 2002, and systematic non-adherence 5%) and an inferred baseline microfilarial prevalence of 70%. Hence, we used a prefecture-level aggregated prevalence of 70%. The three villages in Ôti/Kpendjal were identified as having different intervention effectiveness (one each of minimal, reference and enhanced) but a consistent baseline microfilarial prevalence of 70%. Thus, we used a prefecture-level aggregate pre-intervention baseline prevalence of 70% and integrated variability in the intervention effectiveness when simulating interventions for the purposes of validation (Supplementary Methods 2).

Using the inferred prefecture-level aggregated pre-intervention microfilarial prevalence (Supplementary Table 2), we calibrated EPIONCHO-IBM by varying the ABR (using a $k_E = 0.3$[6], with 100 simulations per ABR) informed by previous work on the relationship between ABR and pre-intervention microfilarial prevalence[6,17] and selected ABRs that yielded predictions within a 1-percent-point range (e.g., 70%-71%) of the inferred prefecture-level pre-intervention microfilarial prevalence. Because of the highly hyperendemic nature of the villages within each prefecture (minimum pre-intervention baseline microfilarial prevalence of 70%), this approach yielded broad ranges of compatible ABRs (Supplementary Table 2). The resulting ABR distribution (Supplementary Fig. 3) was analysed using fitdistrplus[7] to estimate the parameters of a gamma distribution, which was then used to sample ABRs in subsequent model runs.

Supplementary Methods 2. Modelling interventions

The three prefectures in Togo have had a long history of intervention—including vector control from 1976 to 2007 (excepting Ôti/Kpendjal, where vector control stopped in 1993) and MDA from 1991—before the seroprevalence data were collected in 2015. We followed Amaral et al.[6] in simulating the history of MDA and vector control. For MDA, the frequency and coverage were divided into temporal segments reflecting the evolution of the programme, which shifted from the initial goal of control to elimination. This consisted of early MDA delivered by mobile teams (1991-1995), increased coverage associated with the switch to community directed treatment with ivermectin (CDTI) (1996-2001) and the switch to higher coverage and biannual MDA (2002-2015 and 2003-2015, respectively). For each segment, we assumed a coverage (of the total population) as used by Amaral et al.[6], which was informed by national programme data[5]. For vector control, we also followed Amaral et al.[6] in defining the period of intervention, from 1976-2007 that applied to Special Intervention Zones (excepting Ôti/Kpendjal where vector control ceased in 1993[6]), and defined vector control efficacy as the reduction in ABR for the period of aerial larviciding of vector breeding sites. Variation in the effectiveness of intervention in each prefecture was simulated using parameters defining the efficacy of vector control, the total population ivermectin treatment coverage, and the proportion of systematic non-adherence (categorised as minimal, reference and enhanced; see Supplementary Table 8 and Supplementary Methods 1).

The microfilarial prevalence and anti-Ov16 IgG4 seroprevalence in 2015 were predicted by first using EPIONCHO-IBM to simulate to endemic equilibrium, sampling ABRs from the range that reflected a prefecture's pre-intervention baseline microfilarial prevalence (Supplementary Methods 1), and then simulating the history of intervention—assuming a minimal, reference or enhanced effectiveness—using 1,500 stochastic repeats. For Ôti/Kpendjal, we integrated variability in the identified effectiveness categorisation of each village (Supplementary Methods 1) by combining 500 stochastic repeats of each of the minimal, reference and enhanced categorisations. We assumed the best fitting seroconversion-seroreversion hypotheses (from the prior analysis of the Gabon data) and recorded microfilarial prevalence and anti-Ov16 IgG4 seroprevalence predictions at the start of 2015 to align with the year when the serological and parasitological data were collected by Komlan et al.[5] (microfilarial prevalence predictions are shown in Supplementary Table 9). Aggregated prevalence and seroprevalence were calculated using a sampled population from each prefecture-specific projection that matched the proportion of individuals in the study from each prefecture (i.e., weighted by the sample size from each prefecture; 324/1455 = 22.3% for microfilarial prevalence and 254/644 = 39.4% for anti-Ov16 IgG4 seroprevalence in Ôti/Kpendjal; 539/1455 = 37.0% and 355/644 = 55.1% in Kéran; 592/1455 = 40.7% and 35/644 = 5.43% in Bassar).

Supplementary Methods 3. Sensitivity and specificity estimates

Ov16 Rapid Diagnostic Test. We conducted a literature search to identify studies reporting estimates of the sensitivity and specificity of the SD BIOLINE Ov16 rapid diagnostic test (Ov16-RDT) used in Gabon, and the horseradish peroxidase (HRP) Ov16-ELISA used in Togo. For the SD BIOLINE Ov16-RDT, we conducted a search on EMBASE using the following terms:

1. ("ov16" or "Ov16" or "IgG4").mp. [mp=title, abstract, heading word, drug trade name, original title, device manufacturer, drug manufacturer, device trade name, keyword heading word, floating subheading word, candidate term word]
2. ("RDT" or "Rapid Diagnostic Test").mp. [mp=title, abstract, heading word, drug trade name, original title, device manufacturer, drug manufacturer, device trade name, keyword heading word, floating subheading word, candidate term word]

3. ("SD" or "Bioline" or "SD Bioline").mp. [mp=title, abstract, heading word, drug trade name, original title, device manufacturer, drug manufacturer, device trade name, keyword heading word, floating subheading word, candidate term word]

4. 1 and 2 and 3

We filtered the search results to identify studies using SD BIOLINE Ov16-RDTs on whole blood and reporting diagnostic characteristics (sensitivity and specificity). Additionally, studies discussing the diagnostic characteristics of Ov16-RDTs were also considered, along with the diagnostic performance of the RDT reported from Gabon dataset[4]. Of the 16 studies identified, two estimated the sensitivity and specificity of the RDT using whole blood against skin snips, with sensitivities of 51% and 60% and specificities of 95% and 94%, respectively (Supplementary Table 3). Two studies used Ov16-ELISA as the reference test, estimating sensitivities of 29% and 40%, and specificities of 98.9% and 99.9% respectively (Supplementary Table 3). We did not consider the diagnostic sensitivity and specificity reported on the SD BIOLINE manufacturer instructions as reported by Dieye et al.[18], because these values were obtained in a laboratory setting. In the field, and especially in low prevalence areas, the performance of RDTs[10] (and whole-blood RDTs in particular[19]) is likely to be substantially lower. Indeed, in the Ov16 Technical Meeting (held in May 2016)[10], it was also noted that in Gabon, the sensitivity of RDTs was 78% in meso- to hyperendemic areas, but only 46% in hypoendemic areas (which matches the setting of our data).

Ov16 Horse Radish Peroxidase (HRP) ELISA. For the Ov16 HRP ELISA, we conducted a search on EMBASE using the following terms:

1. ("onchocerciasis" or "oncho*").mp. [mp=title, abstract, heading word, drug trade name, original title, device manufacturer, drug manufacturer, device trade name, keyword heading word, floating subheading word, candidate term word]

2. (("sensitivity" and "specificity") or ("sens" and "spec")).mp. [mp=title, abstract, heading word, drug trade name, original title, device manufacturer, drug manufacturer, device trade name, keyword heading word, floating subheading word, candidate term word]

3. ("ELISA" or "Enzyme Linked Immunosorbent Assay").mp. [mp=title, abstract, heading word, drug trade name, original title, device manufacturer, drug manufacturer, device trade name, keyword heading word, floating subheading word, candidate term word]
4. ("Ov16" or "IgG4").mp. [mp=title, abstract, heading word, drug trade name, original title, device manufacturer, drug manufacturer, device trade name, keyword heading word, floating subheading word, candidate term word]
5. 1 and 2 and 3 and 4

We filtered the search results to identify studies using HRP as the enzyme conjugate and reported the sensitivity and specificity or presented data permitting their calculation. We focused on studies using the HRP Ov16 ELISA as being the most similar to that used by Komlan et al.[5] for their study in Togo. The other main enzyme conjugate used in Ov16 ELISA assays is alkaline phosphatase (AP). There are varying reasons to use one or the other; however, with Thermo Scientific Kits, the ELISA substrates that can be used with HRP enzymes have lower detection limits (picogram range) than those with AP (nanogram range)[20,21]. This typically results in higher sensitivities[22] (at the compromise of lower specificity) for the HRP ELISA. The reported sensitivity and specificity values found by the search are shown in Supplementary Table 10.

We fitted beta distributions to the sensitivity and specificity estimates for each diagnostic (SD BIOLINE Ov16-RDT on whole blood and HRP Ov16-ELISA) using the mean and variance to estimate the shape parameters, α and β (i.e., moment estimators). Supplementary Fig. 4 and 9 show the reported estimates and fitted beta distributions for the RDT and HRP ELISA respectively. We used these parameterised beta distributions to sample diagnostic parameters (sensitivity/specificity) for each of our EPIONCHO-IBM simulations to integrate uncertainty in diagnostic performance into the resulting seroprevalence projections

In addition to integrating diagnostic performance uncertainty—and to make explicit the effect of changes in sensitivity/specificity on our inference—we also ran sensitivity analysis using point estimates of the sensitivity and specificity for each diagnostic within the range of values reported from the literature. For the SD BIOLINE Ov16-RDT, we used sensitivities of 40%, 50%, and 60%

and specificities of 95%, 97%, and 99%. For the HRP Ov16-ELISA, we used sensitivities of 70%, 80%, and 85%, and specificities of 70%, 85%, and 95%.

Supplementary References

1. Filipe, J. A. N., Boussinesq, M., Renz, A., Collins, R. C., Vivas-Martinez, S., Grillet, M. E., Little, M. P. & Basáñez, M.-G. Human infection patterns and heterogeneous exposure in river blindness. *Proc. Natl. Acad. Sci. U. S. A* **102**, 15265−15270 (2005).
2. Lont, Y. L., Coffeng, L. E., de Vlas, S. J., Golden, A., de Los Santos, T., Domingo, G. J. & Stolk, W. A. Modelling anti-Ov16 IgG4 antibody prevalence as an indicator for evaluation and decision making in onchocerciasis elimination programmes. *PLoS Negl. Trop. Dis.* **11**, e0005314 (2017).
3. Hamley, J. I. D., Walker, M., Coffeng, L. E., Milton, P., de Vlas, S. J., Stolk, W. A. & Basáñez, M.-G. Structural uncertainty in onchocerciasis transmission models influences the estimation of elimination thresholds and selection of age groups for seromonitoring. J. Infect. Dis. **221(Suppl 5)**, S510−S518 (2020).
4. Atsame, J., Stapley, J. N., Ramani, A., Mourou, R., Ntsame, E., Efame, E., Angue, O.-N., Obiang, J.-L., Pilotte, N., Gass, K. & Basáñez, M.-G. Comparison of diagnostic tools to assess the feasibility of programmatic use of rapid diagnostic tests for onchocerciasis: a dataset from Gabon. *Data Brief* **57,** 110901 (2024).
5. Komlan, K., Vossberg, P. S., Gantin, R. G., Solim, T., Korbmacher, F., Banla, M., Padjoudoum, K., Karabou, P., Köhler, C. & Soboslay, P. T. *Onchocerca volvulus* infection and serological prevalence, ocular onchocerciasis and parasite transmission in northern and central Togo after decades of *Simulium damnosum* s.l. vector control and mass drug administration of ivermectin. *PLoS Negl. Trop. Dis.* **12,** e0006312 (2018).
6. Amaral, L.-J., Bronzan, R. N., Seim, A., Milord, M.-D., Padjoudoum, K., Telou, I. G., Agoro, S., Datagni, M., Gnossike, P., Hamley, J. I. D., Walker, M. & Basáñez, M.-G. Reaching elimination of onchocerciasis transmission with long-term vector control and ivermectin treatment in Togo. *Nat. Commun.* **17,** 779 (2025).
7. Delignette-Muller, M. L. & Dutang, C. fitdistrplus: An R package for fitting distributions. *J. Stat. Softw.* **64**, 1–34 (2015).
8. Nikièma A. S., Koala, L., Unnasch, T. R., Diendéré, J., Compaoré, J., Ouédraogo, M. W., Kafando, C. M., Bakajika, D., Bougouma, C., Faye, B., Traoré, S. & Dabiré, R. K. Field sensitivity and specificity of the SD BIOLINE onchocerciasis IgG4 Rapid Diagnostic Test in children <10 years old from endemic areas in Burkina Faso. *Parasite Epidemiol. Control* **25**, e00352 (2024).

9. Ogawa, G. M., Golden, A., Bhingarde, J. A., Nshala, A., Liu, E., Newsam, A., Wiegand, R., Cantey, P. & Cama, V. Improved performance of a serology rapid diagnostic test for onchocerciasis by using dried blood spots. Abstract 530, 68th Annual Meeting of the American Society of Tropical Medicine and Hygiene, Maryland, USA, November 20–24, 2019. *Am. J. Trop. Med. Hyg.* **101 (5 Suppl),** 162 (2019).

10. Neglected Tropical Diseases Support Center, Taskforce for Global Health. Decatur, GA, USA, May 2–3, 2016. Ov-16 Meeting Notes. https://www.cor-ntd.org/resources/ov-16-meeting-notes (2016).

11. Ogunrinade A. F., Chandrashekar R., Eberhard M. L. & Weil G. J. Preliminary evaluation of recombinant *Onchocerca volvulus* antigens for serodiagnosis of onchocerciasis. *J. Clin. Microbiol.* **31**, 1741–1745 (1993).

12. Golden, A. Steel, C., Yokobe, L., Jackson, E., Barney, R., Kubofcik, J., Peck, R., Unnasch, T. R., Nutman, T. B., de Los Santos, T. & Domingo, G. J. Extended result reading window in lateral flow tests detecting exposure to *Onchocerca volvulus*: a new technology to improve epidemiological surveillance tools. *PLoS One.* **8**, e69231 (2013).

13. Golden, A., Faulx, D., Kalnoky, M., Stevens, E., Yokobe, L., Peck, R., Karabou, P., Banla, M., Rao, R., Adade, K., Gantin, R. G., Komlan, K., Soboslay, P. T., de Los Santos, T. & Domingo, G. J. Analysis of age-dependent trends in Ov16 IgG4 seroprevalence to onchocerciasis. *Parasit. Vectors* **9,** 338 (2016).

14. Hotterbeekx, A., Perneel, J., Mandro, M., Abhafule, G., Siewe Fodjo, J. N., Dusabimana, A., Abrams, S., Kumar-Singh, S. & Colebunders, R. Comparison of diagnostic tests for *Onchocerca volvulus* in the Democratic Republic of Congo. *Pathogens* **9**, 435 (2020).

15. Djune-Yemeli, L., Domché, A., Nana-Djeunga, H. C., Donfo-Azafack, C., Lenou-Nanga, C. G., Masumbe-Netongo, P. & Kamgno, J. Relationship between skin snip and Ov16 ELISA: two diagnostic tools for onchocerciasis in a focus in Cameroon after two decades of ivermectin-based preventive chemotherapy. *PLoS Negl. Trop. Dis.* **16**, e0010380 (2022).

16. Behrend, M. R., Basáñez, M.-G., Hamley, J. I. D., Porco, T. C., Stolk, W. A, Walker M, de Vlas, S. J. & NTD Modelling Consortium. Modelling for policy: the five principles of the Neglected Tropical Diseases Modelling Consortium. *PLoS Negl. Trop. Dis.* **14,** e0008033 (2020).

17. Hamley, J. I. D., Milton, P., Walker, M. & Basáñez, M.-G. Modelling exposure heterogeneity and density dependence in onchocerciasis using a novel individual-based transmission model, EPIONCHO-IBM: implications for elimination and data needs. *PLoS Negl. Trop. Dis.* **13,** e0007557 (2019).

18. Dieye, Y., Storey, H. L., Barrett, K. L., Gerth-Guyette, E., Di Giorgio, L., Golden, A., Faulx, D., Kalnoky, M., Ndiaye, M. K. N., Sy, N., Mané, M., Faye, B., Sarr, M., Dioukhane, E. M., Peck, R. B., Guinot, P. & de Los Santos, T. Feasibility of utilizing the SD BIOLINE

Onchocerciasis IgG4 rapid test in onchocerciasis surveillance in Senegal. *PLoS Negl. Trop. Dis.* **11**, e0005884 (2017).

19. Bennuru S., Oduro-Boateng, G., Osigwe, C., Del Valle, P., Golden, A., Ogawa, G. M., Cama, V., Lustigman, S. & Nutman, T. B. Integrating multiple biomarkers to increase sensitivity for the detection of *Onchocerca volvulus* infection. *J. Infect. Dis.* **221**, 1805–1815 (2019).
20. Thermo Fisher Scientific. ELISA Enzyme Substrates. https://www.thermofisher.com/uk/en/home/life-science/antibodies/immunoassays/elisa-kits/elisa-enzyme-substrates.html (2026).
21. Thermo Fisher Scientific. Guide to enzyme substrates for ELISA. https://assets.thermofisher.com/TFS-Assets/LSG/Application-Notes/TR0033-Substrates-ELISA.pdf (2011).
22. Beyzavi, K., Hampton, S., Kwasowski, P., Fickling, S., Marks, V. & Clift, R. Comparison of horseradish peroxidase and alkaline phosphatase-labelled antibodies in enzyme immunoassays. *Ann. Clin. Biochem*. **24**, 145–152 (1987).
23. Ramani, A., Dixon, M. & Stapley, J. mrc-ide/EPIONCHO.IBM: analysis for modelling anti-Ov16 seroprevalence for the control and elimination of onchocerciasis. *Zenodo.* https://doi.org/10.5281/zenodo.21244449 (2026).
