# Supplementary material for: Modelling anti-Ov16 seroprevalence for the control and elimination of onchocerciasis
Source: Nat Commun. 2026 Aug 19;17:9924. doi: 10.1038/s41467-026-76562-9 (PMC13582928; doi:10.1038/s41467-026-76562-9)
Supplement: Supplementary file 2 [file 41467_2026_76562_MOESM2_ESM.pdf]

nature portfolio

Peer Review File

Modelling anti-Ov16 seroprevalence for the control and elimination of onchocerciasis

Corresponding Author: Professor Maria-Gloria Basáñez

This file contains all reviewer reports in order by version, followed by all author rebuttals in order by version.

Attachments originally included by the reviewers as part of their assessment can be found at the end of this file.

Version 0:

Reviewer comments:

Reviewer #1

(Remarks to the Author)
• What are the noteworthy results?
This paper presents a model calibration and validation that allows testing of variable combinations of seroconversion and reversion hypotheses. The authors seek to identify thresholds which are indicative of unstable transmission and/or pathway to elimination to identify the measurable thresholds to inform stopping MDA thresholds that are feasible within diagnostic performance of Ov16 serological tests. The article is well written, and overall the article would benefit from providing more substantial linkage between the modelled work and setting thresholds.
• Will the work be of significance to the field and related fields? How does it compare to the established literature? If the work is not original, please provide relevant references.
o Yes, the work of this type of modeling helps to inform stopping decisions for MDA and guidelines for elimination verification. The authors acknowledge that serological thresholds in modeling are highly dependent on underlying assumptions.
o This model allows discrepancy between skin snip results and serological results.
• Does the work support the conclusions and claims, or is additional evidence needed?
o Yes, the model calibrated using mf prevalence fits the observed data serological evidence though both tested models generally fit.
o Different diagnostic performances were modeled and showed better or poorer fit by MSE– the authors may want to clarify more the anticipated reasons for this and prevent gleaning diagnostic performance from the best fit data.
• Are there any flaws in the data analysis, interpretation and conclusions? Do these prohibit publication or require revision?
o Overall the discussion is logically presented and the analysis and code are made available. There is no major flaw that should prevent publication. Please consider the following points to enhance discussion and to render the content more accessible to non-modelers.
o It would be helpful to include some discussion of whether there could be assumed Ov16-negative due to reasons other than diagnostic failure. For example, it has been observed that approximately 20% of skin snip positive elicit no detectable anti-Ov16 IgG4. This could be due to deficiency in IgG4, or lack of immune response to Ov16, in addition to a technical sensitivity deficiency in the ELISA or RDT.
o Please clarify why why H4 (i) chosen over H4(ii). Although the minimum values at some sensitivity and specificities were in seroreversion hypothesis (i), across different seroconversion and different sensitivity and specificity values, it appeared that (ii) had more often a better MSE.
o Is there any diagnostic test bias introduced by seroreversion hypothesis – for example, if the reversion hypothesis more closely matches data, but in reality there is poorer sensitivity of the diagnostic test.
o Is there any accommodation for differential performance of the diagnostic depending on the prevalence and treatment history – some concerns about the serological performance having poorer sensitivity in populations who have been under treatment for longer or are hypoendemic.
o Given the wide confidence intervals for higher ages, could the validation be focused on younger age groups – those most important for establishing elimination?
• Is the methodology sound? Does the work meet the expected standards in your field?
o Yes, the authors are attentive to best practice and standards set by modelling consortia.
o It would be helpful to provide some context about the key finding that the near-patent/patent seroconversion may be driving

the superior performance. Some combination with all seroconversion (especially with ii seroreversion) hypotheses produced low MSE instances. Can different conversion/reversion combinations produce similar results though some offset?
• Is there enough detail provided in the methods for the work to be reproduced?
o Yes, the authors have provided code for this model and supplemental information.

(Remarks on code availability)
I do not have the required expertise to check the code. The presence of the code on public website was confirmed.

Reviewer #2

(Remarks to the Author)
The manuscript is well written and can be published once the authors have attended to the issues raised in the attached file.

(Remarks on code availability)
The review is provided in the attached file.

Version 1:

Reviewer comments:

Reviewer #2

(Remarks to the Author)
The manuscript has improved substantially, and I commend the authors for thoughtfully addressing the reviewers' comments. The revisions have strengthened the clarity, rigor, and overall quality of the work. I therefore recommend the manuscript for publication.

(Remarks on code availability)
The authors provided all necessary code and documentation to support reproducibility. The repository includes a clear README with sufficient instructions for installation and execution, and the analyses were reproducible using the supplied materials. The code is well organized and represents a usable and valuable resource for the community.

Reviewer #3

(Remarks to the Author)
I reviewed the authors' responses to the Reviewer's 1 comments. According to the letter detailing the responses to the reviewers' comments, I noted that Reviewer 1 had made 15 points, consisting of 7 comments and 8 questions. The authors have responded to all of these.
Regarding the comments, the authors thanked reviewer 1 for the comments and appreciated his comment made for his interest in their study.
Regarding the questions, the authors also answered all the questions (1.2., 1.6, 1.8, 1.9, 1.10, 1.11, 1.12, 1.14). For each question, they provided a satisfactory response. They also incorporated the changes made to address the various questions into the manuscript, taking a great care to highlight them.
In conclusion, the authors have satisfactorily addressed the concerns raised by the Reviewer 1.

(Remarks on code availability)

Open Access This Peer Review File is licensed under a Creative Commons Attribution 4.0 International License, which permits use, sharing, adaptation, distribution and reproduction in any medium or format, as long as you give appropriate credit to the original author(s) and the source, provide a link to the Creative Commons license, and indicate if changes were made.
In cases where reviewers are anonymous, credit should be given to 'Anonymous Referee' and the source.
The images or other third party material in this Peer Review File are included in the article's Creative Commons license, unless indicated otherwise in a credit line to the material. If material is not included in the article's Creative Commons license and your intended use is not permitted by statutory regulation or exceeds the permitted use, you will need to obtain permission directly from the copyright holder.
To view a copy of this license, visit https://creativecommons.org/licenses/by/4.0/

REVIEWERS' COMMENTS

Reviewer #1 (Remarks to the Author):

• What are the noteworthy results?

1.1 This paper presents a model calibration and validation that allows testing of variable combinations of seroconversion and reversion hypotheses. The authors seek to identify thresholds which are indicative of unstable transmission and/or pathway to elimination to identify the measurable thresholds to inform stopping MDA thresholds that are feasible within diagnostic performance of Ov16 serological tests. The article is well written.

Reply by Authors to 1.1. We thank the Reviewer for this appreciative comment.

1.2 Overall the article would benefit from providing more substantial linkage between the modelled work and setting thresholds.

Reply by Authors to 1.2. We thank the Reviewer for highlighting this important point. We agree that the use of models to define serological thresholds is of critical importance to onchocerciasis elimination programmes, including for initiating MDA in previously untreated foci, for stopping MDA and verifying elimination and for responding to serological signals in post-treatment/post-elimination surveillance periods. We have emphasised these applications throughout the revised manuscript. For example, in the Discussion:

> "This represents a major advance in the use of transmission models to inform serological thresholds for key public health decisions such as initiating or stopping MDA, and informing the elimination of onchocerciasis transmission during the PTS and PES periods"

> "As onchocerciasis elimination programmes continue transitioning away from using skin-snip microscopy to (less invasive) serological surveillance, it is essential that transmission models advance in parallel to remain useful for public health decision-making….This is a major step forward for onchocerciasis transmission dynamics modelling, providing a critical tool to support programmatic decisions on initiating MDA, stopping treatment safely, determining elimination, and responding to serological signals during PTS and PES."

In addition, to make the linkage explicit, we have added a sentence in the Discussion clarifying that the principal contribution of this work is to provide a mechanistic mapping from transmission status/intervention history to age-specific Ov16 seroprevalence (accounting for seroreversion and imperfect test performance), which is essential for subsequent threshold-defining modelling work:

> "Specifically, by embedding a biologically-motivated seroconversion–seroreversion process within a validated transmission model, our framework provides a quantitative mapping from underlying transmission status and intervention history to the expected age-specific distribution of Ov16 seroprevalence (under imperfect test performance), which is essential to evaluate and refine operational start-/stop-MDA and PTS/PES serological thresholds."

• Will the work be of significance to the field and related fields? How does it compare to the established literature? If the work is not original, please provide relevant references.

1.3 The work of this type of modeling helps to inform stopping decisions for MDA and guidelines for elimination verification. The authors acknowledge that serological thresholds in modeling are highly dependent on underlying assumptions.

Reply by Authors to 1.3. We thank the Reviewer for recognising the significance of our work towards informing stopping MDA decisions, which was indeed the original motivation of our work. We are also grateful to the Reviewer for acknowledging our attention to the importance of modelling assumptions, which we have subjected to extensive sensitivity analysis to strengthen the robustness of our conclusions.

1.4 This model allows discrepancy between skin snip results and serological results.

Reply by Authors to 1.4. We appreciate that the Reviewer recognises our efforts to model anti-Ov16 seropositivity in the absence of detectable skin-snip positivity.

• Does the work support the conclusions and claims, or is additional evidence needed?

1.5 The model calibrated using mf prevalence fits the observed data serological evidence though both tested models generally fit.

Reply by Authors to 1.5. We appreciate that the Reviewer acknowledges the validity of our calibration and the visual comparison of model outputs with the anti-Ov16 serological data. However, we would like to stress that our seroconversion-seroreversion hypotheses are also compared quantitatively using mean squared errors which yield clear differences in the goodness-of-fit of the various hypotheses.

1.6 Different diagnostic performances were modeled and showed better or poorer fit by MSE– the authors may want to clarify more the anticipated reasons for this and prevent gleaning diagnostic performance from the best fit data.

Reply by Authors to 1.6. We are grateful for the opportunity to clarify this point. We use different assumptions on the diagnostic performance of anti-Ov16 serological tests to evaluate the robustness of our conclusions on the seroconversion–seroreversion hypotheses. We do not infer diagnostic performance from model fit; instead, we treat diagnostic performance as uncertain and integrate this uncertainty to strengthen the robustness of our conclusions. Importantly, a lower MSE under a given diagnostic assumption should not be interpreted as evidence that this assumption is "more correct": diagnostic sensitivity/specificity are not identifiable from these seroprevalence data alone because they trade off against seroreversion dynamics (and other unobserved processes). Differences in MSE arise because alternative combinations of effective sensitivity and seroreversion timing can generate similar age-specific seroprevalence patterns, so some combinations match a given dataset marginally better even though multiple explanations remain plausible. Estimating diagnostic performance would require independent, paired diagnostic data and is outside the scope of this analysis. Indeed, we are unable to reach strong conclusions on the duration of immunological memory (time to seroreversion) because of the confounding effect of diagnostic uncertainty. This is expressly acknowledged in the Discussion section of the manuscript and is supported by figures and tables in the Supplementary Material:

> "In our analysis of the Gabon data, we were unable to distinguish between immediate seroreversion (i.e., seroreversion occurring after the loss of all mating worm pairs) and finite seroreversion (i.e., occurring after clearance of all parasite stages). This is not unexpected as seroreversion and diagnostic sensitivity are confounded; higher sensitivity can compensate, to some extent, for declining

antibody responses. This can be seen in Supplementary Figures 6–8 and Supplementary Tables 6–7, where, when using point diagnostic performance adjustments, simulations assuming no seroreversion with poorer diagnostic performance provide the best fit to the Gabon data, while simulations using instant or finite seroreversion provided the best fit as diagnostic performance increased."

"We emphasize that differences in MSEs across diagnostic assumptions are used only for robustness assessment and do not constitute evidence about the true diagnostic performance, which is not identifiable from these data alone."

• Are there any flaws in the data analysis, interpretation and conclusions? Do these prohibit publication or require revision?

1.7 Overall the discussion is logically presented and the analysis and code are made available. There is no major flaw that should prevent publication.

Reply by Authors to 1.7. We thank the Reviewer for their support of our work.

Please consider the following points to enhance discussion and to render the content more accessible to non-modelers.

1.8 It would be helpful to include some discussion of whether there could be assumed Ov16-negative due to reasons other than diagnostic failure. For example, it has been observed that approximately 20% of skin snip positive elicit no detectable anti-Ov16 IgG4. This could be due to deficiency in IgG4, or lack of immune response to Ov16, in addition to a technical sensitivity deficiency in the ELISA or RDT.

Reply by Authors to 1.8. We thank the Reviewer for raising this important point. We have added a paragraph in the Discussion clarifying that Ov16-negative results among infected individuals can arise for reasons other than technical diagnostic failure, including heterogeneity in Ov16-specific IgG4 responses, infection-stage/titre effects, and incomplete antigenic "coverage" (i.e., antibody responses preferentially directed to other parasite antigens). We cite work on paediatric data showing that a subset of microfilaria-positive children had no detectable antibody to Ov16 (or other recombinant antigens) despite responses to crude parasite antigen, and we note that such findings do not by themselves demonstrate stable individual-level "non-responder" phenotypes (most evidence is cross-sectional). We also clarify in the Discussion that our sensitivity parameter is phenomenological—representing this composite process at the survey level—consistent with our modelling assumptions.

"Relatedly, an Ov16-negative result in an infected individual may reflect several processes, including variability in the magnitude and timing of the Ov16-specific IgG4 response, antigenic 'coverage' of the assay (i.e., individuals whose humoral response is dominated by reactivity to other parasite antigens), and technical performance of the ELISA or RDT. In a paediatric study from Sierra Leone,[29] only 75% of skin-snip positive children had detectable IgG to Ov16, and 10/53 microfilaria-positive children had no detectable IgG to any of four recombinant antigens (Ov11, Ov27, Ov29 or Ov16) despite low–moderate responses to crude adult worm antigen; the authors concluded that additional epitopes may be needed and noted that longitudinal follow-up would be required to determine the significance of these 'antibody-negative' infections. Likewise, contemporary

evaluations of Ov16 assays in endemic settings consistently report sensitivities below 100% among parasitologically-confirmed infections.[19,30,34,36] These observations are sometimes interpreted as evidence of stable 'non-responder' phenotypes; however, direct evidence for persistent individual-level failure to mount an Ov16-specific IgG4 response is limited because most data are cross-sectional and antibody responses can both lag behind infection and wane after clearance.[16,19] In our modelling, we interpret sensitivity as an effective probability that a truly infected individual is observed as being Ov16-positive at a given survey, subsuming these biological and technical sources of discordance rather than attributing them to fixed host phenotypes."

1.9 Please clarify why H4 (i) chosen over H4(ii). Although the minimum values at some sensitivity and specificities were in seroreversion hypothesis (i), across different seroconversion and different sensitivity and specificity values, it appeared that (ii) had more often a better MSE.

Reply by Authors to 1.9. In the original manuscript, H4i was the best fit for 1/9 diagnostic performance combinations, H4ii for 4/9, and H4iii for 4/9. Although H4iii had the smallest (best) overall mean squared error (MSE), the 95% confidence intervals (CIs, arising from stochastic variation) of the MSEs overlapped across hypotheses, including overlap between the smallest MSE for H4iii and the smallest MSE for H4i. We therefore retained H4i for validation with the serological data from Togo as an additional plausible hypothesis under uncertainty.

However, based on comments from Reviewer 2, we have updated our method to sample from distributions of diagnostic performance parameters (sensitivity/specificity), to better integrate uncertainty in diagnostic performance and enhance the robustness of our final conclusions. This updated method also yields a single MSE per seroconversion/seroreversion hypothesis while integrating uncertainty in diagnostic performance (the true value of which is not identifiable from these data, as described above; see **Reply by Authors to 1.6**). Under this approach, hypotheses H4ii and H4iii are superior to H4i, but are indistinguishable from each other (overlapping 95% CIs of the MSEs). Hence, we take both H4ii and H4iii forward to the validation stage of the analysis but do not carry H4i forward under the updated uncertainty integration.

1.10 Is there any diagnostic test bias introduced by seroreversion hypothesis – for example, if the reversion hypothesis more closely matches data, but in reality there is poorer sensitivity of the diagnostic test.

Reply by Authors to 1.10. We thank the Reviewer for bringing up this important point. We agree that, because seroreversion and diagnostic sensitivity are confounded, apparent support for a seroreversion hypothesis could reflect poorer-than-assumed test sensitivity rather than true antibody waning. We, therefore, do not infer diagnostic performance from goodness-of-fit. In the revised analysis, we integrate diagnostic uncertainty by sampling sensitivity and specificity from empirically-informed prior distributions and propagating this uncertainty through fitting/validation, which reduces the potential for diagnostic bias regarding inference about seroreversion (see also **Reply by Authors to 1.6**).

1.11 Is there any accommodation for differential performance of the diagnostic depending on the prevalence and treatment history – some concerns about the serological performance having poorer sensitivity in populations who have been under treatment for longer or are hypoendemic.

Reply by Authors to 1.11. We agree with the Reviewer that context-dependent performance of anti-Ov16 serology is plausible. In settings with prolonged MDA and/or low transmission intensity, true

infections may be associated with lower and more heterogeneous antibody responses (including seroreversion), increasing the probability that individuals fall near the assay cut-off and are classified as negative. Empirically, both anti-Ov16-ELISA/RDT sensitivity estimates and observed seroreversion patterns vary across settings and sampling platforms and are not expected to be constant. In our analysis, we do not attempt to infer how sensitivity varies with prevalence or treatment history. Rather, we treat diagnostic performance as uncertain and integrate this uncertainty by sampling sensitivity and specificity from empirically-informed distributions, so that our conclusions about seroconversion/seroreversion are robust to plausible ranges of effective test performance. A fuller accommodation of systematic, epidemiological setting and/or intervention history-dependent sensitivity (e.g., via explicit modelling of quantitative titres and assay cut-offs as functions of infection intensity and time since treatment) would require longitudinal titre data linked to independent infection measures across settings with different intervention histories, which were not available for the present study. We have added the following to the Discussion to clarify the approach we have taken:

> "In addition, diagnostic performance may plausibly vary across epidemiological contexts and intervention histories (e.g., if antibody titres are lower or more heterogeneous in hypoendemic settings or after prolonged MDA), which could contribute to setting-specific effective sensitivity. We do not model titre dynamics explicitly here and therefore treat sensitivity/specificity as uncertain effective parameters rather than attempting to infer context-dependent diagnostic performance characteristics from these data."

1.12 Given the wide confidence intervals for higher ages, could the validation be focused on younger age groups – those most important for establishing elimination?

Reply by Authors to 1.12. The wider confidence intervals at older ages arise primarily from smaller sample sizes for those ages. In our validation, we account for this through the weighted mean squared error (MSE) calculation. For a given model run, we compute the squared error between modelled and observed seroprevalence in each age group, and then weight each age-specific squared error by the corresponding observed age-group sample size before summing across ages to obtain a single MSE. Consequently, younger age groups—where sample sizes are larger and which are programmatically most relevant for elimination decisions—contribute more to the overall validation metric, while older ages are retained to ensure consistency with the full age-profile of seroprevalence. This approach allows us to validate hypotheses in a way that naturally emphasises younger ages without excluding older ages entirely.

• Is the methodology sound? Does the work meet the expected standards in your field?

1.13 Yes, the authors are attentive to best practice and standards set by modelling consortia.

Reply by Authors to 1.13. We thank the Reviewer for this positive comment and for appreciating our efforts to apply sound methodologies that follow best practice in infectious disease modelling.

1.14 It would be helpful to provide some context about the key finding that the near-patent/patent seroconversion may be driving the superior performance. Some combination with all seroconversion (especially with ii seroreversion) hypotheses produced low MSE instances. Can different conversion/reversion combinations produce similar results though some offset?

Reply by Authors to 1.14. We thank the Reviewer for this important point. Yes, different seroconversion/seroreversion assumptions can produce similar age-seroprevalence profiles through

partial offsetting. For example, faster seroreversion and/or lower effective sensitivity both increase the probability of observing Ov16-negative results among infected or exposed individuals, so these processes can compensate for one another to some extent (as noted in the Discussion section: "seroreversion and diagnostic sensitivity are confounded; higher sensitivity can compensate, to some extent, for declining antibody responses"). Likewise, earlier versus later seroconversion changes the timing at which children accumulate seropositivity, and this can be partially counterbalanced by different assumptions about seroreversion or effective test sensitivity, yielding occasional low-mean squared error instances under alternative combinations. We have revised the Discussion to clarify that although some seroconversion/seroreversion/sensitivity combinations can partially offset and yield similar fits for specific age groups, our conclusions are based on comparative performance across the sampled diagnostic-performance ranges using all-age data, under which the near-patent/patent seroconversion hypothesis provides the best overall fit. The revised section of the Discussion now reads:

> "A salient advantage of our analysis is the use of all-age seroprevalence data from Gabon[22], the integration of uncertainty in diagnostic performance, and the comparison of results when using age- and sex-dependent EPIONCHO and ONCHOSIM exposure functions. While some combinations of exposure, diagnostic performance and seroreversion can partially compensate and yield similar fits for specific age groups, the all-age data and uncertainty-integrated fitting framework show that, on average across the sampled parameter ranges, the near-patent/patent seroconversion hypothesis consistently outperforms hypotheses invoking pre-patent seroconversion."

• Is there enough detail provided in the methods for the work to be reproduced? (Remarks on code availability):

1.15 Yes, the authors have provided code for this model and supplemental information. I do not have the required expertise to check the code. The presence of the code on public website was confirmed.

Reply by Authors to 1.15. We appreciate the Reviewer's efforts in confirming our provision of the model code and methodological detail to ensure reproducibility of our analyses.

Reviewer #2 (Remarks to the Author):

The manuscript is well written and can be published once the authors have attended to the issues raised in the attached file.

Referee's Summary. I have read the manuscript in which the authors have extended EPIONCHO-IBM with an immunologically motivated Ov16 IgG4 seroconversion–seroreversion sub-model, calibrated to Gabon microfilarial age-prevalence and validated with long-running intervention data from Togo. The authors systematically compared hypotheses about the parasite stage that elicits seroconversion and found the near-patent/patent stage best explaining age–seroprevalence patterns across a range of diagnostic sensitivities/specificities. This is timely and policy-relevant for programs shifting from skin snips to serological surveillance. The manuscript is generally well structured and transparent about key assumptions such as prefecture-level histories, and lack of village-level series. The core result which is near-patent/patent exposure drives Ov16

seroconversion comes through clearly and is well defended by the cross-setting calibration/validation workflow.

Reply by Authors to Referee's Summary. We thank the referee for this positive summary of our work.

However, the authors should consider the following to improve the manuscript.

2.1 Name consistency and model identification: - There's an instance of EPONCHO-IBM21 (missing the "I"). Please correct to EPIONCHO-IBM for consistency and search for any other occurrences. This is in the Results header section ("We calibrated EPONCHOIBM21…").

Reply by Authors to 2.1. We thank the Reviewer for taking the time to highlight typos and grammatical/spelling errors. We have gone through and corrected the manuscript thoroughly.

2.2 Quantifying uncertainty and model comparison across hypotheses: - The authors report weighted MSEs and state that the near patent/patent hypothesis outperforms pre-patent hypotheses, but readers would benefit from the following:

2.2.1 Reporting uncertainty on the comparative metric for instance, bootstrap CIs on ΔMSE between hypotheses and/or an information criterion (WAIC/LOO-CV) to indicate strength of evidence, not just point MSE.

Reply by Authors to 2.2.1. We agree with the Reviewer that readers will benefit from uncertainty quantification around comparative fit metrics. We therefore report the 95% Monte Carlo confidence intervals (CIs) for the weighted MSEs (arising from stochastic variation across model runs; see **Reply by Authors to 2.3** below) and we now display these 95% Monte Carlo CIs in Fig. 3 (and continue to provide complete detail in the Supplementary Material file). We use overlap/non-overlap of these CIs to communicate the strength of evidence for differences in fit between hypotheses, rather than relying on point estimates only. We did not add information-criterion measures such as WAIC/LOO-CV because our model comparison is based on a simulation-based discrepancy (weighted MSE) rather than an explicit likelihood for the observed age-stratified seroprevalence; WAIC/LOO-CV are not directly applicable without specifying a probabilistic observation model. Likewise, while ΔMSE values can be reported relative to a chosen reference hypothesis, they do not alter the ranking/inference provided by the MSEs and their CIs, and our primary focus is therefore on uncertainty-integrated MSE comparisons. We have added a small clarification of our approach to communicating uncertainty in model fits in the revised Results:

> "Uncertainty in these comparisons is summarised using 95% Monte Carlo confidence intervals (95% MCCIs; integrating stochastic uncertainty) associated with the weighted MSEs."

2.2.2 Showing a compact table with ΔMSE (or ΔWAIC) relative to the best hypothesis for both Gabon (calibration) and Togo (validation), alongside assumed diagnostic Se/Sp values. This will crystallize robustness across diagnostic assumptions.

Reply by Authors to 2.2.2. We appreciate the Reviewer's suggestion. In the revised analysis, we integrate diagnostic uncertainty by sampling sensitivity and specificity from empirically-informed distributions (see **Reply by Authors to 2.3** below); therefore, each hypothesis yields a single diagnostic uncertainty-integrated weighted mean squared error (MSE) with 95% Monte Carlo confidence interval (which expresses stochastic uncertainty), rather than a set of MSEs indexed by discrete sensitivity/specificity values. To facilitate comparison, we present the hypotheses' MSEs in

normalized form relative to the best-performing hypothesis (i.e., ΔMSE scaled to the best fit) in Fig. 3 for Gabon (calibration) and Fig. 6 for Togo (validation). These figures provide the compact "ΔMSE-like" summary requested and demonstrate robustness under integrated diagnostic uncertainty. For transparency, the point-estimate / fixed sensitivity/specificity analyses are retained in the Supplementary Information.

2.3 Treatment of diagnostic performance (Se/Sp): - The authors currently examine discrete grids of Se/Sp for the RDT (Gabon) and HRP ELISA (Togo). Consider (a) placing Bayesian priors on Se/Sp and integrating them to obtain posterior predictive fits, or (b) Monte-Carlo drawing Se/Sp from empirically informed distributions rather than fixed points. This would propagate diagnostic uncertainty consistently into predicted age seroprevalence and the hypothesis comparison.

Reply by Authors to 2.3. We thank the Reviewer for this insightful suggestion. We have revised the main analysis to propagate diagnostic uncertainty by Monte Carlo sampling sensitivity and specificity from empirically-informed beta distributions, rather than evaluating only a discrete grid of fixed values. Specifically, we parameterise beta distributions for sensitivity and specificity (separately for the Gabon Ov16-RDT and Togo HRP Ov16-ELISA) using published mean/variance estimates; we then draw sensitivity/specificity pairs repeatedly and apply these draws to adjust model-predicted 'true' seroprevalence to 'observed' seroprevalence, and compute weighted mean squared errors (MSEs) that are averaged (marginalised) over the diagnostic performance uncertainty. This yields uncertainty-integrated MSEs (with 95% Monte Carlo CIs that capture residual stochastic uncertainty) for each hypothesis. We emphasise that sensitivity and specificity are treated as uncertain inputs and are not inferred from goodness-of-fit in our analysis (see **Reply by Authors to 1.6**).

2.4 Calibration details & reproducibility: - The authors weight squared errors by age-group sample proportions which is good, yet the precise objective function and optimization/search mechanics are only referenced to Supplementary Methods. Add the explicit formula and any hyper-parameters in the main text (or a short box) and provide a minimal script/notebook in the repo that recreates Fig. 1 calibration and the hypothesis testing loop (1,500 stochastic repeats, ABR–k_E combinations). Also ensure the exact analysis scripts (not only the general EPIONCHO-IBM code) are linked in "Code availability". The authors currently duplicate the GitHub link; replace with a single link and a path to the analysis folder.

Reply by Authors to 2.4. We thank the Reviewer for these detailed and constructive suggestions to strengthen reproducibility. We have substantially revised the main text to include the explicit weighted MSE objective function (and mathematical definitions of all terms, including the age-group weights), with additional implementation details retained in Supplementary Methods. We have also revised the Code availability section to (i) remove duplicated links and (ii) point directly to the analysis code used to generate the results in this manuscript, which will resolve to a released version of the model.

To further support reproducibility, we have added a step-by-step reproducibility entry point in the repository that enables readers to rerun the core workflow underlying Fig. 1 calibration and the hypothesis-testing loop (including the 1,500 stochastic repeats and ABR–k_E combinations). This is achieved using a README to describe each of the files that need to be used to run the model, process the model outputs, and create the outputs shown in the paper. Additionally, the final, processed model outputs are made available so that users do not have to re-run the model to reproduce the results, as we acknowledge that the model's run time is substantial.

We have clarified the following in the Code availability statement, where we indicate that instructions for reproducing the results in the paper are provided:

> "The EPIONCHO-IBM model code and instructions to replicate the analyses conducted in this study are available at: https://github.com/mrc-ide/EPIONCHO.IBM/tree/ov16. Instructions on how to use the code are contained within the accompanying README file.
>
> The version of the EPIONCHO-IBM model with the best-fit hypotheses from this paper is available at: https://github.com/mrc-ide/EPIONCHO.IBM/tree/master. Steps on how to use the model code are given in the vignettes."

2.5 Validation in Togo: granularity and known heterogeneity: - The authors acknowledge prefecture-level aggregation might drive over-prediction in Ôti/Kpendjal (vector control likely near 100% in some villages, higher than your 90% cap). It would help to:

2.5.1 Run a sensitivity analysis lifting the 90% cap to 100% and show its influence on Ôti/Kpendjal predictions.

Reply by Authors to 2.5.1. We thank the Reviewer for this suggestion. We agree that lifting the assumed upper bound on vector-control efficacy provides a useful upper-bound sensitivity analysis to assess whether our prefecture-level aggregation could be contributing to over-prediction in Ôti/Kpendjal. We therefore repeated the relevant validation analyses allowing vector control efficacy to reach 100% for the enhanced intervention scenario in the Ôti/Kpendjal prefeture. The resulting outputs were not substantially different from the original analyses, and did not change our conclusions. We have included the MSE results in Supplementary Tables 11 and 13 and referred to them in the revised discussion:

> "As an additional robustness check, we repeated the Togo validation allowing vector-control efficacy to reach 100% in the enhanced scenario for Ôti/Kpendjal; however, the results were not substantially different and did not affect our principal conclusions (Supplementary Tables 11 and 13)."

We emphasise, however, that the intent of this analysis is not to assert that 100% vector control efficacy was achieved uniformly. Village-level analyses presented in Amaral et al. (*Nat Commun* 2025; 17: 779) suggest that best-fitting village-specific scenarios frequently fall below 100% (e.g., 60–90%), although the assumption of 100% vector control efficacy was also simulated and presented in some figures, e.g. Fig. 2 (page 4) of Amaral et al. (op. cit.). Accordingly, we interpret the 100% scenario as an extreme bound on plausible heterogeneity.

2.5.2 Provide prefecture-specific posterior ABR distributions pre/post vector control as a figure (densities or violin plots) to visualize how much headroom the model has to reconcile data.

Reply by Authors to 2.5.2. We thank the Reviewer for the suggestion to add the posterior ABR distributions for Togo. We have added these as Supplementary Figure 3.

2.5.3 If feasible, a small "what-if" panel quantifying how village-level heterogeneity (plausible variance in coverage and VC efficacy) affects predicted seroprevalence.

Reply by Authors to 2.5.3. We thank the Reviewer for this suggestion. Our validation analysis already incorporates village-level heterogeneity to the extent supported by the available data: baseline transmission intensity is represented using an ABR distribution, and for Togo we compiled village-specific intervention scenarios (coverage, systematic non-adherence, and vector-control

efficacy categories) and propagated this variability when simulating prefecture-level outcomes (see Supplementary Methods 1 and Supplementary Methods 2). However, the observed serological outcomes available for validation are aggregated at the prefecture level, and we do not have age-stratified village-level seroprevalence to quantify the variance in predicted seroprevalence attributable specifically to village-level heterogeneity in a way that would be identifiable. We therefore focus on prefecture-level validation, while noting village-level heterogeneity as a likely contributor to residual misfit (particularly in Ôti/Kpendjal).

2.6 Age-exposure structure and children: - The authors note EPIONCHO-IBM assumes higher exposure in <10y and that this could overestimate seroprevalence in children compared with ONCHOSIM structures. They can consider an additional scenario with a moderated child exposure profile to demonstrate whether their central conclusion (near-patent/patent driver) is robust to this structural choice. A simple figure showing the two exposure profiles and resulting fits in children would pre-empt critiques.

Reply by Authors to 2.6. We thank the Reviewer for this helpful suggestion. We agree that the assumed age-exposure structure, particularly in children, is an important structural uncertainty. We have therefore extended the analysis to include an alternative age- and sex-dependent exposure profile consistent with the ONCHOSIM framework in addition to the default EPIONCHO-IBM (which we refer to as EPIONCHO exposure function). We now include a comparison of the two exposure functions in Supplementary Figure 1. We recalibrated the model to the Gabon all-age seroprevalence dataset under both exposure structures and repeated the hypothesis comparisons. This shows that our central conclusion is robust: across both exposure profiles, seroconversion initiated by near-patent/patent infection (H4) provides the best overall fit to the Gabon age-seroprevalence patterns (with H4ii/H4iii remaining the leading seroreversion variants). The use of two exposure functions (EPIONCHO and ONCHOSIM) to enhance robustness to structural model uncertainty is now integrated fully throughout the analyses.

2.7 Entomological dynamics and secular trends: - The authors state that, absent long entomological series, only vector control was assumed to reduce ABR; climate/environmental secular trends were not represented. Add a brief sensitivity where ABR drifts by a small annual percentage (±1–2%/year) independently of VC to show the conclusion is not an artefact of constant-baseline assumptions.

Reply by Authors to 2.7. We thank the Reviewer for this suggestion. We performed the requested sensitivity analysis by allowing the pre-intervention ABR to drift independently of vector control by a small annual percentage (±1–2% per year) over the relevant period. This produced no appreciable change in the predicted age-seroprevalence trajectories or in the hypothesis ranking/weighted MSEs. The reason is that the Togo settings modelled for validation are characterised by very high baseline transmission intensity (high ABR/endemicity), such that small secular perturbations of the ABR within this narrow range do not materially change the force of infection or the resulting serological age profiles compared with the dominant effects of intervention history and the seroconversion/seroreversion assumptions. Because the resulting curves were visually indistinguishable from the constant-ABR assumption and the inference was unchanged, we have not added an additional figure or table. We also refer the reviewer to the paper by Amaral et al. (Nat Commun 2025; 17: 779), where the issue of secular trends in ABR (or lack thereof) is discussed in detail for Togo, with examples given. See Limitations section (page 7) of Amaral et al. (op. cit.).

2.8 Clarity on within-host vs vector dynamics: - The methods nicely describe life-stage tracking and deterministic handling of mf dynamics while stochastic at the host level. A concise schematic (or expanded caption) clarifying which components are stochastic vs deterministic, and where density

dependence enters (especially "establishment increases as transmission decreases") would help readers who are less familiar with EPIONCHO-IBM's architecture.

Reply by Authors to 2.8. We thank the Reviewer for their suggestion. We have included a schematic (new Fig. 8) that conveys the EPIONCHO-IBM current architecture and makes explicit the deterministic and stochastic components of the model.

2.9 Figure suggestions: - For each multi-panel age-seroprevalence figure, add: (i) explicit sample sizes per age bin in the caption, (ii) the assumed Se/Sp values used, (iii) the number of stochastic repeats, and (iv) a concise legend mapping H4i vs H4iii line styles. Authors already include many of these; consolidating them in every relevant caption will aid skim readers.

Reply by Authors to 2.9. We thank the Reviewer for this helpful presentation suggestion. In the revised manuscript we have standardised the captions for all multi-panel age-seroprevalence figures to explicitly include: (i) the total sample size; (ii) the diagnostic sensitivity/specificity assumptions used (or that sensitivity and specificity are drawn from distributions, with means specified); (iii) the number of stochastic repeats, and (iv) a concise legend clarifying hypothesis labels (e.g., H4ii/H4iii) and line styles. We retain the plotted Wilson score 95% CIs on observed seroprevalence estimates as error bars, but have ensured that the requested metadata are consistently provided in the captions/legends. This also is good practice and indeed required by *Nat Commun*.

2.10 Code and data availability: - Collapse the duplicated GitHub URL lines into one and point to a specific commit/tag used for this paper (e.g., a release). This makes the work reproducible and avoids link rot.

Reply by Authors to 2.10. We thank the Reviewer for this suggestion. We have provided two links to the GitHub repository to give access to two separate branches of the modelling analysis. One link takes users to the run scripts and analysis files that were used to produce this paper. The second link directs users to a cleaned model code that provides the final version of the model integrating, within EPIONCHO-IBM, the final results of our analysis (see **Reply by Authors to 2.4**).

2.11 There are several typographical and grammatical errors. The authors should check the following and others not highlighted.

2.11.1 "EPONCHO-IBM21" → EPIONCHO-IBM. Also check for any "EpiOncho vs EPIONCHO" capitalization inconsistencies throughout.

2.11.2 "microfilarie increases" → microfilariae increase (plural noun + plural verb).

2.11.3 "serovonversion" → seroconversion which has been captured multiple instances in Discussion/Conclusions.

2.11.4 "cross sectional studies" → cross-sectional studies (add hyphen).

Reply by Authors to 2.11. We thank the referee for this meticulous check and have corrected all typographical errors.

2.12 Numerical clarity: - Where the authors give sampling proportions (e.g., 324/1455 = 22%), add one decimal place consistently or give exact percentages with the same rounding rule across prefectures. Currently the percentages are mixed precision; consider standardizing to one decimal.

Reply by Authors to 2.12. We thank the Reviewer for this suggestion. We have standardized all reported values in the manuscript to be rounded to 3 significant figures where we have calculated

the values ourselves. Values reported by others have been left with their existing precision. We have added the following text towards the end of the Methods section:

> "All reported values were rounded to 3 significant figures with the exception of those values taken from the literature, which are given with their reported precision."

ROUND 1 REVIEWER 2 ATTACHMENT:

Summary

I have read the manuscript in which the authors have extended EPIONCHO-IBM with an immunologically motivated Ov16 IgG4 seroconversion–seroreversion sub-model, calibrated to Gabon microfilarial age-prevalence and validated with long-running intervention data from Togo. The authors systematically compared hypotheses about the parasite stage that elicits seroconversion and found the near-patent/patent stage best explaining age–seroprevalence patterns across a range of diagnostic sensitivities/specificities. This is timely and policy-relevant for programs shifting from skin snips to serological surveillance. The manuscript is generally well structured and transparent about key assumptions such as prefecture-level histories, and lack of village-level series. The core result which is near-patent/patent exposure drives Ov16 seroconversion comes through clearly and is well defended by the cross-setting calibration/validation workflow. However, the authors should consider the following to improve the manuscript.

1. Name consistency and model identification: - There's an instance of *EPONCHO-IBM21* (missing the "I"). Please correct to *EPIONCHO-IBM* for consistency and search for any other occurrences. This is in the Results header section ("We calibrated EPONCHO-IBM21…").
2. Quantifying uncertainty and model comparison across hypotheses: - The authors report weighted MSEs and state that the near patent/patent hypothesis outperforms pre-patent hypotheses, but readers would benefit from the following
    - Reporting uncertainty on the comparative metric for instance, bootstrap CIs on ΔMSE between hypotheses and/or an information criterion (WAIC/LOO-CV) to indicate strength of evidence, not just point MSE.
    - Showing a compact table with ΔMSE (or ΔWAIC) relative to the best hypothesis for both Gabon (calibration) and Togo (validation), alongside assumed diagnostic Se/Sp values. This will crystallize robustness across diagnostic assumptions.
3. Treatment of diagnostic performance (Se/Sp): - The authors currently examine discrete grids of Se/Sp for the RDT (Gabon) and HRP ELISA (Togo). Consider (a) placing Bayesian priors on Se/Sp and integrating them to obtain posterior predictive fits, or (b) Monte-Carlo drawing Se/Sp from empirically informed distributions rather than fixed points. This would propagate diagnostic uncertainty consistently into predicted age-seroprevalence and the hypothesis comparison.
4. Calibration details & reproducibility: - The authors weight squared errors by age-group sample proportions which is good, yet the precise objective function and optimization/search mechanics are only referenced to Supplementary Methods. Add the explicit formula and any hyper-parameters in the main text (or a short box) and provide a minimal script/notebook in the repo that recreates Fig. 1 calibration and the hypothesis-testing loop (1,500 stochastic repeats, ABR–k_E combinations). Also ensure the exact analysis scripts (not only the general EPIONCHO-IBM code) are linked in "Code availability". The authors currently duplicate the GitHub link; replace with a single link and a path to the analysis folder.
5. Validation in Togo: granularity and known heterogeneity: - The authors acknowledge prefecture-level aggregation might drive over-prediction in Ôti/Kpendjal (vector control likely near 100% in some villages, higher than your 90% cap). It would help to:

- Run a sensitivity analysis lifting the 90% cap to 100% and show its influence on Ôti/Kpendjal predictions.
- Provide prefecture-specific posterior ABR distributions pre/post vector control as a figure (densities or violin plots) to visualize how much headroom the model has to reconcile data.
- If feasible, a small "what-if" panel quantifying how village-level heterogeneity (plausible variance in coverage and VC efficacy) affects predicted seroprevalence.

6. Age-exposure structure and children: - The authors note EPIONCHO-IBM assumes higher exposure in <10y and that this could overestimate seroprevalence in children compared with ONCHOSIM structures. They can consider an additional scenario with a moderated child exposure profile to demonstrate whether their central conclusion (near-patent/patent driver) is robust to this structural choice. A simple figure showing the two exposure profiles and resulting fits in children would pre-empt critiques.
7. Entomological dynamics and secular trends: - The authors state that, absent long entomological series, only vector control was assumed to reduce ABR; climate/environmental secular trends were not represented. Add a brief sensitivity where ABR drifts by a small annual percentage (±1–2%/year) independently of VC to show the conclusion is not an artefact of constant-baseline assumptions.
8. Clarity on within-host vs vector dynamics: - The methods nicely describe life-stage tracking and deterministic handling of mf dynamics while stochastic at the host level. A concise schematic (or expanded caption) clarifying which components are stochastic vs deterministic, and where density dependence enters (especially "establishment increases as transmission decreases") would help readers who are less familiar with EPIONCHO-IBM's architecture.
9. Figure suggestions: - For each multi-panel age-seroprevalence figure, add: (i) explicit sample sizes per age bin in the caption, (ii) the assumed Se/Sp values used, (iii) the number of stochastic repeats, and (iv) a concise legend mapping H4i vs H4iii line styles. Authors already include many of these; consolidating them in every relevant caption will aid skim readers.
10. Code and data availability: - Collapse the duplicated GitHub URL lines into one and point to a specific commit/tag used for this paper (e.g., a release). This makes the work reproducible and avoids link rot.
11. There are several typographical and grammatical errors. The authors should check the following and others not highlighted.
    - "EPONCHO-IBM21" → EPIONCHO-IBM. Also check for any "EpiOncho vs EPIONCHO" capitalization inconsistencies throughout.
    - "microfilarie increases" → microfilariae increase (plural noun + plural verb).
    - "serovonversion" → seroconversion which has been captured multiple instances in Discussion/Conclusions.
    - "cross sectional studies" → cross-sectional studies (add hyphen).
12. Numerical clarity: - Where the authors give sampling proportions (e.g., 324/1455 = 22%), add one decimal place consistently or give exact percentages with the same rounding rule across prefectures. Currently the percentages are mixed precision; consider standardizing to one decimal.

ROUND 2 REVIEWER 3 ATTACHMENT:

Thank you for giving me the opportunity to serve as a mediator. I am grateful to you for giving me the opportunity to make a few comments to the authors on their manuscript entitled "Modelling anti-Ov16 seroprevalence for the control and elimination of onchocerciasis".

As a mediator, I reviewed the authors' responses to the reviewer's comments, as expected. I also made comments to the authors.

According to the letter detailing the responses to the reviewers' comments, I noted that Reviewer 1 had made 15 points, consisting of 7 comments and 8 questions. The authors have responded to all of these.

Regarding the comments, the authors thanked reviewer 1 for the comments and appreciated his comment made for his interest in their study.

Regarding the questions, the authors also answered all the questions (1.2., 1.6, 1.8, 1.9, 1.10, 1.11, 1.12, 1.14). For each question, they provided a satisfactory response. They also incorporated the changes made to address the various questions into the manuscript, taking a great care to highlight them.

Therefore, we validate the responses provided by the authors to the reviewer's questions.

I would like to congratulate the authors on this essential work in the elimination of onchocerciasis. This work will certainly form the basis for the future studies, as once the effective serological tools are available for surveys, this study will incorporate these new parameters and advise on new achievable thresholds for decision-making needs of different countries. I have made some comments in the manuscript for the authors on certain points. They can judge whether these comments are relevant to the current manuscript or to other future studies.

I reiterate my sincere thanks for choosing me to be a mediator, and I remain available for any further contributions in the future.

Very Best regards,

Achille S. Nikièma
