# Supplementary material for: Modelling anti-Ov16 seroprevalence for the control and elimination of onchocerciasis
Source: Nat Commun. 2026 Aug 19;17:9924. doi: 10.1038/s41467-026-76562-9 (PMC13582928; doi:10.1038/s41467-026-76562-9)
Supplement: Supplementary file 3 [file 41467_2026_76562_MOESM3_ESM.pdf]

nature portfolio

Corresponding author(s): Maria-Gloria Basáñez, Martin Walker

Last updated by author(s): Jul 9, 2026

Reporting Summary

Nature Portfolio wishes to improve the reproducibility of the work that we publish. This form provides structure for consistency and transparency in reporting. For further information on Nature Portfolio policies, see our Editorial Policies and the Editorial Policy Checklist.

Statistics

For all statistical analyses, confirm that the following items are present in the figure legend, table legend, main text, or Methods section.

n/a	Confirmed	
☐	☒	The exact sample size (*n*) for each experimental group/condition, given as a discrete number and unit of measurement
☐	☒	A statement on whether measurements were taken from distinct samples or whether the same sample was measured repeatedly
☒	☐	The statistical test(s) used AND whether they are one- or two-sided<br>*Only common tests should be described solely by name; describe more complex techniques in the Methods section.*
☒	☐	A description of all covariates tested
☐	☒	A description of any assumptions or corrections, such as tests of normality and adjustment for multiple comparisons
☐	☒	A full description of the statistical parameters including central tendency (e.g. means) or other basic estimates (e.g. regression coefficient) AND variation (e.g. standard deviation) or associated estimates of uncertainty (e.g. confidence intervals)
☐	☒	For null hypothesis testing, the test statistic (e.g. *F*, *t*, *r*) with confidence intervals, effect sizes, degrees of freedom and *P* value noted<br>*Give P values as exact values whenever suitable.*
☒	☐	For Bayesian analysis, information on the choice of priors and Markov chain Monte Carlo settings
☒	☐	For hierarchical and complex designs, identification of the appropriate level for tests and full reporting of outcomes
☒	☐	Estimates of effect sizes (e.g. Cohen's *d*, Pearson's *r*), indicating how they were calculated

Our web collection on statistics for biologists contains articles on many of the points above.

Software and code

Policy information about availability of computer code

Data collection	No software was used to collect the data analysed in this manuscript. The data from the hypoendemic (treatment-naive) setting in Gabon were provided by colleagues at the Task Force for Global Health and a description of data collection is provided in Atsame et al. (2024) Data in Brief. The data from Togo were obtained from the published paper by Komlan et al. (2018) PLoS Negl Trop Dis. These sources of data are fully described and cited in the manuscript.
Data analysis	All model simulations were run using R (version 4.4.3; R Core Team. R: A language and environment for statistical computing. R Foundation for Statistical Computing, Vienna, Austria. https://www/R-project.org/ (2025)) and the Imperial College Research Computing Service (Imperial College Research Computing Service. Imperial College London, UK. https://doi.org/10.14469/hpc/2232 (2025)).<br><br>The code used to run the model, analyse the results, and produce outputs are available on Github (https://github.com/mrc-ide/EPIONCHO.IBM/tree/ov16), and through Zenodo (https://doi.org/10.5281/zenodo.21244449).

For manuscripts utilizing custom algorithms or software that are central to the research but not yet described in published literature, software must be made available to editors and reviewers. We strongly encourage code deposition in a community repository (e.g. GitHub). See the Nature Portfolio guidelines for submitting code & software for further information.

Data

Policy information about availability of data

All manuscripts must include a data availability statement. This statement should provide the following information, where applicable:

- Accession codes, unique identifiers, or web links for publicly available datasets
- A description of any restrictions on data availability
- For clinical datasets or third party data, please ensure that the statement adheres to our policy

The manuscript includes a Data Availability statement: "All information used for the analyses is contained in the figures, tables, and Supplementary Information. The data used to calibrate and validate the model are publicly available in the publications by Atsame et al. 2024, Komlan et al. 2018 and Amaral et al. The following link describes the source data and the code to reproduce the analyses presented: https://doi.org/10.5281/zenodo.21244449.", with references to each study given within the text.

Research involving human participants, their data, or biological material

Policy information about studies with human participants or human data. See also policy information about sex, gender (identity/presentation), and sexual orientation and race, ethnicity and racism.

Reporting on sex and gender	We do not report results by sex or gender, but do report results by age (age profiles of Onchocerca volvulus microfilarial prevalence and anti-Ov16 seroprevalence).
Reporting on race, ethnicity, or other socially relevant groupings	We do not report results by race and/or ethnicity. The primary data were collected in onchocerciasis-endemic communities in Gabon and Togo, and hence refer to African populations.
Population characteristics	We describe the number of individuals, their age range, the diagnostics used to assess infection (skin-snip microscopy for microfilariae) and Ov16 assays for the assessment of IgG4 antibodies against the O. volvulus Ov16 antigen (Rapid Diagnostic Tests or Enzyme-Linked Immunosorbent Assays). We also provide information on the treatment history of the populations which were examined to obtain the primary data (by other authors), namely, treatment-naive in Gabon, and four decades of ivermectin treatment (mass drug administration) in Togo. We also simulated the impact and duration of vector control as an additional intervention in Togo where appropriate.
Recruitment	N/A. This is a modelling study using publicly available data.
Ethics oversight	The published studies that collected the primary data provide the details of the Ethics Review Boards that approved the studies. We have included an Ethics statement as follows: "This study analysed, through transmission dynamics modelling, de-identified, individual-level and aggregate village-level microfilarial prevalence and anti-Ov16 IgG4 seroprevalence data collected in Gabon and Togo in 2015. Ethical approval for these studies was obtained, respectively, from the Ministry of Health and Social Affairs of the Republic of Gabon (APOC/EVE/030/15/HZ)22, and the Togolese Bioethics Committee for Research in Health (Comité de Bioéthique pour la Recherche en Santé; CBRS (#013/2015/CBRS/3.Septembre 2015)23. The study in Togo was also granted study authorization and approval by the Ministry of Health of Togo (#338/2015/MSPS/CAB/SG)23. For our (in silico) study, no human participants were recruited and no individual-level identifiable data were used; therefore, no institutional review board approval and informed consent were required for the present (modelling) analysis."

Note that full information on the approval of the study protocol must also be provided in the manuscript.

Field-specific reporting

Please select the one below that is the best fit for your research. If you are not sure, read the appropriate sections before making your selection.

☒ Life sciences ☐ Behavioural & social sciences ☐ Ecological, evolutionary & environmental sciences

For a reference copy of the document with all sections, see nature.com/documents/nr-reporting-summary-flat.pdf

Life sciences study design

All studies must disclose on these points even when the disclosure is negative.

Sample size	We provide information on the sample sizes that were used in the primary data collection studies. For Gabon: 4,350 individuals (aged 5-85 years, from 67 communities) with skin-snip microscopy; of these, 4,257 (from 58 communities) with blood samples for the Ov16 Rapid Diagnostic Test (Ov16 RDT). For Togo: 1,455 individuals (aged 5-80 years, from 11 villages in 4 prefectures) with skin-snip microscopy; of these 644 tested using Horseradish Peroxidase Ov16 Enzyme-linked Immunosorbent Assay (HRP Ov16 ELISA). Data on age (for age-seroprevalence profiles) were available from the Komlan et al. 2018 publication for 576 people.
Data exclusions	No data were excluded.
Replication	The model was simulated 1,500 times for 100 years for each of the questions assessed. The predicted 'true' seroprevalence from each run of the model was adjusted using 1,500 pairs of diagnostic sensitivity and specificity values. Full details are provided in the Methods section of the manuscript, and the Supplementary Methods section of the Supplementary Material.
Randomization	N/A

Blinding	N/A

Reporting for specific materials, systems and methods

We require information from authors about some types of materials, experimental systems and methods used in many studies. Here, indicate whether each material, system or method listed is relevant to your study. If you are not sure if a list item applies to your research, read the appropriate section before selecting a response.

Materials & experimental systems

n/a	Involved in the study
☐	☒ Antibodies
☒	☐ Eukaryotic cell lines
☒	☐ Palaeontology and archaeology
☒	☐ Animals and other organisms
☒	☐ Clinical data
☒	☐ Dual use research of concern
☒	☐ Plants

Methods

n/a	Involved in the study
☒	☐ ChIP-seq
☒	☐ Flow cytometry
☒	☐ MRI-based neuroimaging

Antibodies

Antibodies used	The Ov16 serology assays (RDT and ELISA) test for the presence of IgG4 antibodies against the O. volvulus Ov16 antigen.
Validation	The use of IgG4 has already been validated in previous studies for the purposes of assessing the presence of the Ov16 antigen. We provide references to these studies: Lobos et al. An immunogenic Onchocerca volvulus antigen: a specific and early marker of infection. Science 251, 1603–1605 (1991); Weil et al. A rapid-format antibody card test for diagnosis of onchocerciasis. J. Infect. Dis. 182, 1796–1799 (2000); Cama et al. Evaluation of an Ov-16 IgG4 enzyme-linked immunosorbent assay in humans and its application to determine the dynamics of antibody responses in a non-human primate model of Onchocerca volvulus infection. Am. J. Trop. Med. Hyg. 99, 1041–1048 (2018).

Plants

Seed stocks	N/A
Novel plant genotypes	N/A
Authentication	N/A
